# Bridging Chemical Literature and Molecular Graphs: LLM‐Augmented Enantioselectivity Prediction for Iridium‐Catalyzed Asymmetric C–H Borylation

**DOI:** 10.1002/advs.78094

**Published:** 2026-10-14

**Authors:** Zhe Wang, Baohua Zhang, Yi Isaac Yang, Yuan Yao, Yinwu Li

**Affiliations:** ^1^ School of Materials and New Energy South China Normal University Shan Wei China; ^2^ Hong Kong University of Science and Technology Hong Kong China; ^3^ Shenzhen Bay Laboratory Shen Zhen China; ^4^ Shenzhen Medical Academy of Research and Translation Shen Zhen China

**Keywords:** borylation, LLM, machine learning, spatial molecular graph, stereoselectivity

## Abstract

Asymmetric C–H borylation provides direct access to enantioenriched organoboron compounds. Predictive catalyst design for this reaction class remains challenging, as stereocontrol arises from coupled interactions among catalyst, substrate, and product structures. Moreover, mechanistic knowledge is dispersed across the literature. Here we report a machine‐learning framework for predicting enantioselectivity in iridium‐catalyzed asymmetric C–H borylation that bridges molecular graphs and chemical literature. A unified dataset comprising 800 literature‐reported reactions was constructed for this reaction class. The model combines a multi‐entity three‐dimensional graph encoder for catalyst, substrate, and product with temperature‐aware kinetic treatment, and a large‐language‐model prior module that extracts atom‐resolved mechanistic annotations from primary publications. On a scaffold‐based held‐out test set, the STEREO‐Lit (Spatial Three‐Entity Reaction Encoder Literature) model achieves strong predictive performance, outperforming conventional machine‐learning baselines and a geometry‐grounded control. Perturbation studies further show that literature‐derived semantic annotations contribute chemically meaningful, site‐specific information rather than merely increasing model capacity. The trained model was subsequently applied to explore a reaction space of about 100 000 catalyst–substrate combinations and identify promising high‐improvement candidates, several of which were supported by DFT calculations. The framework couples multi‐entity 3D encoding with literature‐derived priors, and remains predictive when training data are limited to a few hundred reactions.

## Graphical abstract

A machine‐learning framework for asymmetric C–H borylation bridges multi‐entity 3D molecular graphs and chemical literature. Atom‐resolved mechanistic annotations extracted by large language models augment three‐component graph encoding of catalyst, substrate, and product. The model predicts enantioselectivity with high accuracy on unseen scaffolds and prospectively screens ∼100 000 catalyst–substrate combinations, with DFT validation of selected candidates.

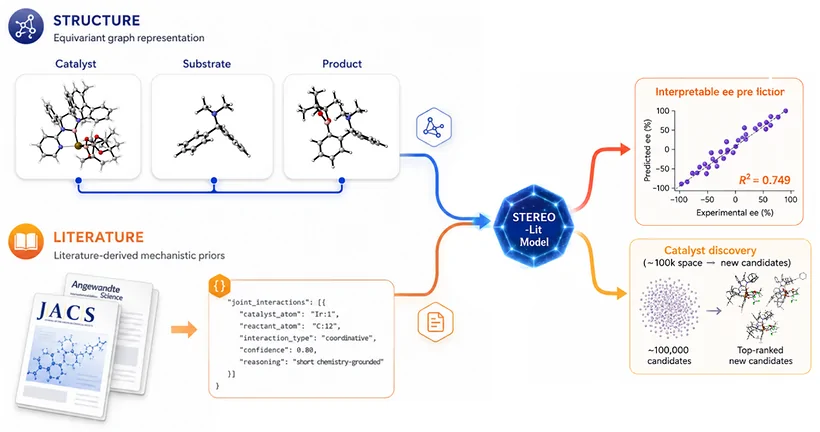

## 1 Introduction

Asymmetric C–H borylation provides a direct catalytic route to enantioenriched organoboron compounds [1, 2]. These compounds are key intermediates for the construction of C─C, C─N, and C─O bonds (Figure 1A) [3, 4]. Such motifs are valuable in medicinal chemistry, as exemplified by the chiral boronic‐acid drug bortezomib, underscoring the significance of stereocontrolled C–H borylation. Through stereospecific oxidation, amination, cross‐coupling, and related C─B bond transformations, such intermediates provide rapid access to densely functionalized chiral scaffolds relevant to synthesis, medicinal chemistry, and materials science [5]. Iridium‐based catalysts have proven particularly effective for asymmetric C–H borylation, operating under mild conditions and accommodating a broad substrate scope [6, 7]. Their selectivity can be tuned by steric effects, directing groups, and secondary interactions in both C(sp^2^)–H and C(sp^3^)–H substrates when chiral ligands such as BINOL‐derived monophosphites, quinolyl oxazolines, and N,B‐bidentate frameworks are used [8, 9, 10, 11]. Despite this progress, experimental data remain scattered across independent studies [4]. No unified dataset is available for this reaction class. As a result, rational catalyst design for new substrate families remains difficult because enantiocontrol arises from a delicate interplay of multiple factors.

**FIGURE 1:**
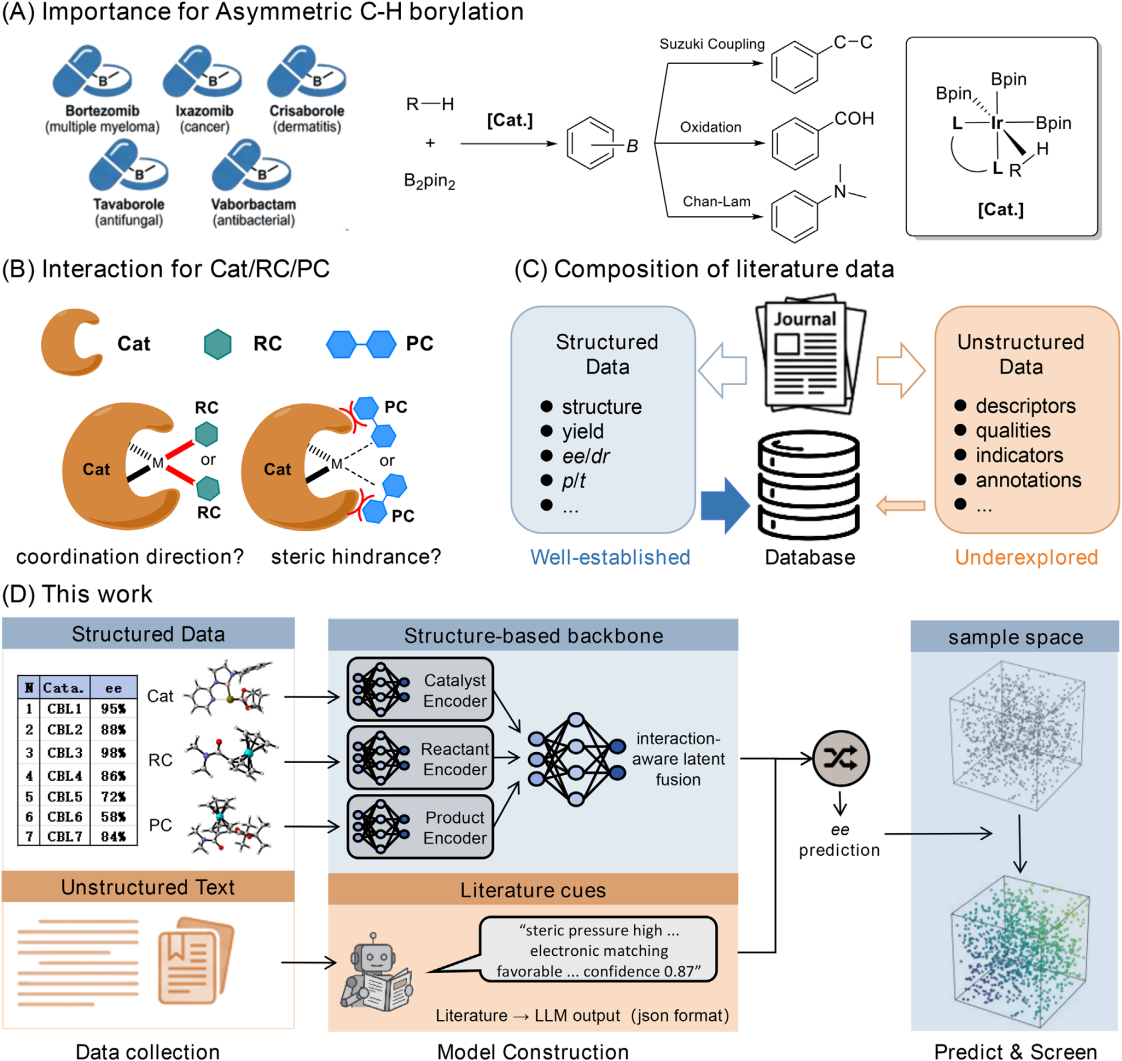
Schematic overview of literature‐augmented multi‐entity learning for asymmetric C‐H borylation. (A) strategic importance of asymmetric C‐H borylation. (B) three‐component interactions among catalyst, substrate, and product. (C) Composition of literature data: structured and unstructured. (D) This work.

Recent work has clarified ligand effects [9, 12, 13, 14] and extended asymmetric Ir‐catalyzed borylation to diverse substrate classes, including strained (hetero)cycloalkanols, aminocyclopropanes, ether‐directed substrates, benzhydryl ethers, axially chiral N‐aryl phthalimides, and sulfoximines. These systems reveal that weak coordination, local steric confinement, and ligand side‐arm effects can combine to enforce high enantiocontrol [15, 16, 17, 18, 19, 20]. Directed enantioselective borylation has reached aryl chlorides and P‐stereogenic phosphorus compounds, further broadening the design space for catalyst‐controlled selectivity [21, 22, 23]. Empirical optimization alone, however, cannot disentangle the multiple factors that control selectivity [24]. Density functional theory can resolve these interactions at the transition‐state level, but systematic screening remains computationally prohibitive because both the catalyst and the substrate are conformationally flexible [25]. Machine learning therefore offers an attractive alternative [26]. By learning structure‐selectivity relationships directly from experimental data, ML models avoid costly transition‐state calculations [27, 28]. A growing body of work shows that machine‐learning models, leveraging diverse molecular representations spanning high‐throughput experimental data, literature reaction libraries, catalytic‐cycle intermediates, and tunable fragment descriptors, enable robust prediction of enantioselectivity and rational design of chiral catalysts and reaction conditions [29, 30]. These data‐driven strategies circumvent expensive quantum chemical calculations, allow the discovery of highly selective systems from limited training data, and reveal previously unrecognized structural and environmental factors governing stereoselectivity [31, 32, 33, 34, 35, 36].

Despite this promise, current ML approaches still struggle with the structural complexity of asymmetric C–H borylation [37]. Most models rely on two‐dimensional molecular representations [38]. These representations discard the stereochemical information required to distinguish prochiral faces [34]. More importantly, catalyst, substrate, and product are usually encoded as independent feature vectors [39], ignoring the intermolecular compatibility that dictates selectivity in the transition state [27, 40, 41]. The electronic structure and spatial configuration of the reactants jointly govern their interaction mode with the catalyst. Meanwhile, the spatial structure of the products exerts steric effects on the catalyst through the transition state (Figure 1B). Another limitation lies in the mechanistic knowledge embedded in the primary literature (Figure 1C) [42]. Structured data, including molecular structures, activity metrics, and reaction conditions, can be readily used for model construction [43]. However, the literature also contains unstructured text that records mechanistic reasoning from prior studies [37]. Purely geometric encodings cannot access this implicit knowledge [42]. Models trained only on three‐dimensional structures can learn which spatial arrangements correlate with high enantioselectivity, but they cannot explain why a specific electronic environment at a particular atomic position is chemically decisive [25]. Recent efforts to incorporate language‐model‐derived information into molecular prediction point in a promising direction [39]. Examples include entity‐level embeddings in drug–target interaction studies and literature‐mined descriptors in catalysis [44, 45, 46]. However, these approaches still operate at a coarser granularity than the atom‐resolved and reaction‐specific priors that govern enantioselectivity.

Here, we address these gaps by integrating multi‐entity three‐dimensional graph learning with literature‐derived semantic knowledge (Figure 1D). We assembled 800 asymmetric C–H borylation reactions from the published literature and established a unified dataset for this reaction class, to our knowledge, the first such dataset. This dataset provides the empirical foundation for subsequent modeling. Our structure‐based backbone represents the catalyst, substrate, and product as jointly optimized 3D molecular graphs. It fuses their embeddings through a shared learnable projection that couples their spatial and electronic complementarity at the latent level. To capture the unstructured layer of mechanistic reasoning, we introduce a large language model prior module. This semantic layer extracts atom‐resolved annotations from the primary literature and dynamically directs the model's attention to selectivity‐determining sites. The model therefore does not need to infer these relationships solely from structural data. This unified framework combines geometry‐based learning with literature‐encoded chemical intuition. It enables prediction with both quantitative accuracy and mechanistic interpretability. By integrating multi‐entity structural inputs with language‐model‐derived textual knowledge, this work provides a potential strategy for building machine‐learning models for asymmetric catalysis under small‐data conditions, although its transferability beyond iridium‐catalyzed C‐H borylation remains to be verified and is presented here as a hypothesis for future work.

## 2 Result and Discussion

### 2.1 Overview of the Modeling Framework

Our workflow comprises three stages. First, we curated a unified dataset of enantioselective Ir‐catalyzed C–H borylation reactions from the primary literature. Each reaction was represented as a catalyst–substrate–product triplet, and the experimental *ee* value was used as the prediction target. Next, we built a two‐tier predictive model. The structure‐based model encodes the three molecular components as 3D graphs and couples them with a temperature‐aware kinetic module. The semantic layer extracts atom‐resolved mechanistic priors from the source publications by using a large language model. Finally, we used the trained model to screen an expanded chemical space of untested catalyst–substrate combinations. Selected predictions were further validated by DFT calculations. We refer to this structure‐based model as STEREO (Spatial Three‐Entity Reaction Encoder), and to its literature‐augmented full model as STEREO‐Lit.

### 2.2 Dataset Curation and Reaction Space Analysis

Enantioselective Ir‐catalyzed borylation reactions were systematically collected from the published literature, yielding a curated dataset of 800 reactions. The dataset spans 174 catalysts and 606 reaction components. We partitioned the dataset into training, validation, and test sets with a split of 640, 80, and 80. We used a scaffold‐based structural‐signature splitting strategy to ensure that reactions sharing catalyst, substrate, or product scaffolds did not appear in more than one partition. This design allows the model to be evaluated on genuinely new combinations rather than on interpolation within known scaffold families.

The *ee* distribution across substrate classes is strongly skewed toward high selectivity, with a median value of 90.0% *ee* (Figure 2A), reflecting the publication bias of asymmetric catalysis toward successful examples. It also increases the difficulty of prediction because distinguishing between 88% *ee* and 95% *ee* requires the model to resolve numerically small but practically decisive differences. The catalyst generality plot in Figure 2B shows that a small subset of catalyst scaffolds is reused across dozens of substrates while maintaining high *ee* values. By contrast, other catalysts are confined to narrow substrate scopes. This pattern highlights the many‐to‐many coupling between catalyst and substrate families in this reaction class.

**FIGURE 2:**
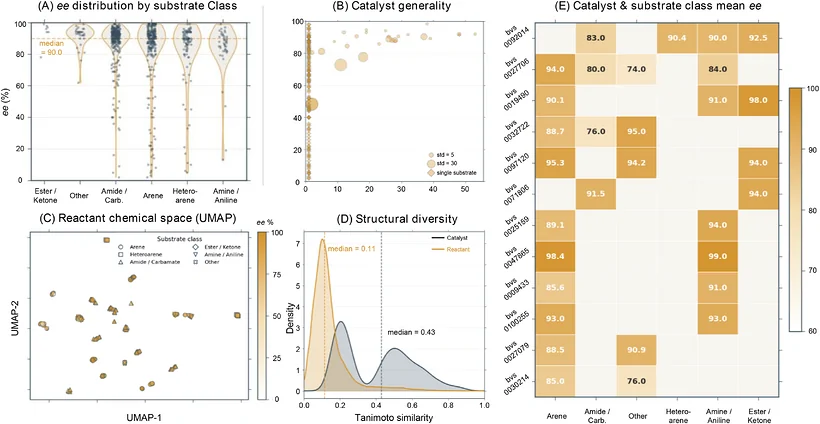
Dataset curation and chemical space analysis. (A) *ee* distribution across substrate class. (B) catalyst generality plot. (C) UMAP [47] projection of reactant chemical space. (D) structural diversity analysis. (E) catalyst–substrate class heat map of mean *ee* values. The catalyst identifiers (bvs 00…) are internal database codes used solely for indexing within this dataset (Table S1).

The UMAP projection of reactant chemical space in Figure 2C confirms the structural diversity of the substrate families. Partial clustering is observed by class, but substantial overlap remains between neighboring categories. The Tanimoto similarity [48] distributions in Figure 2D reveal a strong asymmetry between catalysts and substrates. Catalysts are structurally concentrated, with a median pairwise similarity of approximately 0.43, indicating that a relatively small number of privileged scaffolds dominate the catalyst pool. Substrates are much more dispersed, with a median similarity of approximately 0.11. The catalyst–substrate class heat map in Figure 2E further shows that the same catalyst family can deliver markedly different *ee* values across different substrate classes, and the same trend is observed in the opposite direction. Selectivity therefore cannot be regarded as an intrinsic property of either component alone.

These analyses impose two key constraints on model design. First, *ee* in this system emerges from the joint geometric and electronic fit among the catalyst, substrate, and product. The model therefore requires multi‐entity encoding rather than single‐molecule featurization. Second, the structural concentration of the catalyst pool means that the model must learn transferable interaction patterns across catalyst–substrate pairings rather than memorizing individual scaffolds.

### 2.3 Predictive Performance of the Multi‐Entity Framework

With these constraints in mind, we evaluated the framework on a held‐out test set of 80 reactions. STEREO‐Lit achieved an *R* ^2^ of 0.749 with a mean absolute error (MAE) of 5.19% *ee*, outperforming a panel of conventional baselines including Random Forest, Gradient Boosting, Support Vector Regression, k‐Nearest Neighbors, Ridge, ElasticNet, and a multilayer perceptron (MLP), all trained and evaluated on the identical scaffold‐based train/validation/test partition [49]. The strongest conventional baseline, Random Forest, reached *R* ^2^ = 0.584 (MAE = 5.9% *ee*); the gap is larger in *R* ^2^ than in MAE, suggesting that the advantage of STEREO‐Lit is concentrated in the harder, high‐error cases rather than spread uniformly across the test set. To place this error in context, a constant predictor fixed at the training‐set mean *ee* gives a test MAE of 10.12% *ee* (test‐label SD 16.27), a 49% higher error than STEREO‐Lit's 5.19% *ee*. On the 31 of 80 held‐out reactions below 90% *ee*, STEREO‐Lit reaches 7.06% versus 12.29% for that baseline, confirming that its advantage holds precisely in the low‐selectivity regime where a mean predictor fails. The MLP baseline relied on concatenated descriptor representations rather than explicit multi‐entity 3D encoding. It reached only *R* ^2^ = 0.432, a shortfall that points to the input representation rather than to the use of a neural model per se. As a three‐dimensional reference, a SchNet model [50] that encodes catalyst, substrate, and product but merges them into a single graph reached *R* ^2^ = 0.545 (MAE = 5.6% *ee*), below even Random Forest, indicating that 3D geometry on its own is not sufficient. Enantioselectivity here arises from the three‐dimensional interplay among three chemically distinct components. Taken together, these comparisons indicate that STEREO's advantage stems from role‐separated, multi‐entity spatial encoding, rather than from descriptor choice, model capacity, or 3D geometry alone. The additional gain of STEREO‐Lit over STEREO is instead attributable to the literature‐derived semantic layer.

STEREO (the geometry‐grounded base, LLM layer removed) gave *R* ^2^ = 0.655, and the mean absolute error increased to 5.5% *ee*, as shown in Figure 3A. The drop indicates that literature‐derived annotations provide information beyond what can be captured by the three‐dimensional representation alone. The scatter plot of predicted versus observed *ee* values in Figure 3B shows that most test reactions fall within ±10% *ee* of the diagonal (Pearson *r* = 0.875). The largest deviations occur mainly in the lower‐*ee* regime, where the training data are comparatively sparse. Overall, the model preserves useful resolution across the full selectivity range while maintaining strong performance in the synthetically most relevant high‐*ee* region. As a further control, the JSON‐formatted reaction annotations were supplied to LLM without structure‐based encoding, yielding lower accuracy (*R* ^2^ = 0.62) than both STEREO and STEREO‐Lit. This confirms that the framework's advantage arises not from the parametric knowledge of the language model itself but from coupling multi‐entity 3D encoding with literature‐derived semantic priors.

**FIGURE 3:**
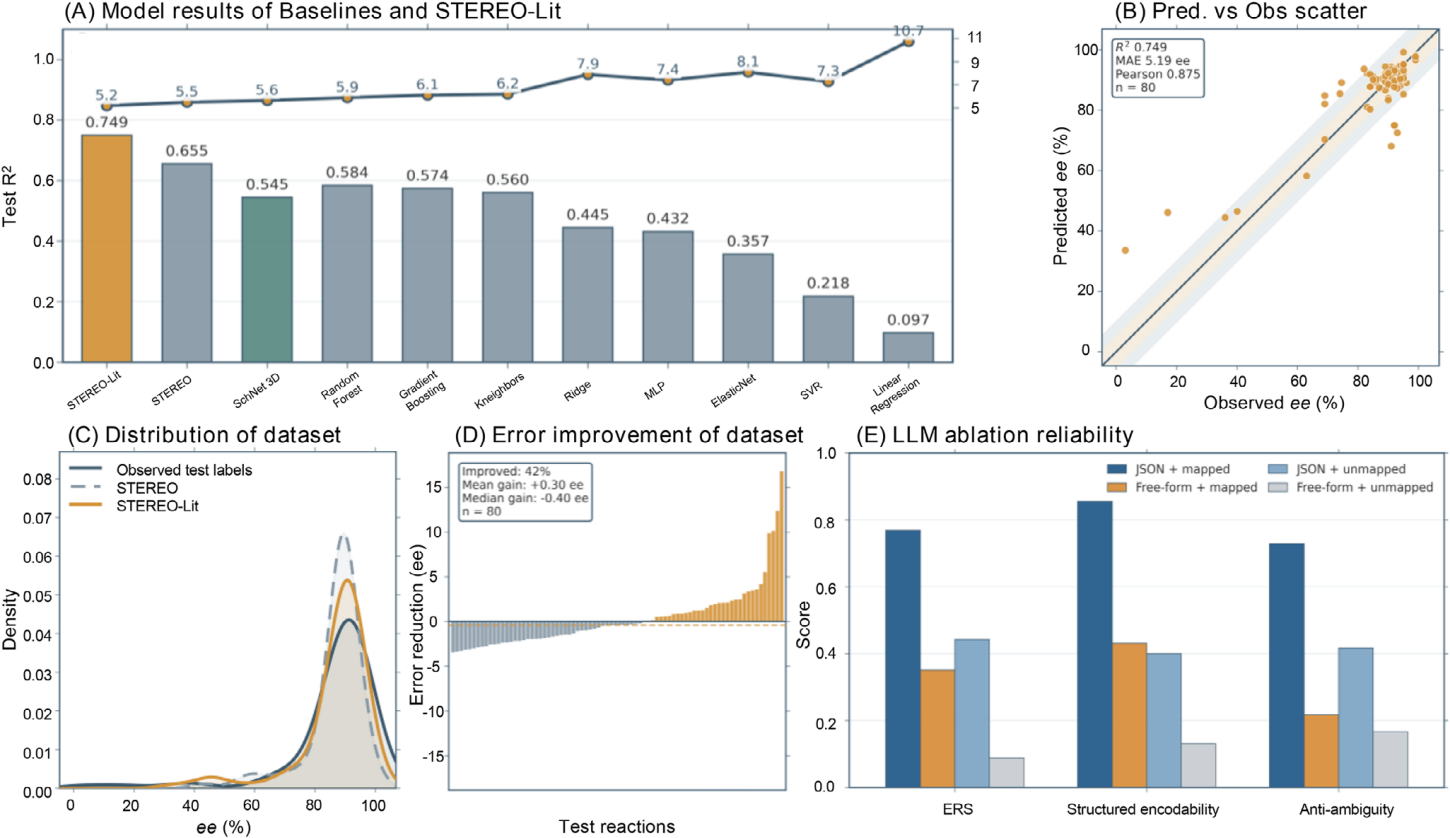
Performance evaluation against baselines and semantic controls. (A) model results comparison. (B) predicted versus observed ee scatter plot. (C) density distributions of predicted and experimental ee values. (D) reaction‐level absolute error improvement upon LLM semantic conditioning. (E) LLM ablation and extraction reliability metrics.

Comparison of the predicted density with the experimental distribution in Figure 3C reveals a qualitative difference between the two model variants. STEREO‐Lit reproduces the observed high‐*ee* peak faithfully. By contrast, STEREO smooths out the 90% to 100% *ee* window. This is exactly the regime in which the distinction between a good catalyst and an excellent catalyst is most important. The reaction‐level error analysis in Figure 3D further clarifies this effect. Each bar represents the change in absolute prediction error after literature conditioning. Positive values indicate that the semantic layer reduces the error, whereas negative values indicate that it worsens the prediction. The per‐reaction changes are heterogeneous: about 42% of reactions improve, the median shift is slightly negative (−0.4% *ee*), and the mean error reduction is +0.3% *ee*. Rather than a uniform shift, the gain is concentrated in a minority of high‐error reactions, where semantic conditioning corrects large structure‐only failures; for the majority of reactions the semantic branch leaves predictions essentially unchanged. This selective‐correction pattern is the intended behavior of the confidence‐gated fusion: the gate contributes only where literature evidence is informative, which is also why the improvement appears in *R* ^2^ (sensitive to large errors) more than in MAE. Additionally, the LLM ablation experiments and the extraction reliability analysis are shown in Figure 3E.

### 2.4 Semantic Validity of the LLM Annotations

The ablation analysis above shows that the semantic branch improves performance, but it does not reveal whether the LLM contributes meaningful chemical information or merely serves as an auxiliary feature channel. To isolate the effect of semantic content, we performed a text‐replacement experiment in which the molecular structures were kept fixed while only the LLM‐derived annotations were altered (Figure 4). For each test reaction, the original JSON annotation extracted from the corresponding source publication was replaced with an annotation randomly sampled from a reaction belonging to a different substrate class and a different control‐mode category. Because the molecular graphs and temperature were unchanged, any difference in predicted *ee* must arise from the semantic input alone.

**FIGURE 4:**
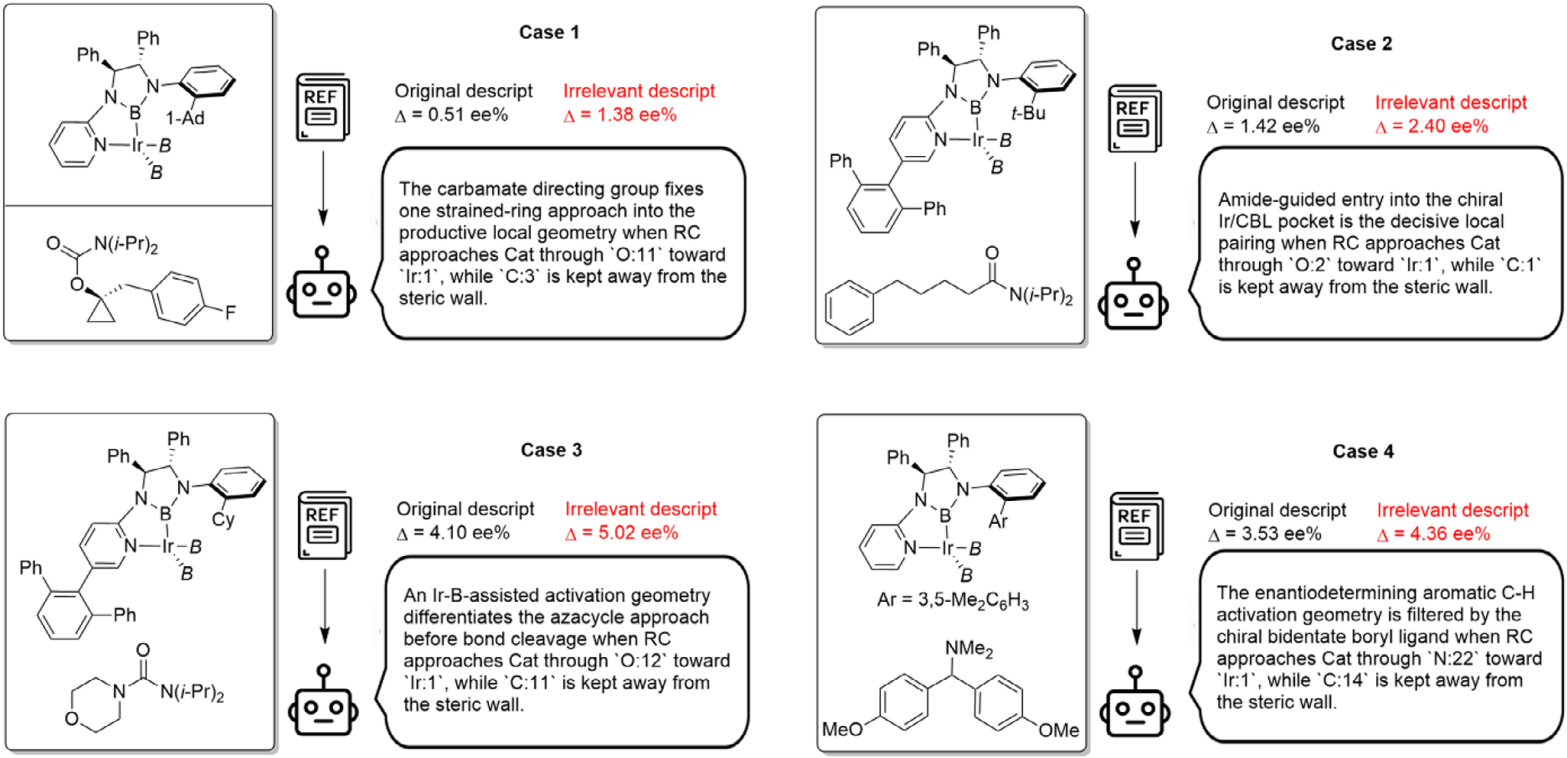
Perturbation analysis of semantic priors.

As shown in Figure 4, in Cases 1–3, the model identifies distinct oxygen‐directed coordination geometries as the enantiocontrolling local motifs: the carbamate directing group in Case 1 fixes one strained‐ring approach into the productive local geometry through O:11 toward Ir:1 while C:3 is kept away from the steric wall; the amide‐guided entry in Case 2 channels the substrate into the chiral Ir/ligand pocket through O:2 toward Ir:1 while C:1 is kept away from the steric wall; and the Ir‐B‐assisted activation geometry in Case 3 differentiates the azacycle approach through O:12 toward Ir:1 while C:11 is kept away from the steric wall. These atom‐specific annotations yield prediction errors of Δ = 0.51%, 1.42%, and 4.10% *ee*, respectively. Replacing them with unrelated descriptors while keeping the molecules unchanged increases the errors to Δ = 1.38%, 2.40%, and 5.02% *ee*, underscoring the decisive role of directed oxygen coordination in stereocontrol. In Case 4, the annotation instead describes a nitrogen‐directed aromatic C–H activation geometry filtered by the chiral bidentate boryl ligand, where RC approaches Cat through N:14 toward Ir:1 while N:14 is kept away from the steric wall, yielding Δ = 3.53% *ee*. Substituting this N:14‐centered activation mode with a generic descriptor raises Δ to 4.36% *ee*, confirming the model's dependence on atom‐specific coordination priors for enantioselectivity.

This experiment isolates the semantic channel from all other model inputs. The sharp drop in prediction accuracy upon annotation replacement confirms that the gate is not simply exploiting an additional parameter budget; it is selectively utilizing the chemical content of the LLM output. When that content is replaced by mismatched annotations, the prediction degrades substantially, consistent with the design intent of the confidence‐gated fusion mechanism described in the Methods section.

### 2.5 Chemical Space Exploration and Computational Consistency Check

Building on the validated *ee* prediction model, we extended its application to a broader reaction space. As shown in Figure 5A, 174 catalysts and 606 reaction components define a sample space of 105 444 combinations, of which only about 1% have been experimentally reported. We therefore used the best‐performing STEREO‐Lit model to predict *ee* values across the unobserved space. The resulting prediction landscape is summarized in Figure 5B as a function of distance from the training data in the conditioned latent space, and the same predictions are projected by *t*‐SNE [51] in Figure 5C (annotation‐free candidates receive a learned default semantic prior rather than candidate‐specific literature cues; see Methods). Because the experimental data are unevenly distributed and enriched in the high‐*ee* regime, we did not prioritize candidates solely by absolute predicted *ee*. Instead, we screened for catalyst replacement recommendations predicted to improve *ee* by more than 5 percentage points relative to an observed anchor reaction under the same substrate/product/temperature context, which yielded approximately 5,000 high‐improvement extrapolation candidates. Because each recommendation inherits the substrate, product, and thus the borylation site from its experimentally observed anchor, the screen does not require the model to predict regiochemistry for unseen catalysts; the assumption that the directing‐group‐controlled site is retained upon catalyst replacement within the same ligand family is consistent with the mechanistic precedents in the source literature. To enable mechanistic follow‐up, we further restricted attention to a literature‐supported subset of approximately 1670 candidates for which transition‐state models could be constructed in a chemically consistent manner based on reported mechanistic precedent. From this manageable subset, candidates were prioritized by three practical criteria: the predicted *ee* improvement over the original result, the prior experimental validation of the catalyst family in related reactions, and ensuring structural diversity among the proposed catalysts. For DFT analysis, we then selected three representative catalyst‐replacement recommendations to form a chemically comparable validation panel within the diarylphosphinate substrate family under the same temperature regime. Details of model construction and the structural optimization comparison under gas‐phase and solvent conditions are shown in Figures S2 and S3. This design was chosen to keep mechanisms comparable across transition‐state models, and to test whether our model‐selected catalysts still gave the same rescue effect for related substrates and catalyst families. Based on previously reported computationally optimized structures, we constructed transition‐state models for these three representative cases while maintaining consistency with the established reaction mechanism and favored conformational motifs of this system [23]. As shown in Figure 5D, the computed rate‐determining transition‐state energy differences for candidate recommendations 1–3 were 2.8, 4.5, and 8.1 kcal/mol, respectively. The reported *ee* values for these three substrates in the original literature were 84.0%, 88.0%, and 88.0%, respectively. The predicted *ee* values for the new combinations were 93.9%, 93.1%, and 94.2%, respectively.

**FIGURE 5:**
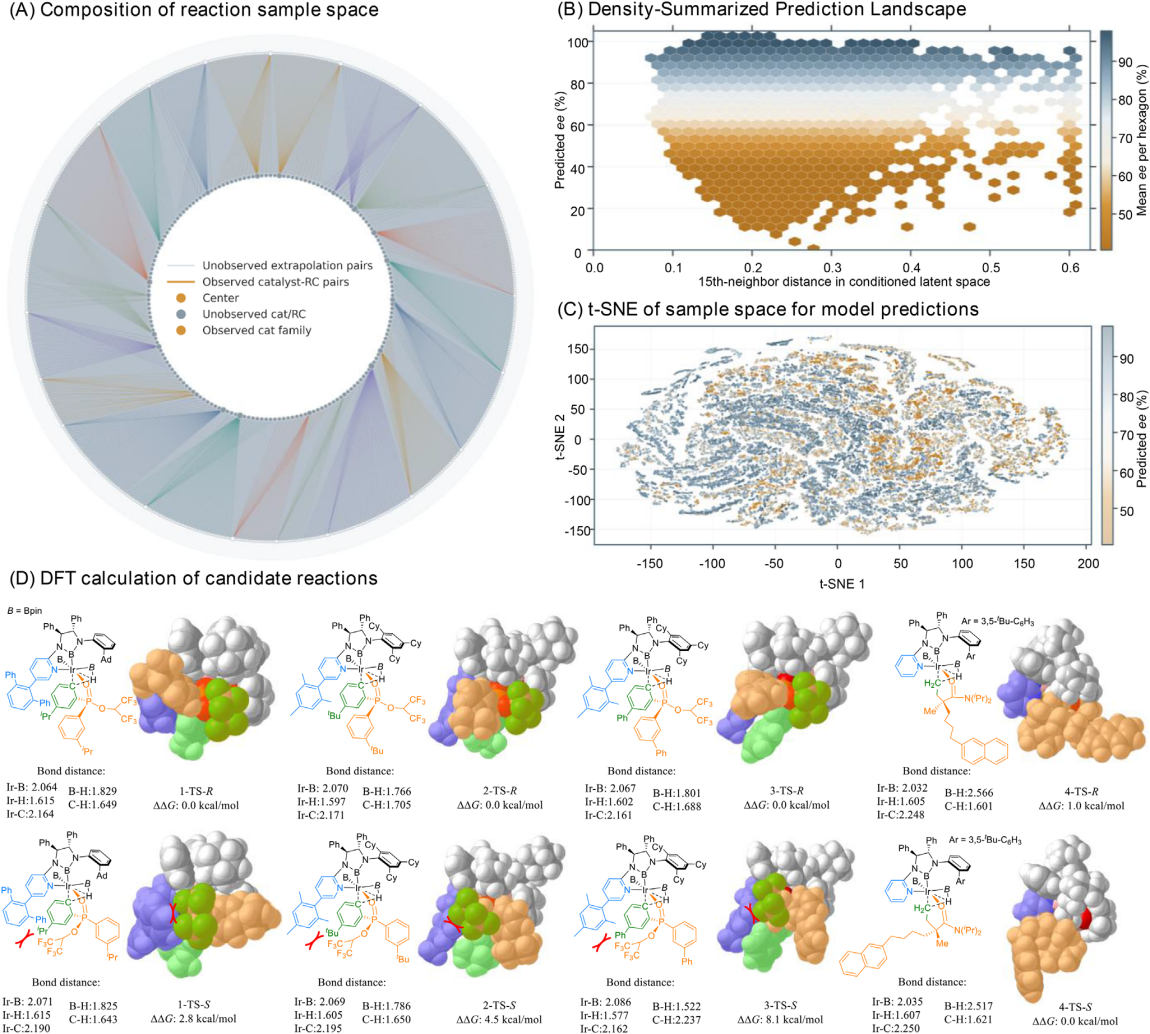
Extrapolation screening and computational validation. (A) composition of the reaction sample space. (B) density‐summarized prediction landscape. (C) *t*‐SNE projection of predicted sample space. (D) DFT validation of candidate reactions, the bond distances are shown in Å.

These calculations provide partial and qualitative support for the mechanistic plausibility of the model‐prioritized catalyst‐replacement recommendations rather than a quantitative confirmation: because *ee* is related to the transition‐state energy difference through the logarithmic Eyring relation, the computed ΔΔ*G* ^‡^ values in this regime correspond to *ee* values close to saturation and are experimentally indistinguishable, so no finer ranking can be inferred among the three cases. As a further test in a discriminating regime, a catalyst‐replacement candidate on a distinct amide substrate with a predicted *ee* of 69% was subjected to DFT transition‐state analysis. As shown in Figure 5D, the computed ΔΔ*G* ^‡^ for 4‐TS of 1.0 kcal/mol is quantitatively distinguishable from the high‐selectivity cases, supporting the model's ability to separate low‐ from high‐selectivity candidates. We further note that this validation covers only a small number of substrates and that additional experimental and DFT validation on further substrate families will be necessary to fully verify and iteratively update the model. The present results should therefore be interpreted as partial and qualitative.

## 3 Method

### 3.1 Multi‐Entity 3D Reaction Encoder

The distributional and structural analyses above indicate that enantioselectivity in this system is an emergent property of three‐component complementarity that geometry‐only single‐molecule representations cannot capture. We therefore designed a two‐tier architecture (Figure 6A): a structure‐based model encoding multi‐entity spatial compatibility under reaction conditions, and a literature‐derived semantic layer recovering atom‐resolved mechanistic priors from source publications.

**FIGURE 6:**
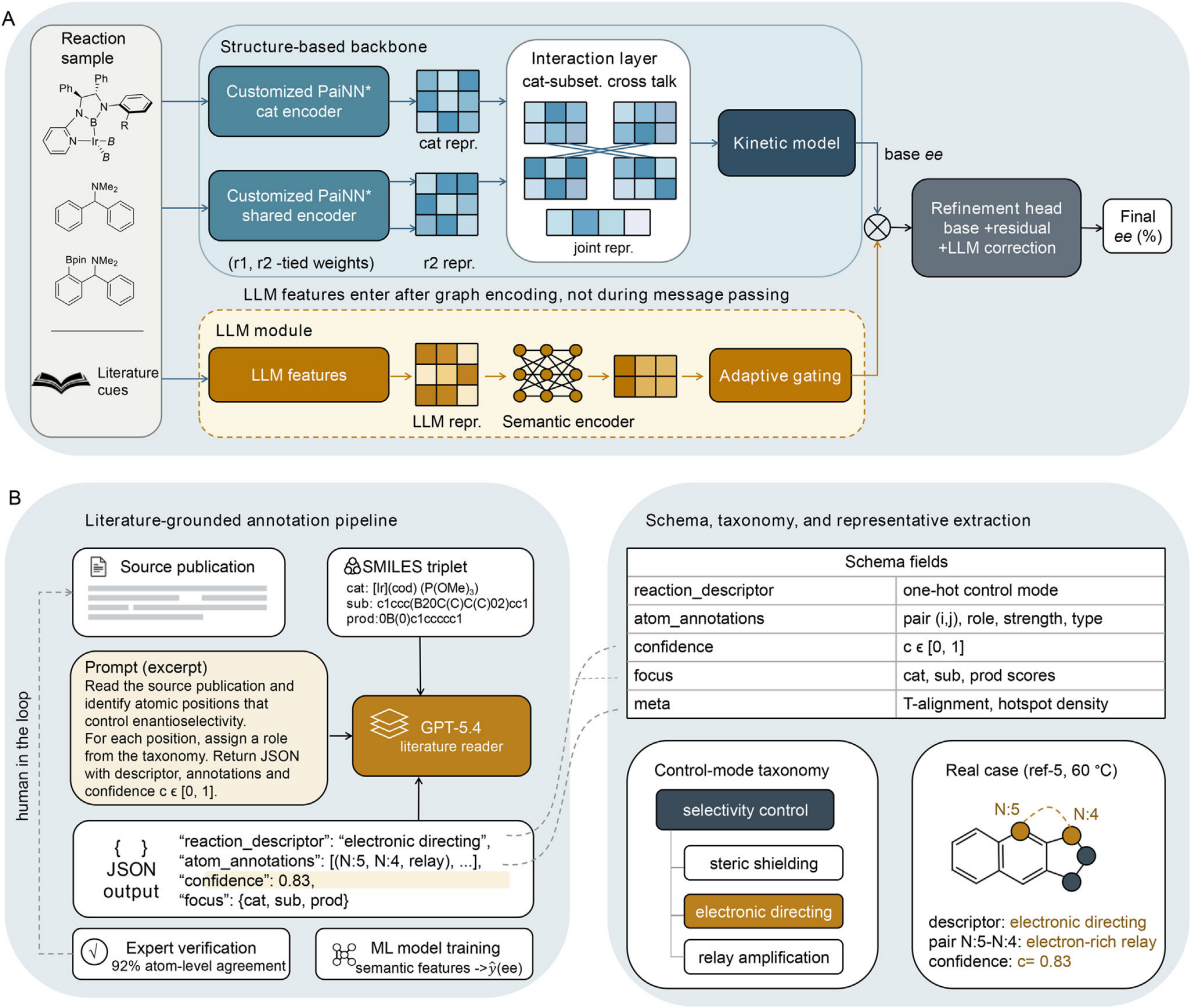
Architecture of the structure‐based model and semantic layer. (A) Multi‐entity 3D reaction encoder with PaiNN backbones and temperature‐aware kinetic coupling. (B) LLM‐derived mechanistic annotation pipeline with JSON schema and atom‐map grounding.

In asymmetric C–H borylation, the catalyst provides the chiral pocket, the substrate presents the prochiral site, and the product records the local steric environment resulting at the borylation position. These three components play chemically distinct roles in determining facial selectivity and cannot be collapsed into a single graph without losing role‐specific information. The product graph is not a proxy for the prediction target: its three‐dimensional geometry is fully determined by the substrate scaffold and the known reaction site, and is therefore available prior to any selectivity measurement. Including it lets the encoder capture how the spatial environment at the borylation position changes upon C═B bond formation. Enantioselectivity prediction in the present framework presupposes an established borylation site. The product geometry is constructed from the substrate scaffold and the known reaction position, so the model targets directed borylation reactions where activation sites are set by directing groups or established regioselectivity precedents. Joint prediction of regio‐ and enantioselectivity, which would require dedicated training labels and an evaluation protocol for site selection, is left to future work. We encode each component as an independent three‐dimensional molecular graph using PaiNN [52], an E(3)‐equivariant message‐passing neural network operating on atomic coordinates. We term this multi‐entity, structure‐based model STEREO. The three graph‐level embeddings are fused through a learnable function:

(1) $$h_{\mathrm{rxn}\;}=f_{\theta }\left(h_{\mathrm{cat}}\;⊕h_{\mathrm{sub}}\;⊕h_{\mathrm{prod}}\right)$$

where *h*_*cat*_, *h*_*sub*_, *h*_*prod*_ are the invariant graph‐level embeddings, and the ⊕ denotes concatenation and *f_θ_* is a shared nonlinear projection into a reaction‐level latent vector. Enantiomeric excess reflects the free‐energy difference ΔΔ*G* ^‡^ between competing transition states, which depends on temperature through the Eyring equation [53]. We therefore condition *h_rxn_* on a small kinetic module that ingests temperature and related descriptors.

### 3.2 Literature‐Derived Mechanistic Annotation via LLM

The structure‐based model captures spatial compatibility and kinetic modulation but cannot access the causal reasoning preserved in the primary literature. Large language models can parse such arguments from unstructured text but cannot compute transition‐state energies or sample conformational space; the challenge is therefore to extract LLM‐readable mechanistic knowledge and embed it into the spatial encoding framework without replacing the physics, while suppressing three failure modes‐lexical instability, hallucination, and lack of atom‐resolved granularity.

To address these difficulties simultaneously, we designed a structured extraction pipeline built around two coupled design choices: a fixed JSON schema and explicit atom‐map grounding (Figure 6B). For each reaction, the source publication was provided to GPT‐5.4 [54] together with the SMILES triplet of catalyst, substrate, and product, in which every heavy atom carries a stable identifier (e.g., N:5, C:9). Prior to extraction, explicit *ee* values and outcome‐revealing selectivity statements were manually redacted from the source text so that the semantic annotations reflected mechanistic rationale rather than reported labels. The prompt instructs the model to use these identifiers when referring to selectivity‐relevant positions, rather than free‐form spatial language such as “the nearby nitrogen” or “the carbon adjacent to the borylation site.” The output is constrained to a fixed JSON schema with five fields (Supporting Information): a reaction‐level selectivity descriptor drawn from a closed control‐mode taxonomy (steric shielding, electronic directing, relay amplification, etc.); atom‐resolved mechanistic annotations indexed by atom map and encoded as pair features; an overall confidence score *c* ∈ [0,1]; component‐level focus scores; and auxiliary meta‐features (temperature alignment, hotspot density). We manually reviewed and validated all 800 JSON annotations and confirmed that they contain no direct selectivity information: no experimental *ee* values, no approximate numerical values that could hint at the outcomes, and no statements that reveal the substrate ranking. A representative annotation is provided in Table S2, and a word‐frequency analysis of all 800 entries is shown in Figure S1; both are consistent with this finding.

To verify that these two choices are individually necessary, we toggled the two factors independently across all four prompt conditions (Figure 3E), and scored each output on three metrics for which higher is better: Structured Encodability (convertibility into the pair‐feature representation), Anti‐Ambiguity (avoidance of vague positional references), and the composite ERS. JSON + mapped was strongest on all three (ERS 0.77, Structured Encodability 0.86, Anti‐Ambiguity 0.73). Schema constraint was the dominant factor: on its own (JSON + unmapped) it outperformed atom mapping on its own (Free‐form + mapped) in both ERS (0.44 vs 0.35) and Anti‐Ambiguity (0.42 vs 0.22); the unconstrained condition was worst (ERS 0.09). The two factors were also synergistic, with the combined condition exceeding the level expected from their independent gains on every metric, most strikingly for Anti‐Ambiguity (0.73 observed vs 0.48 under additivity). Beyond these design‐level checks, we assessed annotation quality directly: expert verification on a held‐out set of 50 reactions gave 92% atom‐level agreement, and the exact‐covered versus propagated‐covered provenance distinction was retained during training to modulate semantic authority (Supporting Information).

### 3.3 Confidence‐Gated Semantic Fusion

Not all source publications provide atom‐resolved mechanistic discussion. The semantic branch must therefore automatically down‐weight its contribution when evidence is sparse, and supply local corrections only when the literature support is strong. We implement this through a confidence gat_e_:

(2) $$g=\sigma \left(w_{g}\cdot m+b_{g}\right)\cdot \alpha$$

where *σ* is the logistic sigmoid, *w*_*g*_ and *b*_*g*_ are learnable weights and bias, ***m*** is a meta‐feature vector composed of the overall confidence, temperature alignment, hotspot density, and component‐level focus scores, and *α* is a global semantic scale factor. The final prediction takes the form:

(3) $$\begin{array}{ccc} h_{fused} & = & h_{phys}+g⊙f_{\mathrm{sem}}\left(h_{sem}\right)+g_{c}⊙\left(\gamma ⊙h_{phys}+\beta \right),\;\;\hat{y}\\ & & =head\left(h_{fused}\right) \end{array}$$

where *h*_*phys*_ is the structure‐based representation, *h*_*sem*_ is the encoding of the JSON annotation, *f*_*sem*_ is the sematic residual network, *g*⊙*f*_sem_(***h*** *_sem_*) is the gated semantic residual and the last term applies a Feature‐wise Linear Modulation (FiLM) [55] conditioning of ***h_phys_*** with conditioning gate *g_c_* (parameters in Table S3). When *g*, *g_c_* → 0, the model reduces to STEREO. *γ* and *β* are the FiLM scale and shift vectors. Unreported catalyst‐substrate combinations receive no reaction‐specific annotation and enter the semantic branch under a low‐reliability default prior (≈ 0.018). Because the semantic features enter only after graph encoding is complete, the E(3)‐equivariance of the structure‐based backbone is preserved throughout message passing; the scalar *ee*‐magnitude readout is correspondingly reflection‐invariant, matching the symmetry of the prediction target (Section S1.2). The prediction head receives only the gated, structure‐based encoding of the semantic features, not the features themselves, so the LLM branch cannot override the structural account of selectivity but can sharpen it where the literature provides atom‐resolved evidence.

## 4 Conclusion

In summary, we contribute both a dataset and a model for asymmetric C–H borylation. We curated the first unified dataset for this reaction class (to our knowledge), which comprises 800 enantioselective Ir‐catalyzed reactions over 174 catalysts and 606 components, each paired with an atom‐resolved, LLM‐extracted mechanistic annotation, and release the data, annotations, and code as an open resource. On this basis, STEREO‐Lit encodes catalyst, substrate, and product as distinct yet interacting 3D graphs with temperature‐aware kinetics and literature‐derived semantic priors, reaching *R* ^2^ = 0.749 and MAE = 5.19% *ee* on scaffold‐based held‐out reactions. Perturbation experiments attribute these gains to genuine, site‐specific mechanistic information rather than added capacity, and screening of ∼100 000 catalyst–substrate combinations nominated catalyst‐replacement candidates predicted to raise *ee*, three supported by transition‐state DFT. More broadly, we hypothesize that, because the framework requires only atom‐mapped structures and a documented mechanism, it may transfer to other asymmetric and catalytic reactions and extend to a more general structure–property model for small‐data tasks where the mechanism is documented in the literature but absent from structured datasets. Validating this transferability across reaction classes beyond Ir‐catalyzed C–H borylation is an important direction for future work.

## Author Contributions

**Zhe Wang**: methodology, software, conceptualization. **Baohua Zhang**: visualization. **Yi Isaac Yang**: resources. **Yuan Yao**: resources. **Yinwu Li**: data curation, writing – original draft, conceptualization.

## Conflicts of Interest

The authors declare no conflicts of interest.

## Supporting information




**Supporting File 1**: advs78094‐sup‐0001‐SuppMat.zip.

**Supporting File 2**: advs78094‐sup‐0002‐SuppMat.pdf.

## Data Availability

The data and code that support the findings of this study are openly available in a public GitHub repository at https://github.com/nickspark/STEREO, and the full extrapolation dataset is deposited in Zenodo at https://doi.org/10.5281/zenodo.22658981.
