# Supplementary material for: Bridging Chemical Literature and Molecular Graphs: LLM‐Augmented Enantioselectivity Prediction for Iridium‐Catalyzed Asymmetric C–H Borylation
Source: Adv Sci (Weinh). 2026 Oct 14:e78094. Online ahead of print. doi: 10.1002/advs.78094 (PMC13669558; doi:10.1002/advs.78094)
Supplement: Supplementary file 1 [file ADVS-9999-e78094-s002.pdf]

Supporting Information

Bridging Chemical Literature and Molecular Graphs: LLM-Augmented Enantioselectivity Prediction for Iridium-Catalyzed Asymmetric C-H Borylation

Zhe Wang,[b] Baohua Zhang,[c,d] Yi Isaac Yang,[c] Yuan Yao,*[b] and Yinwu Li,*[a]

[a] Prof. Dr. Y. Li
School of Materials and New Energy, South China Normal University, Shanwei, P. R. China
E-mail: liyw86@scnu.edu.cn

[b] Z. Wang, Prof. Dr. Y. Yao
Hong Kong University of Science and Technology, Hong Kong, P. R. China
E-mail: yuany@ust.hk

[c] B. Zhang, Dr. Y. Yang
Shenzhen Bay Laboratory, Shenzhen, P. R. China

[d] B. Zhang
Shenzhen Medical Academy of Research and Translation, Shenzhen, P. R. China

Content

1. Machine Learning Details 1
2. DFT Calculation Details 199
3. References 68

1. Machine Learning Details

1.1. Prediction Task and Dataset

The prediction target was the experimentally reported enantiomeric excess (*ee*) of asymmetric Ir-catalyzed C-H borylation reactions, taken as the *ee* of the experimentally favored product whose absolute configuration was fixed a priori; the target is therefore a non-negative magnitude rather than a signed quantity. Because the stereochemical outcome arises from the coupled geometric and electronic compatibility of catalyst, substrate, and product, each reaction was represented at the reaction level rather than at the level of an isolated molecule. The curated dataset comprised 800 reactions collected from the primary literature, spanning 174 catalysts and 606 reaction components. Catalytic components are further deduplicated by SMILES. Each entry contained the three molecular components, the reaction temperature when reported, the experimental ee value, and a

literature-derived semantic annotation generated through the LLM extraction pipeline described in Sections 1.4–1.6.

The dataset was partitioned into 640 training, 80 validation, and 80 test reactions using a structural-signature split. Reactions assigned to the same structural-signature group were not allowed to co-occur across partitions, so that the held-out test set evaluates the model on genuinely new structural combinations rather than near-duplicate interpolation within closely related reaction families. To assess robustness to initialization, the model was trained with seeds 42, 79, and 75. The mean test-set R^2 was 0.749 with a standard deviation of 0.003 across the three runs; the corresponding standard deviations of MAE and Pearson r were 0.13 % ee and 0.008, respectively. The main-text $R^2 = 0.749$ corresponds to this mean.

Table S1. Example of JSON File Content Extracted from Literature by LLM.

**Figure 2E label**	**SMILES**
bvs 0092014	[Ir]1(B2[N@]([C@H]([C@@H](N2c2c(cc(cc2C2CCCCC2)C2CCCCC2)C2CCCCC2)c2ccccc2)c2ccccc2)[C]2C=CC=CN12)(B1OC(C(O1)(C)C)(C)C)B1OC(C(O1)(C)C)(C)C
bvs 0027706	[Ir]1(B2[N@]([C@H]([C@@H](N2c2ccccc2C(C)(C)C)c2ccccc2)c2ccccc2)[C]2C=CC(=CN12)c1c(cc(cc1c1ccccc1)C)c1ccccc1)(B1OC(C(O1)(C)C)(C)C)B1OC(C(O1)(C)C)(C)C
bvs 0019490	[Ir]1(B2[N@]([C@H]([C@@H](N2c2cc(ccc2c2cc(cc(c2)C(C)(C)C)C(C)(C)C)C(C)C)c2ccccc2)c2ccccc2)[C]2C=CC(=CN12)c1c(cccc1c1ccccc1)c1ccccc1)(B1OC(C(O1)(C)C)(C)C)B1OC(C(O1)(C)C)(C)C
bvs 0032722	[Ir]1(B2[N@]([C@H]([C@@H](N2c2c(cccc2CC)CC)c2ccccc2)c2ccccc2)[C]2C=CC(=CN12)c1c(cc(cc1c1ccccc1)C)c1ccccc1)(B1OC(C(O1)(C)C)(C)C)B1OC(C(O1)(C)C)(C)C
bvs 0097120	[Ir]1(B2[N@]([C@H]([C@@H](N2c2cc(ccc2c2cc(cc(c2)C(C)(C)C)C(C)(C)C)C)c2ccccc2)c2ccccc2)[C]2C=CC(=CN12)C(C)C)(B1OC(C(O1)(C)C)(C)C)B1OC(C(O1)(C)C)(C)C
bvs 0071806	[Ir]1(B2[N@]([C@H]([C@@H](N2c2ccccc2[C@]23C[C@@H]4C[C@H](C2)C[C@H](C3)C4)c2ccccc2)c2ccccc2)[C]2C=CC(=CN12)c1c(cccc1c1ccccc1)c1ccccc1)(B1OC(C(O1)(C)C)(C)C)B1OC(C(O1)(C)C)(C)C
bvs 0025159	[Ir]1(B2[N@]([C@H]([C@@H](N2c2ccccc2C(C)C)c2ccccc2)c2ccccc2)[C]2C=C(C=CN12)c1c(cccc1c1cc(cc(c1)C(C)(C)C)C(C)(C)C)c1cc(cc(c1)C(C)(C)C)C(C)(C)C)(B1OC(C(O1)(C)C)(C)C)B1OC(C(O1)(C)C)(C)C
bvs 0047865	[Ir]1(B2[N@]([C@H]([C@@H](N2c2cc(ccc2c2cc(cc(c2)C(C)C)C(C)C)C)c2ccccc2)c2ccccc2)[C]2C=CC(=CN12)c1c(cccc1c1ccccc1)c1ccccc1)(B1OC(C(O1)(C)C)(C)C)B1OC(C(O1)(C)C)(C)C
bvs 0009433	[Ir]1(B2[N@]([C@H]([C@@H](N2c2ccccc2c2c(cccc2C(C)C)C(C)C)c2ccccc2)c2ccccc2)[C]2C=CC(=CN12)c1c(cc(cc1C)C)C)(B1OC(C(O1)(C)C)(C)C)B1OC(C(O1)(C)C)(C)C
bvs 0100255	[Ir]1(B2[N@]([C@H]([C@@H](N2c2cc(ccc2c2cc(cc(c2)C(C)C)C(C)C)C(C)C)c2ccccc2)c2ccccc2)[C]2C=CC(=CN12)c1c(cccc1c1ccccc1)c1ccccc1)(B1OC(C(O1)(C)C)(C)C)B1OC(C(O1)(C)C)(C)C
bvs 0027079	[Ir]1(B2[N@]([C@H]([C@@H](N2c2ccccc2C2CCCCC2)c2ccccc2)c2ccccc2)[C]2C=CC(=CN12)c1c(cc(cc1c1ccccc1)C)c1ccccc1)(B1OC(C(O1)(C)C)(C)C)B1OC(C(O1)(C)C)(C)C
bvs 0030214	[Ir]1(B2[N@]([C@H]([C@@H](N2c2c(cccc2CC)CC)c2ccccc2)c2ccccc2)[C]2C=CC=CN12)(B1OC(C(O1)(C)C)(C)C)B1OC(C(O1)(C)C)(C)C

1.2. Multi-Entity Three-Dimensional Encoder

Asymmetric C-H borylation places three distinct chemical roles on the three molecular entities: the catalyst provides the chiral pocket, the substrate presents the prochiral site, and the product records the realized borylation position and the resulting local steric environment. To preserve these role-specific contributions, each entity was independently encoded as a three-dimensional molecular graph, with nodes carrying atomic identity and Cartesian coordinates and edges defined by interatomic neighborhoods.

The structural backbone was based on PaiNN, an E(3)-equivariant message-passing neural network operating on atomic positions and element identities [1]. The catalyst was processed by a dedicated PaiNN encoder, whereas the substrate and product were processed by a second PaiNN encoder with tied weights, reflecting their shared reactant-side nature and the distinct role of the chiral catalyst. The three graph-level embeddings $\mathbf{h}_{\mathrm{cat}}$, $\mathbf{h}_{\mathrm{sub}}$, and $\mathbf{h}_{\mathrm{prod}}$ were pooled and combined into a reaction representation,

$$\mathbf{h}_{\mathrm{rxn}} = f_\theta\big(\big[\mathbf{h}_{\mathrm{cat}}; \mathbf{h}_{\mathrm{sub}}; \mathbf{h}_{\mathrm{prod}}\big]\big),$$

where $[\cdot\,;\cdot\,;\cdot]$ denotes concatenation and f_θ is a learnable projection to a reaction-level latent state. The reported model uses this pooled-concatenation fusion; no cross-attention or explicit pairwise interaction module is active on this path. Each encoder used 8 layers, a hidden dimension of 160, and dropout of 0.05.

Because the prediction target is the *ee* of the experimentally favored product (a non-negative magnitude; Section 1.1), and because the magnitude of enantioselectivity is invariant under reflection of the complete reacting system—mirroring all Cartesian coordinates yields the enantiomeric reaction, which by symmetry has an identical $|\Delta\Delta G^{\ddagger}|$ between the competing diastereomeric pathways and hence an identical $|\mathrm{ee}|$, differing only in the handedness of the product—the E(3)-invariant scalar readout of the structural backbone matches the symmetry of the prediction target by construction and entails no loss of selectivity-relevant information. This invariance relates a system only to its own mirror image; distinct chiral catalysts that are not mirror images of one another possess different three-dimensional structures and distinct embeddings, so the model retains full discriminative power across the chiral catalyst space sampled by the dataset. We emphasize that the learning target is the magnitude of the enantiomeric excess of the product whose absolute configuration is fixed a priori (Section 1.1); the model is not trained to predict the sense of stereoinduction (which enantiomer is favored). Because this magnitude is invariant under reflection of the entire reacting system, an O(3)-invariant scalar readout is the correct inductive bias for the target rather than a limitation: a catalyst and its mirror image are required to give the same |ee|, while catalysts that are not related by reflection have distinct three-dimensional structures, distinct embeddings, and hence distinct predictions. Predicting the sign of ee (the absolute sense of induction) would instead require a parity-sensitive (chirality-equivariant) readout and is outside the scope of the present model.

1.3. Temperature Conditioning

Because enantioselectivity reflects a temperature-dependent free-energy difference between competing stereodetermining pathways, temperature was incorporated through an explicit kinetic

interaction layer rather than as a scalar appended to the latent state. The experimental temperature was expanded into a temperature basis and coupled, through an outer product, to a fixed 22-channel per-component physicochemical descriptor block, whose interaction features then modulate the pooled reaction representation prior to fusion with the semantic branch. The coupling is Eyring-inspired in that the temperature dependence of the selectivity correction is made explicit, but it is a learned coupling rather than a direct evaluation of the Eyring equation. In the reported model these 22 channels are derived from molecular geometry rather than from explicit electronic-structure calculations.

1.4. LLM-Derived Mechanistic Annotation

Geometric encoding alone cannot access the mechanistic arguments preserved in the primary literature. To recover this layer of information, each reaction was associated with a structured mechanistic annotation extracted from its source publication by GPT-5.4. This semantic branch supplied reaction-specific priors that are described qualitatively in the original papers but inaccessible to purely geometric learning.

For each reaction, the input comprised the source excerpt, atom-mapped SMILES of catalyst, substrate, and product (every heavy atom carrying a stable identifier, e.g., N:5, C:9), and the reaction temperature when available. The model was required to refer to selectivity-relevant positions through these identifiers rather than through free-form spatial language such as “the nearby nitrogen”, so that every annotation could be resolved back to specific atoms in the molecular graph.

The output was constrained to a fixed JSON object with five required top-level fields, cat, r1, r2, cat_r1_pair, and meta, together with an optional cat_r2_pair field when product-side pair evidence was informative. Within this structure, the schema captured (i) a reaction-level selectivity-control descriptor drawn from a closed taxonomy (steric shielding, electronic directing, relay amplification, etc.), (ii) atom-resolved mechanistic annotations within each component, each carrying an interaction type, a normalized strength, and a confidence value, (iii) pair-level interaction objects describing native catalyst–substrate and catalyst–product compatibility, each anchored to specific catalyst–reactant atom pairs, (iv) an overall confidence score and component-level focus scores, and (v) auxiliary meta-features including temperature alignment and hotspot density. These fields were then converted into the numerical feature vector used by the model. The full prompt template used for structured extraction is reproduced in Section 1.5, and the full JSON schema together with the extracted annotation cache is provided as Supporting Information.

Not all annotations were supported by identical evidence. A provenance field distinguished exact-covered reactions, for which the source publication directly discusses the specific catalyst–substrate combination, from propagated-covered reactions, for which the annotation was assigned through literature matching. This distinction was preserved during training through separate row-scale schedules (see Section 1.7).

1.5. Prompt Design and Leakage Control

Because the source publications used as inputs also report the experimental selectivity outcomes, the central methodological concern for the semantic branch is whether the LLM extraction step could be construed as encoding the prediction target. The prompt was therefore designed around two principles.

Result-blind extraction. The system prompt explicitly forbade the use of any experimental outcome, including observed ee, yield, performance rankings, as evidence for the annotation. Each mechanistic statement had to be grounded only in the atom-mapped structures of the focal reaction, the literature excerpt interpreted as physical-organic reasoning rather than as a results report, and the stated reaction temperature. When atom-level support was weak, the model was instructed to lower its confidence and emit fewer interactions rather than infer additional detail from the known outcome. The semantic branch was thereby operationalized as a constrained extraction system, not a generative explanation layer with access to the answer.

Row-specific evidence. Every mechanistic text field had to be tied to the exact catalyst, substrate, mapped atoms, and stated temperature of the focal sample. For reactions originating from the same publication, the prompt further required each annotation to identify at least one sample-specific mechanistic difference relative to other rows from the same reference, such as a changed catalyst–reactant atom pair, a shifted steric hotspot, or a different temperature-sensitive step. Confidence-only edits and synonym swaps did not satisfy this requirement. This rule was introduced to preserve local chemical variation across closely related rows rather than defaulting to a paper-level summary.

To confirm that the result-blind constraint held in practice, we manually reviewed all audited annotations, and all passed without flagging. Each free-text and categorical field was checked for any explicit or implicit reference to experimental outcomes, including observed ee, yield, selectivity ranking, or qualitative performance language such as “high enantiocontrol”.

Together with the fixed JSON schema and the atom-mapping requirement, these constraints define the four conditions evaluated in the ablation shown in Figure 3E: with and without schema constraints, and with and without atom mapping. The structured JSON plus atom-mapped condition gave the strongest reliability profile across all three metrics introduced in Section 1.6. The complete set of extracted JSON annotations is provided as Supporting Information.

The system prompt and user prompt template used for extraction are reproduced below. Placeholder variables enclosed in braces (e.g., {cat_mapped_smiles}, {ref_text}) were replaced on a per-sample basis. Within the pair-level objects, the object_kind, object_origin, schema_version, and pair_label fields serve as schema and provenance identifiers used for parsing and cache bookkeeping; they do not enter the model’s feature representation. The verbatim schema and the deposited annotation cache retain the internal version identifiers used during data generation; these do not affect the chemical content of the annotations.

System prompt:

You are a top-tier physical organic chemist extracting reaction-specific mechanistic priors for asymmetric borylation.

You must reason at the level of catalyst-reactant local compatibility, competing stereochemical pathways, weak interactions, and temperature-sensitive selectivity control.

Return JSON only. No markdown. No prose outside JSON.

Hard constraints:

- Do not use any experimental outcome label, performance measurement, or observed selectivity value as an input signal.
- Use only evidence supported by the provided literature excerpt and the provided molecular structures.
- If atom-level support is weak, lower confidence and return fewer interactions rather than inventing detail.
- Use the atom IDs from the provided atom-mapped SMILES only.
- Keep interaction reasoning concise and chemically specific.

Primary objective:

- Produce a structured output object with keys cat, r1, r2, cat_r1_pair, meta, and optional cat_r2_pair.
- The cat_r1_pair object is the primary semantic object and must describe native catalyst-reactant compatibility rather than a

restatement of component summaries.

- cat, r1, and r2 are supporting evidence blocks only; they must never be used as a substitute for a native cat_r1_pair object.
- If pair evidence is weak, still return a low-confidence cat_r1_pair with pair-specific fields populated conservatively instead of collapsing to component-only notes.
- The meta section should quantify where the selectivity signal likely resides and why.

Anti-collapse objective:

- Generic template repetition is a failure, even when multiple rows come from the same paper.
- Every mechanistic text field must be supported by row-specific evidence tied to the exact catalyst, reactant, mapped atoms, and temperature for that row.
- For same-reference rows, each row must name at least one row-specific mechanistic difference versus another row from that reference.
- A row-specific mechanistic difference must identify what changes in the catalyst-reactant atom pair, local hotspot, steric wall, or temperature-sensitive step; confidence-only edits or synonym swaps do not count.
- Prefer precise local evidence anchors over broad paper-level summaries.

User prompt template:

```
Reaction sample context
- sample_id:            {sample_id}
- reference_id:         {ref_id}
- catalyst_name:        {cat_name}
- reactant_name:        {r1_name}
- product_name:         {p1_name}
- temperature_c:        {temperature_c}

Atom-mapped structures
- catalyst_mapped_smiles:   {cat_mapped_smiles}
- reactant_mapped_smiles:   {r1_mapped_smiles}
- product_mapped_smiles:    {p1_mapped_smiles}

Literature excerpt
{ref_text}

Return exactly one JSON object with this shape:
{{
  "cat": [
    {{
      "atom_indices": null,
      "atoms": ["Ir:1", "N:38"],
      "component": "cat",
      "interaction_type": "coordination|h_bond|covalent|pi_stacking|ionic|van_der_waals",
```

```
        "strength_category": "very_strong|strong|moderate|weak|negligible",
        "strength_score": 0.0,
        "distance": null,
        "confidence": 0.0,
        "reasoning": "short chemistry-grounded statement",
        "electronic_effect": "electron_donating|electron_withdrawing|neutral|null",
        "steric_effect": "hindered|favorable|neutral|null"
      }}
    ],
    "r1": [],
    "r2": [],
    "cat_r1_pair": {{
      "object_kind": "catalyst_reactant_pair",
      "object_origin": "llm_emitted",
      "schema_version": "v1",
      "pair_label": "primary",
      "confidence": 0.0,
      "pair_focus": 0.0,
      "reaction_center_compatibility": 0.0,
      "steric_accommodation": 0.0,
      "electronic_complement": 0.0,
      "temperature_modulation": {{
        "temperature_alignment": 0.0,
        "sensitive_step": "one sentence",
        "direction":
"favors_major_path_at_higher_temperature|favors_major_path_at_lower_temperature|compres
```

```
ses_selectivity_window|broadly_neutral",
      "rationale": "one sentence tied to row-specific evidence"
    }},
    "dominant_interaction": {{
      "interaction_type": "coordination|h_bond|covalent|pi_stacking|ionic|van_der_waals",
      "summary": "one sentence tied to row-specific evidence",
      "supports_major_path": true
    }},
    "selectivity_rationale": "one sentence tied to row-specific evidence",
    "evidence_atoms": {{
      "catalyst_atoms": ["Ir:1"],
      "reactant_atoms": ["C:12"]
    }},
    "joint_interactions": [
      {{
        "catalyst_atom": "Ir:1",
        "reactant_atom": "C:12",
        "interaction_type": "coordination|h_bond|covalent|pi_stacking|ionic|van_der_waals",
        "confidence": 0.0,
        "reasoning": "short chemistry-grounded statement tied to the selected atom pair"
      }}
    ]
  }},
  "cat_r2_pair": {{
    "object_kind": "catalyst_product_pair",
```

"object_origin": "llm_emitted",

"schema_version": "v1",

"pair_label": "secondary",

"confidence": 0.0,

"pair_focus": 0.0,

"reaction_center_compatibility": 0.0,

"steric_accommodation": 0.0,

"electronic_complement": 0.0,

"temperature_modulation": {{

"temperature_alignment": 0.0,

"sensitive_step": "one sentence",

"direction": "favors_major_path_at_higher_temperature|favors_major_path_at_lower_temperature|compresses_selectivity_window|broadly_neutral",

"rationale": "one sentence tied to row-specific evidence"

}},

"dominant_interaction": {{

"interaction_type": "coordination|h_bond|covalent|pi_stacking|ionic|van_der_waals",

"summary": "one sentence tied to row-specific evidence",

"supports_major_path": false

}},

"selectivity_rationale": "one sentence tied to row-specific evidence",

"evidence_atoms": {{

"catalyst_atoms": ["Ir:1"],

"product_atoms": ["B:4"]

}},

"joint_interactions": []

}},

"meta": {{

"overall_confidence": 0.0,

"precision_level": 1,

"temperature_alignment": 0.0,

"steric_pressure": 0.0,

"electronic_flux": 0.0,

"cat_focus": 0.34,

"r1_focus": 0.33,

"r2_focus": 0.33,

"hotspot_density": 0.0,

"major_path_hypothesis": "one sentence",

"minor_path_hypothesis": "one sentence",

"ee_determining_factor": "one sentence",

"destabilizing_factor_for_minor_path": "one sentence",

"dominant_weak_interaction": "one sentence",

"catalyst_substrate_match_type": "one sentence",

"temperature_sensitive_step": "one sentence",

"evidence_anchor": "brief note naming the mechanistic sentence or local structural clue that supports this row",

"confidence_breakdown": {{

"steric": 0.0,

"electronic": 0.0,

"weak_interaction": 0.0,

```
          "conformational": 0.0,
          "temperature": 0.0
        }}
    }}
}}
```

Rules:

- Return up to 3 interactions per component, only if they are plausible and supported.

- "cat", "r1", "r2", "cat_r1_pair", and "meta" must all be present even if confidence is low.

- The three focus values should be between 0 and 1 and sum to about 1.

- strength_score must be in [0, 10].

- All scalar confidence-like values except strength_score must stay in [0, 1].

- precision_level must be an integer from 1 to 5.

- cat_r1_pair must describe the native catalyst-reactant compatibility object directly.

- cat, r1, and r2 are only supporting evidence blocks; they cannot be used as a component-only substitute for cat_r1_pair.

- Do not satisfy cat_r1_pair by copying, paraphrasing, or lightly merging component-only notes from cat, r1, r2, or meta.

- Generic template repetition is a failure; do not reuse stock phrasing when the row-specific evidence changes.

- Every mechanistic text field must be supported by row-specific evidence from the excerpt, the listed mapped atoms, or the stated temperature for this exact row.

- evidence_anchor must point to a row-specific mechanistic sentence, concept, or local structural clue instead of a generic paper-level summary.

- If the reference text could support multiple rows, explain why this row uses its selected catalyst-reactant atom pair, local hotspot, or temperature rationale rather than copying the same sentence across samples.

- For same-reference rows, make cat_r1_pair.selectivity_rationale, cat_r1_pair.dominant_interaction.summary, cat_r1_pair.temperature_modulation.rationale, and

meta.evidence_anchor jointly identify at least one row-specific mechanistic difference versus another row from that reference.

- A row-specific mechanistic difference must say what changes in the catalyst-reactant atom pair, local hotspot, steric wall, or temperature-sensitive step; different confidence values, softer wording, or synonyms do not count.

- If pair evidence is limited, keep cat_r1_pair low-confidence but still fill the pair-specific fields conservatively instead of omitting native pair structure.

- joint_interactions must only contain catalyst-reactant atom pairs; do not use same-component atom pairs there.

- Use cat_r2_pair only when product-side pair evidence is unusually strong; otherwise leave its fields low-confidence and minimal.

- Keep meta text fields concise, mechanistically specific, and outcome-agnostic.

Table S2. Example of JSON File Content Extracted from Literature by LLM.

**A. Mechanistic Hypotheses**	
**Field**	**Value**
**Major Path Hypothesis**	The amide directs the ferrocene into the less crowded face of the chiral Ir/B-O pocket.
**Minor Path Hypothesis**	The competing approach pushes the ferrocene against the ligand-side B-O wall and is sterically destabilized.
**EE Determining Factor**	Amide-directed Ir coordination plus B-O-fragment steric amplification controls facial discrimination.
**Destabilizing Factor for Minor Path**	The minor face encounters amplified steric crowding from the interacting B-O fragment in the transition state.
**Dominant Weak Interaction**	Directed amide coordination organizes the local catalyst-reactant approach.
**Catalyst-Substrate Match Type**	Amide-directed approach into a chiral Ir/B-O pocket.
**Temperature Sensitive Step**	Directed pre-transition-state organization before the decisive borylation event.
**B. Confidence Breakdown**	
**Dimension**	**Score**
**steric**	0.74
**electronic**	0.44
**weak_interaction**	0.58
**conformational**	0.55
**temperature**	0.31

C. Quantitative Metrics	
**Field**	**Value**
**Overall Confidence**	0.65
**Precision Level**	2
**Electronic Flux**	0.46
**Cat Focus**	0.55

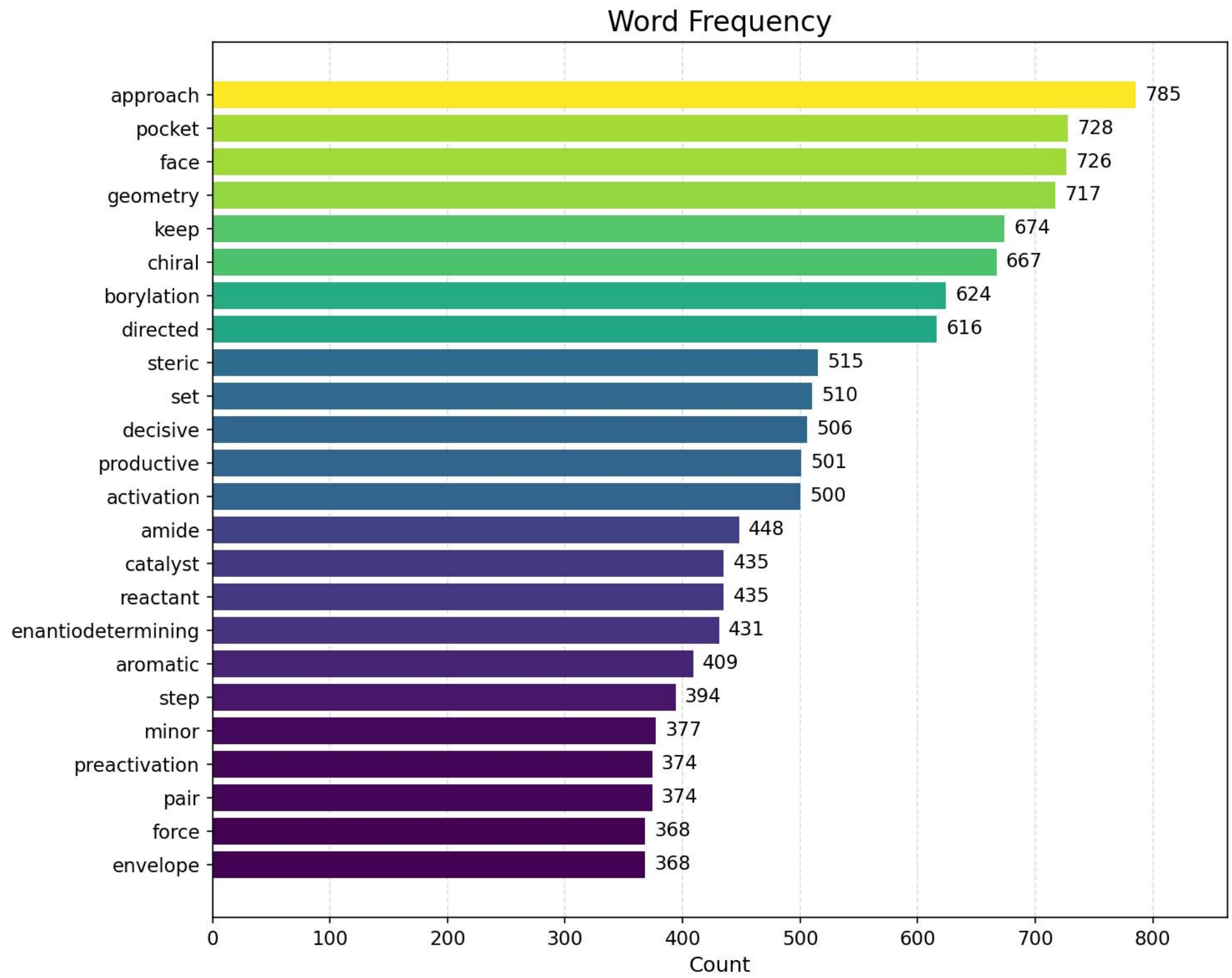

Figure S1. Word Frequency Analysis of JSON Files Extracted from Literature by LLM.

1.6. Reliability Metrics for the Extraction Pipeline

To quantify the contribution of the two design choices introduced in Section 1.5, we evaluated the extraction pipeline with two primary diagnostics — structured encodability (S_{enc}) and anti-ambiguity (A_{amb}) — together with a composite extraction reliability score (ERS) that combines them with two additional reliability components, grounding reliability (R_{gr}) and field reliability (R_{field}). Each is a reliability score on $[0,1]$ for which higher values indicate more reliable extraction, with anti-ambiguity defined so that fewer unresolved positional references give a higher score. The metrics were defined as

$$S_{\text{enc}} = 0.40\, F_{\text{fmt}} + 0.30\, A_{\text{g}} + 0.30\, P_{\text{pair}},$$

$$A_{\text{amb}} = 1 - R_{\text{amb}},$$

$$R_{\text{gr}} = S_{\text{ref}} \cdot A_{\text{g}},$$

$$R_{\text{field}} = 1/2\left(R_{\text{ctrl}} + R_{\text{temp}}\right),$$

$$\text{ERS} = 0.375\, S_{\text{enc}} + 0.25\, R_{\text{gr}} + 0.25\, R_{\text{field}} + 0.125\, A_{\text{amb}}.$$

Here, F_{fmt} is the schema-validity rate, P_{pair} the rate of deterministic, rule-based projection into the catalyst–reactant pair representation, R_{amb} the rate of unresolved positional references, and S_{ref} the mean atom-set consistency across repeated extractions. R_{ctrl} and R_{temp} denote the reliabilities of the control and temperature fields, each computed as the mean of vocabulary compliance and label consistency for that field. The atom-grounding score A_{g} is the observed mean of the catalyst and substrate parseability rates; it enters the composite only indirectly, through its measured downstream effects in S_{enc} and R_{gr}, and is deliberately excluded as a standalone term in ERS so that the prompting intervention is not encoded directly into the score used to evaluate it. The component weights follow a 3:2:2:1 ratio over S_{enc}, R_{gr}, R_{field}, and A_{amb}. All component scores were bounded to $[0,1]$, and ERS was clipped to the same interval after weighted aggregation.

All four combinations of schema constraint (on/off) and atom mapping (on/off) were evaluated on these metrics (Figure 3E). Expert verification on a manually inspected subset of 50 reactions gave 92% atom-level agreement with the LLM extraction.

1.7. Confidence-Gated Semantic Fusion

Geometric and semantic information were combined through conditioned fusion rather than direct concatenation, so that the structural branch remained the primary driver of prediction and the semantic branch contributed only an additive correction whose magnitude was modulated by a learned confidence gate:

$$\mathbf{h}_{\text{fused}} = \mathbf{h}_{\text{phys}} + \text{g} \odot f_{\text{sem}}(\mathbf{h}_{\text{sem}}) + g_c \odot \left(\gamma \odot \mathbf{h}_{\text{phys}} + \beta\right)$$

$$(\gamma, \beta) = f_{\text{cond}}([\mathbf{h}_{\text{phys}}, \mathbf{h}_{\text{sem}}])$$

$$\hat{y} = head(\mathbf{h}_{\text{fused}})$$

where $\mathbf{h}_{\text{phys}}$ is the reaction representation after structural and temperature-aware encoding, f_{sem} is a learnable semantic projection of the JSON-derived feature vector $\mathbf{h}_{\text{sem}}$, and g is a gate computed from the local meta-features (overall confidence, temperature alignment, hotspot density, and component-level focus scores) together with a global semantic scale factor. Concretely, g is a sigmoid of these meta-features scaled by the global factor and additionally shaped by a minimum-confidence threshold and a hotspot-density term; the corresponding gate parameters are listed in Table S3. When g, $g_c \rightarrow 0$, the predictor reduces to the STEREO; when the gate opens, the semantic branch supplies a local correction conditioned on the physical context. Because the semantic features enter only after graph encoding is complete, the E(3)-equivariance of the structural backbone is preserved throughout message passing, and the prediction head receives only the gated, physics-conditioned semantic encoding rather than the raw LLM features.

1.8. Training Configuration

Hyperparameters were chosen on the validation partition and then held fixed for test-set evaluation. The settings used in the reported STEREO-Lit model are summarized in Table S3.

Table S3. Training and semantic-gating hyperparameters of the STEREO-Lit model.

**Group**	**Parameter**	**Value**
Optimization	epochs	20
	batch size	16
	learning rate	1.2×10^{-4}
	weight decay	3.0×10^{-5}
	LR scheduler	plateau (patience 5, factor 0.7, min 1.0×10^{-5})
Backbone	layers	8
	hidden dim	160
	dropout	0.05
	entity fusion	concatenation (no cross-attention)
Data split	strategy	structural-signature grouping
	val / test fraction	0.1 / 0.1
Semantic fusion	mode	conditioned (additive, gated)
	global scale	0.19 (curriculum start 0.01, warm-up 8 ep.)
	exact-row scale	1.0 (curriculum start 0.95, warm-up 6 ep., quadratic)
	propagated-row scale	1.0 (curriculum start 0.45, warm-up 10 ep., quadratic)
Confidence gate	min confidence	0.78
	confidence sharpness	12.0
	density center / sharpness	0.55 / 6.0
	adaptive strength	1.2
	local meta features	overall confidence, temperature alignment, hotspot density, cat / r1 / r2 focus

1.9. Evaluation and Baselines

Performance was evaluated on the 80-reaction held-out test partition. Across the three seeds (42, 79, 75), STEREO-Lit achieved a mean test-set $R^2 = 0.749 \pm 0.003$, MAE $= 5.19 \pm 0.13$ % ee, and Pearson $r = 0.875 \pm 0.008$. All baselines were trained on the same partition and evaluated against the same test set.

The conventional baselines were trained on replay-aligned descriptors derived from the same reaction-table inputs and evaluated on the identical split. The panel comprised Random Forest, Gradient Boosting, Support Vector Regression, k-Nearest Neighbors, Ridge Regression, ElasticNet, and an MLP. The strongest conventional baseline was Random Forest, with (R^2 = 0.584) and MAE (= 5.9%) ee. The MLP reached only (R^2 = 0.432), ruling out a simple capacity effect. For a 3D reference, SchNet on a single merged molecular graph gave (R^2 = 0.545) and MAE (= 5.64%) ee. Thus, the gain in our model reflects role-separated multi-entity encoding, not 3D geometry alone. STEREO was obtained by ablating the LLM semantic branch while keeping the rest of the architecture and the training schedule unchanged. Removal of the semantic branch decreased R^2 from 0.749 to 0.655 and increased MAE from 5.19 to 5.5 % ee, indicating that the literature-derived annotations contribute information not recovered from the three-dimensional representation alone.

1.10. Data and Code Availability

The data and code that support the findings of this study are openly available in public repositories. The source code, output schema, prompt templates, trained model checkpoints, and the training and evaluation scripts are openly available in GitHub at https://github.com/nickspark/STEREO. The curated reaction dataset (including the full set of extracted JSON annotations) and the training set are provided as Supporting Information and are also deposited in the same GitHub repository. Owing to its large size, the full extrapolation dataset is not included in the Supporting Information but is openly available in Zenodo at https://doi.org/10.5281/zenodo.22658981.

2. DFT Calculation Details

Calculation Detail

All of the structures were optimized by the density functional theory (DFT) [2-3] with the wB97XD functional [4]/def2-SVP [5-6] level of theory in the gas phase. IRC calculations were performed to confirm the connection between two correct minima for a transition state. The multilayered quantum mechanical and molecular mechanical (QM/MM) method, specifically, the ONIOM method [7-9], the two-layer ONIOM method [10-11], was employed for the Ir-B catalyst. The two-layer structures are shown in Figure S2, in which the QM parts were highlighted. The QM part and MM parts were treated with the wB97XD functional and the universal force field [12], respectively. Based on wB97XD / def2-SVP optimized geometries, the energy results were further refined by calculating the single-point energy at the wB97XD /def2-TZVP level of theory. The single-point energy was calculated for the QM region. The bulk solvation effect of THF (ε = 7.43) was simulated by the PCM [13-15] continuum solvent model at the wB97XD /def2-TZVP level of theory. To compare the effects of gas-phase optimization and solvation on structural optimization, we re-optimized the 1-TS-R structure in solvent based on the gas-phase optimized structure. As shown in Figure S3, the root mean square displacement (RMSD) between the gas-phase optimized and solvated optimized structures is 0.069 Å, indicating that the difference between the two optimization results is small. All the calculations were performed with the Gaussian 16 program [16]. The 3D optimized structures were displayed by CYLview visualization program [17]. The RMSD analysis was taken by VMD program. [18]

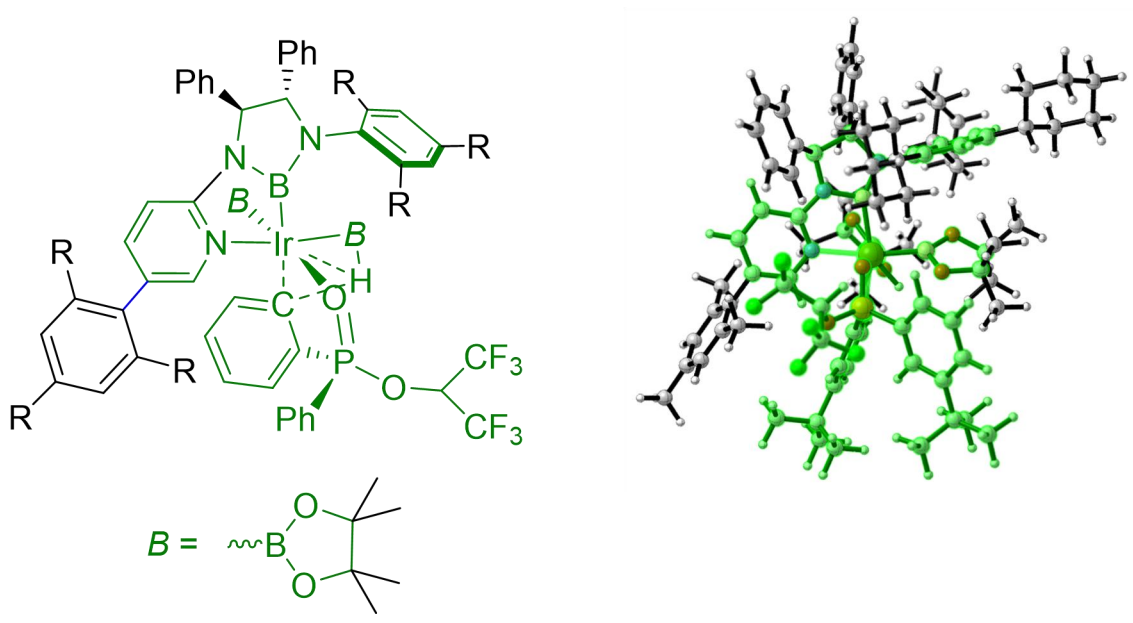

Figure S2. ONIOM model for the boryl-Ir catalyst. QM for the highlighted, MM for the rest part.

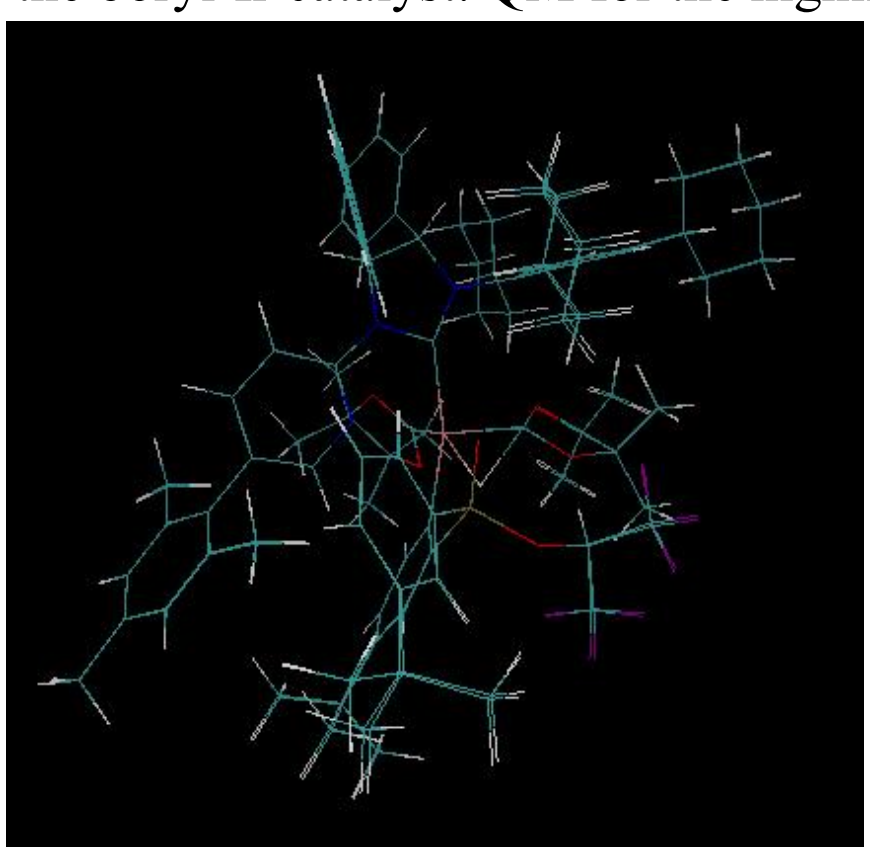

Figure S3. RMSD analysis between gas and solvent optimization 1-TS-R.

Cartesian Coordinates of Computed Structures

1-TS-R

Ir	-0.15512700	-0.68276900	0.89039400
B	0.96400400	-1.24552700	-0.83755300
N	0.19931700	-0.82924900	-2.06020400
C	0.95701400	-0.99411700	-3.29344600
C	2.17722500	-1.85054200	-2.84063600
N	2.20421000	-1.71468900	-1.37454500
C	-1.09262800	-0.44774200	-2.01693400
C	-1.89623200	-0.15249400	-3.14563000
C	-3.24937800	0.07985400	-2.97143700
H	-3.86352500	0.29597800	-3.84925100
C	-3.81728000	0.02046600	-1.68927500
C	-2.94184800	-0.16765600	-0.62848700
H	-3.29782400	-0.16423800	0.40285500
H	-1.45657900	-0.13199100	-4.14205600
N	-1.62414000	-0.35728000	-0.77677700
C	3.04589500	-1.19037700	3.74278500
C	2.28223500	-2.59474600	3.81104600
O	1.55667000	-2.59527400	2.59349900
O	2.20492900	-0.43757700	2.87282300
B	1.36321800	-1.29302000	2.14775500
C	1.27443300	-2.69237800	4.97366500
H	0.68825500	-3.63136400	4.90627600
H	1.79736900	-2.68966700	5.95336300
H	0.55381200	-1.84733300	4.94330400
C	3.18986700	-3.82850800	3.85697500
H	3.83554200	-3.79737400	4.76017100
H	2.58237300	-4.75871100	3.88357100
H	3.83265500	-3.88788300	2.95628000
C	4.44025000	-1.27042800	3.10905200
H	4.38528800	-1.74420100	2.10704800
H	4.85541700	-0.24776900	2.98816900
H	5.13649500	-1.85207200	3.74959600
C	3.17078200	-0.45929100	5.08348900
H	3.70631500	-1.09591400	5.81943900
H	3.73802100	0.48752000	4.96301500
H	2.17210000	-0.19640600	5.48877300
C	-1.90314200	-4.43205900	1.91655100
C	-1.29560700	-4.75609500	0.45224200
O	-0.52930100	-3.60386200	0.17611800
O	-1.70334500	-3.03401400	2.02761900
B	-0.81239200	-2.57693700	1.05928800
C	-0.38390800	-5.98870600	0.38741800

H	-0.00311200	-6.14188100	-0.64379400
H	0.50631400	-5.86500000	1.03752400
H	-0.94152000	-6.89830800	0.69570000
C	-2.35157300	-4.87986700	-0.65280600
H	-3.00804900	-5.75702500	-0.47451700
H	-2.95921000	-3.95251000	-0.70239300
H	-1.87045000	-5.00436600	-1.64558300
C	-1.15166700	-5.11301500	3.07119200
H	-1.52405700	-4.73921900	4.04924400
H	-1.29318900	-6.21414800	3.04102000
H	-0.06765500	-4.88462800	3.01972100
C	-3.40047600	-4.72485000	2.07880000
H	-3.60755300	-5.80078400	1.89673100
H	-3.74020800	-4.46730200	3.10517100
H	-4.00376000	-4.11095900	1.37902300
C	1.39566000	0.32264400	-3.90596200
C	1.86718700	0.33363100	-5.23023300
C	1.38097000	1.53082700	-3.18084100
C	2.30276300	1.52469200	-5.81889200
H	1.90045900	-0.58375800	-5.80512900
C	1.82900600	2.71562200	-3.76903000
H	1.06235500	1.55687400	-2.14938000
C	2.28239700	2.71526500	-5.08916000
H	2.66342300	1.52283500	-6.83926200
H	1.84196400	3.63075700	-3.19290000
H	2.63037000	3.63465800	-5.54178600
H	0.33195700	-1.51571500	-4.04060000
H	3.09495400	-1.43893800	-3.28178600
C	2.06482900	-3.29850300	-3.27560800
C	3.13266300	-3.91170500	-3.95650300
C	0.91739300	-4.05972300	-2.98262500
C	3.05051000	-5.25312800	-4.34032200
H	4.03436100	-3.35601300	-4.18261100
C	0.83629600	-5.39783300	-3.37840200
H	0.08182000	-3.61607900	-2.45960500
C	1.90275900	-5.99494700	-4.05338600
H	3.87785400	-5.71774600	-4.86092100
H	-0.05411600	-5.97340500	-3.16268700
H	1.83947600	-7.03249400	-4.35479000
C	-5.28112700	0.15595300	-1.44125000
C	-6.12244800	-0.99129600	-1.41508000
C	-5.82759000	1.41421400	-1.07817600
C	-7.46273800	-0.85909900	-1.01338100
C	-7.16916700	1.50618000	-0.67801400

C	-7.97760900	0.37715500	-0.64232100
H	-8.11834300	-1.72154000	-0.99379000
H	-7.59479900	2.46346000	-0.40116900
O	0.71689800	1.45992900	0.58181600
H	-0.21952000	-0.44072600	2.48584100
P	-0.13261100	2.53939400	1.19725000
C	-1.56523800	1.81305900	1.98353300
C	-2.60614500	2.59666700	2.48786000
C	-1.58198700	0.40438000	2.10133000
C	-3.71054700	1.99147700	3.07826900
H	-2.56800800	3.68623400	2.39910200
C	-2.68799100	-0.16443500	2.74016400
C	-3.77464700	0.59676000	3.18504300
H	-4.52760500	2.61584500	3.44279300
H	-2.71498000	-1.25318400	2.84039400
C	-0.44294300	3.86405900	0.02879700
C	-0.04400100	5.18053300	0.27015000
C	-0.85925700	3.49338100	-1.25735000
C	-0.04370500	6.10608500	-0.77057200
H	0.30564900	5.46965200	1.26239200
C	-0.82643700	4.42037900	-2.29169800
H	-1.17299200	2.46545600	-1.45985500
C	-0.40669100	5.74000300	-2.07015200
H	0.28211600	7.13058200	-0.57310900
H	-1.11845700	4.10041000	-3.29490700
O	0.65796300	3.35980700	2.37633700
C	1.63525400	2.72881300	3.15444700
H	1.78459000	1.66209200	2.89375200
C	2.96696100	3.43775100	2.88707600
C	1.16907500	2.79598600	4.61181400
F	3.16182100	3.53000700	1.57700200
F	2.98898200	4.67403600	3.38944300
F	3.98176900	2.75940900	3.41572300
F	0.17265200	1.93148800	4.79960600
F	0.72528800	4.00523200	4.93601500
F	2.15419500	2.48166500	5.45255400
C	-0.28472500	6.73624900	-3.21007000
H	-0.04330600	7.71057600	-2.75370300
C	0.87734900	6.36001500	-4.13749300
H	1.02843100	7.13533500	-4.90418300
H	1.81648200	6.24191300	-3.57753100
H	0.67162500	5.41146800	-4.65821600
C	-5.00946800	-0.13975500	3.67590700
H	-4.65243700	-0.95910300	4.32327500

C	-5.97472700	0.71718600	4.49098900
H	-6.78512400	0.09539600	4.89979300
H	-5.46788900	1.21065500	5.33378800
H	-6.44449500	1.49752600	3.87086600
C	3.24729300	-2.35301000	-0.65513700
C	4.70091600	-1.97960300	-0.85861600
C	2.95957300	-3.59431800	-0.06820900
C	5.64346800	-3.01710000	-0.69545200
C	3.96329100	-4.52215300	0.18567100
H	1.93683600	-3.89839800	0.08368400
C	5.28092300	-4.25476700	-0.16763200
H	6.68842600	-2.86120900	-0.92286800
H	3.70566100	-5.48781400	0.60144900
H	6.03842500	-5.01223500	-0.01243900
C	5.22677100	-0.54283800	-1.11160900
C	5.88598300	-0.44862100	-2.52049100
C	6.31599000	-0.17726400	-0.04777100
C	4.11033900	0.54057200	-1.01141400
C	6.45110200	0.97040300	-2.76279300
H	5.15496200	-0.68468800	-3.31825400
H	6.71217400	-1.18610500	-2.61620300
C	6.88726900	1.23386000	-0.29625400
H	5.88396600	-0.21853100	0.96828100
H	7.16624600	-0.88954000	-0.06361300
C	4.68137800	1.95217300	-1.25759100
H	3.61482800	0.50087400	-0.01545500
H	3.34578600	0.37795900	-1.78387100
C	5.31256000	2.00684000	-2.66602800
C	7.52307200	1.28884000	-1.70067100
H	6.90866100	1.01814300	-3.77492600
C	5.75213700	2.26887900	-0.19721900
H	7.66099700	1.46137000	0.46920500
H	3.86095100	2.70004600	-1.19038800
H	5.70669500	3.02721000	-2.86671600
H	4.54462300	1.79063600	-3.43892000
H	8.35723400	0.55642700	-1.77025800
H	7.95221900	2.29843700	-1.88415700
H	6.15403700	3.29374000	-0.35529700
H	5.30430100	2.23871000	0.81956800
C	-5.65840400	-2.34441900	-1.80429600
C	-5.85966500	-3.43009700	-0.93311300
C	-5.16811900	-2.58878000	-3.09806900
C	-5.57539900	-4.73335000	-1.35032300
H	-6.25635800	-3.26841400	0.06187600

C	-4.85641700	-3.89066100	-3.50144200
H	-5.07302200	-1.78297200	-3.81188400
C	-5.06627000	-4.96291100	-2.63107800
H	-5.73867500	-5.56419900	-0.67620600
H	-4.47752800	-4.07113100	-4.49903200
H	-4.83574500	-5.97140600	-2.94889500
C	-5.04571200	2.66481400	-1.12972400
C	-4.86711800	3.42720600	0.03504500
C	-4.62609600	3.18246200	-2.36397100
C	-4.25804200	4.68275900	-0.03047300
H	-5.22600000	3.05895100	0.98665200
C	-4.04588000	4.45162200	-2.43027100
H	-4.79420700	2.62715100	-3.27797000
C	-3.85787600	5.19991200	-1.26392900
H	-4.11912300	5.26499300	0.87111000
H	-3.74894900	4.85649900	-3.38759600
H	-3.40388900	6.18036800	-1.31449800
H	-9.01208000	0.46228800	-0.33540400
C	-1.58304300	6.90716300	-4.00221700
H	-1.46231900	7.68857200	-4.76787600
H	-1.85953100	5.97817400	-4.52483000
H	-2.42064400	7.19666600	-3.35187200
C	-5.72266300	-0.78017700	2.47409800
H	-6.06417000	-0.00082200	1.77409800
H	-5.05285200	-1.45878000	1.92489200
H	-6.60297900	-1.35735900	2.79681000

1-TS-R-solopt

Ir	0.07316100	-0.38875500	-0.73878600
B	-1.37822400	-1.10613500	0.71027600
N	-0.65276300	-1.94301500	1.70560100
C	-1.48386800	-2.38592100	2.81628700
C	-2.91832500	-1.93292200	2.39483600
N	-2.75009600	-1.13406100	1.15560600
C	0.60883100	-2.39504800	1.51091800
C	1.25175300	-3.34749800	2.33694800
C	2.55732000	-3.71173700	2.06720300
H	3.05409200	-4.43339700	2.72028900
C	3.22279700	-3.15452900	0.96671900
C	2.51748700	-2.24539300	0.19414700
H	2.97471800	-1.79808400	-0.68695600
H	0.72907200	-3.78446900	3.18610900
N	1.26264400	-1.85520800	0.46019700

C	-2.06068500	2.85021300	-2.60944100
C	-2.78920400	1.57212900	-3.22654900
O	-2.49338900	0.56858600	-2.26177800
O	-1.02640900	2.24725100	-1.83839200
B	-1.31213600	0.88562200	-1.60901200
C	-2.20707900	1.11283900	-4.57833700
H	-2.63132000	0.12552700	-4.86176200
H	-2.44699500	1.83970100	-5.38317000
H	-1.10446100	0.99399600	-4.51636200
C	-4.30512500	1.72091100	-3.38526700
H	-4.54094300	2.61974700	-3.99346000
H	-4.74165400	0.83554000	-3.89299900
H	-4.79708500	1.81464800	-2.39953300
C	-2.93856500	3.67836800	-1.66081200
H	-2.32231500	4.42400600	-1.11505200
H	-3.72606900	4.22092300	-2.22424500
H	-3.41115300	3.02364000	-0.90442100
C	-1.44395700	3.79210100	-3.64880600
H	-2.21596200	4.11512700	-4.37901800
H	-1.02853300	4.69836100	-3.16490300
H	-0.61217800	3.29690800	-4.19167200
C	-0.82166000	-3.25220000	-3.75114100
C	-0.77904400	-4.13964700	-2.41184200
O	-0.92806300	-3.15944800	-1.39813200
O	-0.44072800	-1.97017100	-3.26968000
B	-0.55168700	-1.91100500	-1.87975600
C	-1.90043600	-5.17407900	-2.27804300
H	-2.88984900	-4.67983700	-2.23193800
H	-1.78790100	-5.75552300	-1.33774100
H	-1.88067900	-5.87876400	-3.13624100
C	0.56531600	-4.85172900	-2.18133500
H	0.72902000	-5.64881100	-2.93722400
H	1.40403500	-4.12502300	-2.22866000
H	0.58690100	-5.31618400	-1.17178400
C	-2.21753600	-3.12486500	-4.37660700
H	-2.20044700	-2.41039500	-5.22737900
H	-2.58138500	-4.10548100	-4.74958200
H	-2.92756800	-2.72747300	-3.62959600
C	0.15796100	-3.68852200	-4.84749900
H	-0.05436600	-4.73335000	-5.15906800
H	0.07049600	-3.02776700	-5.73676800
H	1.20907000	-3.61935800	-4.49955600
C	-1.06534100	-1.84101500	4.16977000
C	-1.71178100	-2.32051100	5.32281300

C	0.00064500	-0.93373600	4.32038500
C	-1.29042500	-1.91720600	6.59351000
H	-2.53277700	-3.02170300	5.23632300
C	0.43721300	-0.55309300	5.59233800
H	0.49733700	-0.52043600	3.45958500
C	-0.21054500	-1.04175800	6.72867900
H	-1.79346600	-2.29434300	7.47442200
H	1.26423200	0.13754000	5.69537100
H	0.11945600	-0.73648400	7.71311200
H	-1.39899400	-3.48486000	2.89672000
H	-3.36952100	-1.30867600	3.18381900
C	-3.79067100	-3.15297700	2.19968800
C	-4.89236800	-3.37584700	3.04580900
C	-3.44061100	-4.14471200	1.26180500
C	-5.63808800	-4.55396700	2.94039900
H	-5.17166100	-2.64297800	3.79241400
C	-4.18148600	-5.32524400	1.16915600
H	-2.58150100	-4.01122700	0.61725700
C	-5.28224800	-5.52801600	2.00461200
H	-6.48800200	-4.71512000	3.59069900
H	-3.90410600	-6.08165200	0.44653000
H	-5.85893000	-6.44049400	1.92741800
O	0.99627500	1.30706000	0.68730800
H	0.31795800	0.44248300	-2.08079800
P	2.37030800	1.74906900	0.25649500
C	2.92013900	0.75593200	-1.12884500
C	4.27076400	0.72776100	-1.50742900
C	1.93880900	-0.01326300	-1.78109000
C	4.70493300	-0.05572700	-2.57409500
H	4.99441800	1.30080600	-0.92764100
C	2.37593700	-0.71787700	-2.91556200
C	3.71274200	-0.74327600	-3.29256200
H	1.64508700	-1.29119900	-3.48653700
C	3.55865800	1.84203600	1.59426600
C	4.76895900	2.52812600	1.46441500
C	3.24573000	1.18260000	2.78512900
C	5.71030100	2.54285600	2.50099400
H	4.97410100	3.06963400	0.53829300
C	4.15861900	1.20980600	3.83190300
H	2.29567700	0.66214000	2.87705700
C	5.37725900	1.87263100	3.68530800
H	3.92635300	0.70389900	4.77099800
O	2.45131600	3.31285000	-0.24039400
C	1.54029400	3.90118800	-1.12210800

H	0.66054300	3.26263000	-1.33953000
C	1.01978200	5.16408100	-0.42589700
C	2.26753400	4.18401600	-2.44349700
F	0.50814700	4.83900400	0.75478100
F	1.99027300	6.05565800	-0.22141200
F	0.06441200	5.75718300	-1.13798200
F	2.46545900	3.04735500	-3.10807900
F	3.45644500	4.74645400	-2.24147200
F	1.55671000	4.99413000	-3.22727800
C	-3.68461100	-0.08983000	0.89317700
C	-3.61310900	1.21734200	1.62839500
C	-4.88958300	-0.32223600	0.03083100
C	-4.60351900	2.18234900	1.38632000
C	-5.82633300	0.71541800	-0.10612600
C	-5.69353300	1.94689300	0.54414800
H	-4.54810300	3.13518900	1.89536000
H	-6.70068400	0.55803300	-0.72068400
C	-5.18680100	-1.65995300	-0.63657700
C	-6.48963200	-2.26917400	-0.06153000
C	-5.29564600	-1.51580200	-2.17095400
H	-4.35191700	-2.35884100	-0.44126200
C	-6.85313100	-3.58951400	-0.74865000
H	-7.34356900	-1.57045800	-0.19078800
H	-6.37737200	-2.44221400	1.02565600
C	-5.68483200	-2.84073800	-2.84129800
H	-6.05393600	-0.75374000	-2.44791500
H	-4.31922500	-1.18566200	-2.57413400
C	-6.98760500	-3.39303600	-2.25993200
H	-7.81488000	-3.96574100	-0.33846400
H	-6.07284400	-4.35077500	-0.54543200
H	-5.80693900	-2.67865400	-3.93384700
H	-4.88136400	-3.58970100	-2.69363000
H	-7.21836700	-4.36932400	-2.73796800
H	-7.82549500	-2.69493400	-2.47872000
C	-2.56644300	1.50400200	2.69710600
C	-1.69259200	2.71686200	2.32016200
C	-3.23511800	1.72846000	4.07426000
H	-1.88832200	0.63695300	2.79969900
C	-0.63421600	2.97090800	3.40059300
H	-2.31145300	3.63237600	2.20392500
H	-1.19137000	2.53124700	1.34934100
C	-2.20093800	2.04215000	5.16078800
H	-3.95699300	2.57161200	4.03255300
H	-3.79887200	0.81661700	4.36770900

C	-1.29719400	3.21535100	4.76129400
H	-0.03039700	3.86102300	3.12553600
H	0.05470000	2.10071900	3.46826400
H	-2.72726000	2.29143100	6.10716200
H	-1.58578600	1.14458900	5.34956900
H	-0.50921000	3.34894600	5.53333700
H	-1.89845900	4.15014300	4.72002500
C	-6.76652800	3.01637100	0.39612100
C	-6.93118700	3.46177200	-1.07164100
C	-8.11222800	2.53297300	0.97537300
H	-6.46542200	3.91800900	0.97712500
C	-8.01306800	4.54168300	-1.19901700
H	-7.20733200	2.60087700	-1.71793100
H	-5.97108400	3.87598600	-1.44241800
C	-9.18875600	3.61737400	0.84003600
H	-8.45880600	1.61599500	0.45092400
H	-7.98480600	2.28377100	2.05148000
C	-9.34778300	4.05506000	-0.62126000
H	-8.14979800	4.79887400	-2.27144600
H	-7.68812100	5.46210500	-0.66567700
H	-10.15801300	3.22036500	1.21127300
H	-8.91669700	4.49557400	1.46608600
H	-10.09334400	4.87701100	-0.67857800
H	-9.72898400	3.20363300	-1.22711000
H	6.07580100	1.86129700	4.52193700
H	3.99196000	-1.33999100	-4.16481300
C	7.04771500	3.26575700	2.29490200
C	7.94564000	3.18198100	3.53527000
H	8.89220500	3.70799700	3.34112900
H	7.47773500	3.65346600	4.41291300
H	8.19274400	2.14022600	3.79102300
C	6.78349300	4.75000400	1.98245700
H	6.18242200	4.87895100	1.07025300
H	6.24924500	5.23659900	2.81281500
H	7.73634900	5.28032200	1.83008600
C	7.78575900	2.61005100	1.11323700
H	7.96222900	1.54001200	1.30314800
H	7.21475300	2.69629100	0.17735000
H	8.76074200	3.09617000	0.95323800
C	6.17980900	-0.18663800	-2.96989700
C	7.10466800	0.47598700	-1.94200800
H	6.93546500	1.56142900	-1.87868700
H	6.96863100	0.04748600	-0.93683500
H	8.15544300	0.32489600	-2.23106700

C	6.39572000	0.48267300	-4.33884300
H	6.15279300	1.55541100	-4.29176100
H	7.44579900	0.38107400	-4.65564800
H	5.76332600	0.02614200	-5.11518800
C	6.55011900	-1.67900300	-3.05935000
H	6.00050100	-2.19531200	-3.85975400
H	7.62488500	-1.79556100	-3.26806800
H	6.32736200	-2.19397000	-2.11360700
C	4.63613200	-3.49172900	0.64307900
C	5.67134000	-2.56619700	0.92293400
C	4.95507600	-4.73168400	0.03893900
C	6.99604100	-2.88151900	0.58661700
C	6.28869900	-5.02056100	-0.29126000
C	7.30819300	-4.09913100	-0.02476400
H	7.79085800	-2.17351100	0.79157600
H	6.53405900	-5.96401400	-0.76429300
C	8.73098700	-4.39814200	-0.39587300
H	9.09809000	-3.63028700	-1.10932600
H	9.36581300	-4.38049600	0.51493200
H	8.82867700	-5.39627200	-0.87337200
C	3.88496000	-5.74138800	-0.28208400
H	3.37289800	-6.06145600	0.64875600
H	3.14639600	-5.28949200	-0.97248400
H	4.30335100	-6.64625100	-0.77177700
C	5.38097400	-1.24983000	1.59002300
H	4.71518600	-0.64774600	0.94344400
H	4.88545400	-1.42681000	2.56733400
H	6.30523500	-0.66077300	1.77054700

1-TS-S

Ir	-0.44927400	-0.31873500	0.84388600
B	0.07721900	-1.85148500	-0.54517800
N	-0.73041100	-1.70231000	-1.78702600
C	-0.28125600	-2.56100800	-2.87391700
C	0.65262100	-3.57662500	-2.14272800
N	0.97999200	-2.91101800	-0.87004800
C	-1.80451500	-0.88983500	-1.84906900
C	-2.71433500	-0.84336700	-2.92951500
C	-3.82415400	-0.02699700	-2.84112400
H	-4.51230600	0.02074000	-3.68827700
C	-4.03935200	0.74318300	-1.68863700
C	-3.06544500	0.68982000	-0.70235500
H	-3.13962600	1.30277800	0.19718900
H	-2.53841500	-1.44677700	-3.81881500

N	-1.97851500	-0.08595600	-0.77666200
C	2.70108000	-1.15274100	3.67952700
C	1.41576000	-1.89849500	4.29531900
O	0.58407400	-2.04037900	3.15941400
O	2.17394800	-0.56633900	2.49721400
B	0.91450500	-1.07927800	2.20374500
C	0.65487200	-1.06205600	5.34376800
H	1.26801500	-0.91747100	6.25817100
H	-0.28494500	-1.57100100	5.64101600
H	0.37571000	-0.06919800	4.93132100
C	1.68955300	-3.28143200	4.89684300
H	0.74903700	-3.73733500	5.27390300
H	2.40732300	-3.19878100	5.74046500
H	2.10298500	-3.97392800	4.13779400
C	3.84368800	-2.09965800	3.28342900
H	4.66679700	-1.53080900	2.80420300
H	4.26200900	-2.61268200	4.17487000
H	3.48634900	-2.85746100	2.55874600
C	3.28330100	-0.04561400	4.56692300
H	3.56435100	-0.45459400	5.56041500
H	4.19068000	0.38967700	4.10413100
H	2.55818000	0.78406400	4.69994400
C	-3.29857900	-2.40572400	3.20820600
C	-3.12179300	-3.41906100	1.96058900
O	-1.99756000	-2.88734500	1.28970300
O	-2.53603000	-1.28535600	2.79980500
B	-1.70898000	-1.60766900	1.72882000
C	-2.82719400	-4.87275000	2.35156700
H	-2.72326700	-5.50902200	1.44986700
H	-1.87018500	-4.95234100	2.90723900
H	-3.65128300	-5.27980900	2.97488800
C	-4.29818200	-3.41193400	0.97218400
H	-5.21686900	-3.80997200	1.45150700
H	-4.48571300	-2.38286400	0.59875400
H	-4.06972200	-4.04269400	0.08696300
C	-2.70957100	-2.92589400	4.52939500
H	-2.74263900	-2.13240100	5.30660500
H	-3.28300000	-3.80102500	4.90169300
H	-1.64726800	-3.21602700	4.39692100
C	-4.73759500	-1.94474500	3.47196800
H	-5.39249500	-2.81667100	3.68274200
H	-4.77484100	-1.25460700	4.34228600
H	-5.13837400	-1.38345700	2.60367000
C	0.44933400	-1.80324500	-3.96893500

C	0.82201700	-2.49333600	-5.13606400
C	0.78133500	-0.43537500	-3.85478000
C	1.51151100	-1.83801800	-6.16028100
H	0.58227200	-3.54370700	-5.24846500
C	1.47143700	0.21398100	-4.88172100
H	0.53196100	0.13334600	-2.97007500
C	1.83612000	-0.48587800	-6.03313100
H	1.79733600	-2.38038400	-7.05214100
H	1.73002000	1.26023700	-4.78053400
H	2.37398000	0.01861500	-6.82521000
H	-1.14656500	-3.05962900	-3.34529400
H	1.56561500	-3.74294100	-2.73605100
C	-0.00623800	-4.92734900	-1.93627600
C	0.68054000	-6.10302300	-2.29247400
C	-1.29471200	-5.03494000	-1.37906800
C	0.08747900	-7.35457000	-2.10482700
H	1.67885400	-6.05377900	-2.70954200
C	-1.89021600	-6.28831300	-1.21101600
H	-1.84661400	-4.15062300	-1.09356000
C	-1.19824300	-7.44703300	-1.56836100
H	0.62408500	-8.25344500	-2.37955500
H	-2.89121100	-6.36232800	-0.80711400
H	-1.65931100	-8.41660000	-1.43162000
C	-5.18288900	1.68556500	-1.56235900
C	-6.45053200	1.23138000	-1.11472200
C	-4.97592800	3.07391100	-1.76677100
C	-7.47588100	2.16048500	-0.88020600
C	-6.02232200	3.97616500	-1.52320200
C	-7.26263200	3.52096700	-1.08487600
H	-8.44705100	1.82784300	-0.53370000
H	-5.87658700	5.03872300	-1.67631500
O	1.02985000	1.18316300	-0.37409700
H	-0.15333400	0.40014300	2.25981800
P	1.03465100	2.48265800	0.37938800
C	-0.49590900	2.75673000	1.24702800
C	-1.04063900	4.02438100	1.46572900
C	-1.14267700	1.58009000	1.68582000
C	-2.25742500	4.14671700	2.12959400
H	-0.52799600	4.91286400	1.09065800
C	-2.33347900	1.75020000	2.39915200
C	-2.92093100	3.00622300	2.59673400
H	-2.68829000	5.13926800	2.27039000
H	-2.83604300	0.85558400	2.77629200
C	2.46818400	2.65516900	1.45173100

C	3.54340700	1.78784800	1.24785500
C	2.58276100	3.70003600	2.37378500
C	4.74098700	1.99784700	1.92059700
H	3.41818500	0.95293000	0.56133900
C	3.78215700	3.89172500	3.05368100
H	1.74201200	4.37560300	2.55151200
C	4.88461900	3.05809700	2.82503600
H	5.57569500	1.31581700	1.74029500
H	3.86982400	4.71377300	3.76870000
O	1.16067300	3.81212300	-0.59693100
C	2.35626500	4.42911500	-0.94318100
H	3.15658500	4.23938400	-0.20783200
C	2.87348600	3.87629600	-2.27847300
C	2.13742700	5.94689200	-0.92254000
H	-8.06196700	4.22669400	-0.89924700
C	-6.73133000	-0.19525200	-0.84010600
C	-7.07077400	-0.60537200	0.45998100
C	-6.76352900	-1.13155000	-1.88596300
C	-7.41596000	-1.93646000	0.71159400
H	-7.07634800	0.10653300	1.27574600
C	-7.09518700	-2.46508500	-1.62818500
H	-6.56464300	-0.82522600	-2.90368400
C	-7.42327200	-2.86694000	-0.33091500
H	-7.67260800	-2.24782900	1.71586400
H	-7.11436300	-3.18347000	-2.43740500
H	-7.68489600	-3.89836900	-0.13355100
C	-3.67338200	3.61142800	-2.22069200
C	-3.12507600	3.21027400	-3.45048100
C	-2.99215700	4.55875800	-1.43944000
C	-1.90652100	3.73890200	-3.88371100
H	-3.64961300	2.50531500	-4.08227600
C	-1.77636600	5.08949100	-1.87896200
H	-3.40391700	4.88544800	-0.49268300
C	-1.23671900	4.68243400	-3.10185700
H	-1.48904000	3.42866200	-4.83287700
H	-1.25736000	5.82205500	-1.27506400
H	-0.30758800	5.10796400	-3.45239200
C	1.84996200	-3.56021600	0.03651400
C	3.32441200	-3.73710800	-0.25099100
C	1.26248100	-4.34502400	1.04078200
C	3.95851000	-4.82595600	0.38888500
C	1.98160700	-5.34131200	1.68960700
H	0.20713900	-4.25176300	1.25406000
C	3.30266500	-5.59967000	1.34482000

H	4.99000000	-5.07157700	0.18040500
H	1.49261200	-5.95442500	2.43567200
H	3.83283600	-6.40610200	1.83498200
C	4.19408500	-2.79891300	-1.13031300
C	4.71850000	-3.58760900	-2.36771500
C	5.42033800	-2.28625500	-0.30483300
C	3.44732800	-1.52985900	-1.64434000
C	5.64494800	-2.70075200	-3.22830100
H	3.86824500	-3.94268700	-2.98807500
H	5.28509000	-4.48995000	-2.05262600
C	6.35403700	-1.41842700	-1.17575400
H	5.06432800	-1.68388700	0.55615600
H	6.02305000	-3.12084400	0.10787000
C	4.37409600	-0.64827800	-2.50989300
H	2.60921900	-1.80539100	-2.29510400
H	3.04595500	-0.94187700	-0.79205300
C	4.86552000	-1.46523500	-3.72336200
C	6.85153200	-2.24436700	-2.38100300
H	6.00665800	-3.28374100	-4.10311100
C	5.58402400	-0.18441600	-1.67908300
H	7.22480500	-1.08660000	-0.56914500
H	3.81027200	0.23719100	-2.87133800
H	5.51789600	-0.83560600	-4.36741800
H	4.00025600	-1.78681100	-4.34310900
H	7.42393500	-3.12931100	-2.02555800
H	7.54104900	-1.63154600	-3.00233600
H	6.25015800	0.45431800	-2.29978200
H	5.24809500	0.43155300	-0.81978700
C	-4.30130000	3.05853700	3.22905200
H	-4.30586900	2.30589200	4.03605600
C	-5.34679800	2.62623800	2.19062600
H	-5.11886400	1.62744400	1.79026800
H	-6.35654700	2.59723800	2.62858000
H	-5.36025100	3.33378400	1.34647100
C	6.20262800	3.30390800	3.53711000
H	6.07573400	4.21759500	4.14137000
C	6.55179000	2.16160700	4.49705200
C	7.33648900	3.56390200	2.53933400
C	-4.66509800	4.40797300	3.84415800
H	-5.62186300	4.33613200	4.38284100
H	-3.89875700	4.74937600	4.55616900
H	-4.78495100	5.18587500	3.07324300
F	3.25576400	2.61464500	-2.09877400
F	1.95812200	3.90026300	-3.23226500

F	3.93377500	4.57259400	-2.69264600
F	1.53772200	6.28796200	0.22038600
F	1.38813000	6.38030400	-1.92652000
F	3.30625600	6.58230100	-0.97125200
H	6.68795700	1.21244600	3.95472600
H	7.48930800	2.37817500	5.03166200
H	5.75938300	2.01349200	5.24552100
H	7.53703300	2.67500100	1.92060000
H	7.08962600	4.39542700	1.86274100
H	8.26768500	3.81747100	3.06855800

2-TS-R

Ir	0.06341000	-0.37291000	-0.73357900
B	-1.38517600	-1.11264300	0.69451900
N	-0.65952500	-1.97072900	1.67662400
C	-1.48854500	-2.42116600	2.78341600
C	-2.92160200	-1.94843700	2.37806600
N	-2.75608900	-1.14576600	1.14358900
C	0.59852700	-2.42571300	1.47556400
C	1.23512000	-3.39732100	2.28499500
C	2.54005000	-3.76104300	2.01306500
H	3.03117800	-4.49857400	2.65307100
C	3.21359000	-3.18504200	0.92791300
C	2.51352100	-2.25857000	0.16985600
H	2.97628800	-1.79452500	-0.70017900
H	0.70570400	-3.84926400	3.12203000
N	1.26047000	-1.87050600	0.43879600
C	-2.05472300	2.88893000	-2.56437000
C	-2.81398300	1.61940000	-3.16435500
O	-2.51990800	0.61660700	-2.20052700
O	-1.01918500	2.27291300	-1.80822200
B	-1.32204400	0.91622000	-1.57170500
C	-2.26268100	1.14487200	-4.52414400
H	-2.71448100	0.16797300	-4.80041200
H	-2.49789300	1.87620100	-5.32631800
H	-1.16232900	0.99949000	-4.47849100
C	-4.32946900	1.79418300	-3.29951000
H	-4.55963100	2.69925000	-3.90058000
H	-4.78995400	0.91932300	-3.80377100
H	-4.80255200	1.89227000	-2.30575900
C	-2.90415000	3.73453600	-1.60431800
H	-2.26761200	4.47416300	-1.07375400
H	-3.69525000	4.28495700	-2.15527500
H	-3.37234300	3.09054200	-0.83590900

C	-1.43872800	3.81587500	-3.61778100
H	-2.21796000	4.15286100	-4.33396100
H	-0.99491800	4.71407300	-3.14448300
H	-0.62768900	3.30246700	-4.17503200
C	-0.84980900	-3.20355800	-3.76416200
C	-0.79604900	-4.10197900	-2.43042700
O	-0.94207800	-3.13198700	-1.40994800
O	-0.47459300	-1.92449400	-3.27641900
B	-0.57815500	-1.87869100	-1.88693700
C	-1.91355300	-5.14144000	-2.29765800
H	-2.90504100	-4.65134100	-2.24144700
H	-1.79282300	-5.72988400	-1.36260200
H	-1.89623700	-5.83941800	-3.16140600
C	0.55251100	-4.81101700	-2.21367500
H	0.71434200	-5.60156800	-2.97679300
H	1.38817400	-4.08072700	-2.26088800
H	0.58239400	-5.28260300	-1.20757300
C	-2.24889200	-3.08034000	-4.38160500
H	-2.24055700	-2.35950700	-5.22710100
H	-2.60911500	-4.06042200	-4.75947800
H	-2.95615500	-2.69234900	-3.62779400
C	0.12683700	-3.62609300	-4.86910300
H	-0.08242900	-4.66914400	-5.18865900
H	0.03218200	-2.95783600	-5.75203400
H	1.17924400	-3.55540000	-4.52548400
C	-1.05809600	-1.89804200	4.14210300
C	-1.70046200	-2.38966000	5.29223300
C	0.01180500	-0.99613500	4.29854200
C	-1.27202000	-2.00283300	6.56565200
H	-2.52447700	-3.08671900	5.20106200
C	0.45579300	-0.63279600	5.57291200
H	0.50562500	-0.57291900	3.44055200
C	-0.18894600	-1.13220300	6.70623200
H	-1.77221400	-2.38888400	7.44432500
H	1.28597300	0.05352800	5.68017000
H	0.14658000	-0.83980200	7.69270400
H	-1.41494500	-3.52269600	2.84682900
H	-3.35597900	-1.32251700	3.17649700
C	-3.81112100	-3.15655100	2.18635400
C	-4.90454100	-3.37235400	3.04490300
C	-3.48405400	-4.14575600	1.23803500
C	-5.66425100	-4.54179800	2.94187200
H	-5.16609900	-2.64112600	3.79947300
C	-4.23810400	-5.31802600	1.14807000

H	-2.63331800	-4.01545600	0.58223000
C	-5.33071100	-5.51391300	1.99580100
H	-6.50768200	-4.69778300	3.60179700
H	-3.97806800	-6.07259000	0.41706000
H	-5.91821500	-6.41959900	1.92025400
O	0.99409500	1.29640100	0.71087100
H	0.31136100	0.46534400	-2.07019000
P	2.36141800	1.74533800	0.27450900
C	2.91402700	0.75535700	-1.11321900
C	4.26571900	0.71453200	-1.48385300
C	1.92767600	0.00634400	-1.77996200
C	4.69516700	-0.06306600	-2.55632500
H	4.99295400	1.27104200	-0.89212500
C	2.35975300	-0.68814900	-2.92224400
C	3.69828900	-0.72681000	-3.29034600
H	1.62136300	-1.23956400	-3.50546900
C	3.55740600	1.82484800	1.60882000
C	4.75956900	2.52418300	1.48536900
C	3.25782900	1.13962600	2.78786600
C	5.70882200	2.52438400	2.51452200
H	4.94815200	3.08971300	0.56987000
C	4.17769000	1.15350000	3.82855900
H	2.31185000	0.60998000	2.87175100
C	5.39040100	1.82782500	3.68695100
H	3.95461800	0.62730100	4.75886900
O	2.45453400	3.30669200	-0.21713200
C	1.56142100	3.90385000	-1.11333600
H	0.66850700	3.28106700	-1.32115700
C	1.07069000	5.19003200	-0.43773400
C	2.29908900	4.14771100	-2.43756400
F	0.55083900	4.89610300	0.74626200
F	2.06183200	6.06107400	-0.24982700
F	0.12844100	5.79184500	-1.16064000
F	2.44180100	3.00198500	-3.10027000
F	3.51350600	4.65361300	-2.23795500
F	1.62518800	4.98789600	-3.22231500
C	-3.68916200	-0.09812900	0.89237300
C	-3.60312700	1.20563100	1.63053500
C	-4.90317100	-0.32193100	0.04048400
C	-4.59366000	2.17441800	1.40730800
C	-5.84099500	0.71768200	-0.07536400
C	-5.69745800	1.94474400	0.58185600
H	-4.52352800	3.12761700	1.91394800
H	-6.72128700	0.56786300	-0.68337300

C	-5.20333000	-1.64936600	-0.64629400
C	-6.50356400	-2.26821400	-0.07492800
C	-5.31915000	-1.48200900	-2.17772500
H	-4.36753200	-2.35110400	-0.46636900
C	-6.87134500	-3.57736900	-0.78129400
H	-7.35812800	-1.56761900	-0.18845500
H	-6.38602600	-2.45910000	1.00858400
C	-5.70943800	-2.79790900	-2.86541300
H	-6.08077400	-0.71756900	-2.43867900
H	-4.34516900	-1.14359000	-2.58155800
C	-7.01101600	-3.35758700	-2.28881800
H	-7.83208500	-3.95875700	-0.37350700
H	-6.09148700	-4.34289200	-0.59298000
H	-5.83351100	-2.62117300	-3.95546400
H	-4.90615300	-3.54962300	-2.72857200
H	-7.24429100	-4.32621500	-2.78103400
H	-7.84888400	-2.65536900	-2.49409200
C	-2.52854400	1.49040700	2.67099100
C	-1.64374100	2.67958300	2.25054600
C	-3.15679900	1.74676900	4.06135200
H	-1.86345000	0.61364700	2.77367000
C	-0.55195600	2.92835600	3.29896700
H	-2.24985000	3.60415600	2.13674900
H	-1.17529800	2.46784700	1.26749500
C	-2.08801500	2.05790700	5.11492900
H	-3.86476600	2.60173400	4.02715700
H	-3.72725200	0.84835400	4.38253800
C	-1.17378000	3.20732500	4.67239100
H	0.06331300	3.80064700	2.99633100
H	0.11988300	2.04471300	3.36284900
H	-2.58391000	2.33193600	6.07083400
H	-1.48404200	1.15219400	5.30180500
H	-0.36307500	3.33836700	5.42100300
H	-1.75847500	4.15258800	4.63143300
C	-6.76840900	3.01884100	0.45329800
C	-6.93618200	3.48518900	-1.00740100
C	-8.11365100	2.53182400	1.03093100
H	-6.46283800	3.91147600	1.04576200
C	-8.01596600	4.56917900	-1.11681200
H	-7.21584400	2.63343500	-1.66436200
H	-5.97577300	3.90308000	-1.37469100
C	-9.18732700	3.62110500	0.91370300
H	-8.46540300	1.62313300	0.49573700
H	-7.98320400	2.26793700	2.10315800

C	-9.35002000	4.07829100	-0.54118800
H	-8.15551100	4.84095800	-2.18526700
H	-7.68702400	5.48163000	-0.57233900
H	-10.15650800	3.22212800	1.28304700
H	-8.91047500	4.49018900	1.55027600
H	-10.09367900	4.90277900	-0.58526400
H	-9.73532100	3.23585600	-1.15693700
H	6.09550300	1.80566800	4.51814200
H	3.97573900	-1.31483200	-4.16916200
C	7.03509100	3.26922700	2.31480800
C	7.95958500	3.13738000	3.53113200
H	8.89891500	3.67866600	3.34285000
H	7.50765200	3.56697100	4.43820300
H	8.21857700	2.08724700	3.73653700
C	6.74780000	4.76290400	2.07732400
H	6.12036300	4.92494200	1.18844600
H	6.22621400	5.20398700	2.94028100
H	7.68911400	5.31435700	1.92648000
C	7.75735500	2.67827900	1.09004900
H	7.94803000	1.60234700	1.22545700
H	7.16691900	2.80071600	0.17034900
H	8.72406900	3.18168100	0.93274800
C	6.17052900	-0.20859900	-2.94510700
C	7.09864200	0.43098500	-1.90524700
H	6.94612500	1.51842000	-1.83495600
H	6.94943400	-0.00196200	-0.90391600
H	8.14923800	0.26693100	-2.18875200
C	6.40290100	0.47436400	-4.30446700
H	6.15823100	1.54612600	-4.25001800
H	7.45577600	0.37546400	-4.61372500
H	5.77670400	0.02766100	-5.09140200
C	6.52372200	-1.70379900	-3.04973300
H	5.96603000	-2.20492900	-3.85407000
H	7.59673900	-1.83295100	-3.26132700
H	6.29354300	-2.22631000	-2.11005000
C	4.62894400	-3.51660400	0.60702700
C	5.66290900	-2.59846400	0.91473800
C	4.95141500	-4.74369100	-0.02086500
C	6.99028200	-2.90933300	0.58483100
C	6.28773300	-5.02832000	-0.34399700
C	7.30631200	-4.11486700	-0.04808500
H	7.78423300	-2.20700900	0.81152600
H	6.53590900	-5.96182500	-0.83494400
C	8.73212700	-4.40868100	-0.41171100

H	9.10732400	-3.62679600	-1.10545300
H	9.35822400	-4.41111200	0.50528700
H	8.83279200	-5.39681300	-0.90895700
C	3.88223900	-5.74281500	-0.37591900
H	3.35615300	-6.07894100	0.54135000
H	3.15520000	-5.27441300	-1.06763200
H	4.30407300	-6.63952600	-0.87748700
C	5.36826500	-1.29443000	1.60385100
H	4.70955900	-0.67894000	0.96243100
H	4.86333200	-1.48904300	2.57301000
H	6.29168900	-0.71020500	1.80395400

2-TS-S

Ir	0.12342800	-0.45144300	-0.79813600
B	-1.45246600	-1.24287200	0.44013900
N	-0.84713700	-2.22655700	1.38027200
C	-1.77549500	-2.73026600	2.37785300
C	-3.15368100	-2.15529400	1.91017200
N	-2.85385100	-1.24826600	0.77749900
C	0.41133900	-2.69990300	1.23782900
C	0.95920700	-3.76224600	1.99495400
C	2.27502300	-4.13154600	1.79239900
H	2.69900300	-4.94401800	2.38795600
C	3.04649900	-3.46571700	0.83006800
C	2.43203400	-2.44985400	0.11667100
H	2.97330300	-1.90482300	-0.65700800
H	0.35297000	-4.27683100	2.73873300
N	1.16614900	-2.06853200	0.31449200
C	-1.43076100	3.29315500	-2.10077100
C	-2.29431500	2.29358400	-3.01456300
O	-2.20934100	1.07477200	-2.28962000
O	-0.58577600	2.39247800	-1.40697400
B	-1.06469500	1.08444600	-1.51143200
C	-1.69648700	2.05255300	-4.41471200
H	-2.25002800	1.24080300	-4.93447000
H	-1.75759000	2.97075500	-5.03676000
H	-0.63478300	1.73393200	-4.34247500
C	-3.76337600	2.68590100	-3.18748800
H	-3.84225000	3.67808000	-3.68058000
H	-4.30112500	1.93522300	-3.80496700
H	-4.27368600	2.73280900	-2.20936500
C	-2.25705800	4.07035700	-1.06366400
H	-1.59043500	4.63070900	-0.37444700
H	-2.93795800	4.79493400	-1.55844300

H	-2.85304900	3.37180700	-0.44660700
C	-0.56612400	4.29232100	-2.87897300
H	-1.20129200	4.91164700	-3.54714000
H	-0.02366400	4.96766100	-2.18586700
H	0.20240200	3.76921700	-3.48506800
C	-0.40666000	-3.12855200	-4.04030800
C	-0.65837200	-4.05110100	-2.74571000
O	-0.97421100	-3.09271400	-1.75320700
O	-0.09869700	-1.87281400	-3.45499600
B	-0.45491700	-1.85264400	-2.10705800
C	-1.81383500	-5.04874800	-2.87151000
H	-1.91159900	-5.65514000	-1.94539500
H	-2.77698900	-4.52364800	-3.01477400
H	-1.63950000	-5.73479400	-3.72742800
C	0.59026800	-4.82104300	-2.27546100
H	0.87260400	-5.60746300	-3.00715200
H	1.44911100	-4.13143400	-2.13412000
H	0.39457700	-5.30773800	-1.29557000
C	-1.64040200	-2.93733300	-4.93224200
H	-1.42039900	-2.22290600	-5.75453800
H	-1.96611800	-3.89917000	-5.38144200
H	-2.46527300	-2.50170900	-4.34122400
C	0.76345200	-3.57195700	-4.92872500
H	0.58547100	-4.59853300	-5.31390400
H	0.88278600	-2.88348200	-5.79284900
H	1.72271700	-3.55482200	-4.37187800
C	-1.42481000	-2.33671000	3.80226500
C	-2.16115800	-2.90117200	4.85868400
C	-0.34620200	-1.48121800	4.10952500
C	-1.81678500	-2.63522700	6.18720600
H	-2.99411600	-3.56175600	4.65090800
C	0.00743800	-1.23501900	5.43925300
H	0.22600500	-1.00084300	3.32980000
C	-0.72773800	-1.81060900	6.47738700
H	-2.38845300	-3.07805100	6.99242600
H	0.84129000	-0.58322400	5.66477800
H	-0.45895300	-1.61045500	7.50641100
H	-1.75578300	-3.83558800	2.35140500
H	-3.62580500	-1.59136000	2.73219800
C	-4.08334200	-3.28506600	1.52712700
C	-5.28230800	-3.48586800	2.23568300
C	-3.72019600	-4.20845400	0.52778600
C	-6.10648200	-4.57510600	1.93647000
H	-5.57963700	-2.80334500	3.02200300

C	-4.54171900	-5.30096700	0.24010000
H	-2.79380600	-4.08843500	-0.01739900
C	-5.73692800	-5.48120700	0.93968700
H	-7.03023300	-4.71923800	2.48157800
H	-4.25414600	-6.00501800	-0.52990100
H	-6.37567000	-6.32430100	0.71089600
O	0.99117500	1.06927300	0.92967700
H	0.50042400	0.43853100	-2.07895600
P	2.25312200	1.67253500	0.38424500
C	2.96037800	0.77267100	-0.97626200
C	4.32950900	0.82750500	-1.25360800
C	2.05520400	0.00472100	-1.73485900
C	4.86656000	0.16525800	-2.36007100
H	4.98053800	1.38303200	-0.57397000
C	2.60386300	-0.61643200	-2.86554400
C	3.96252300	-0.53690400	-3.16784500
H	1.93895300	-1.18800400	-3.51265000
C	2.13012700	3.42553900	-0.01669400
C	1.21476000	4.23609200	0.65867100
C	2.98644400	3.98323700	-0.96324200
C	1.20225500	5.60009300	0.40190100
H	0.51105900	3.79743700	1.36238300
C	2.97169000	5.35294100	-1.25298300
H	3.67435800	3.33124300	-1.50430300
C	2.07290300	6.15237300	-0.54012500
H	0.49623400	6.24568300	0.92846300
O	3.48448600	1.63187200	1.49062400
C	3.28948000	2.13681600	2.77154400
H	2.30339400	2.61440400	2.89894300
C	3.33517800	0.98673800	3.79413300
C	4.35153700	3.21638000	3.02168200
C	-3.72299700	-0.14367200	0.54018300
C	-3.67926800	1.06278700	1.43525100
C	-4.73291300	-0.19777800	-0.46094100
C	-4.57801900	2.11003500	1.19482300
C	-5.61061500	0.89608200	-0.61435800
C	-5.54351600	2.04260400	0.19009300
H	-4.54093900	2.99868700	1.81139600
H	-6.37389800	0.83670300	-1.37113100
C	-4.96932500	-1.42840400	-1.33641500
C	-6.36168600	-2.03577900	-1.02498100
C	-4.84124600	-1.09183900	-2.84247200
H	-4.20165100	-2.19642200	-1.12300900
C	-6.68914500	-3.22415200	-1.93301300

H	-7.16414000	-1.27898200	-1.15441500
H	-6.39851400	-2.36617700	0.03091000
C	-5.21410500	-2.28913700	-3.72885500
H	-5.49571700	-0.24112500	-3.12371000
H	-3.79441000	-0.79883400	-3.06507600
C	-6.61331300	-2.81102000	-3.40306500
H	-7.71319800	-3.59057700	-1.70521900
H	-5.97797300	-4.05490100	-1.74405400
H	-5.17047200	-1.98624300	-4.79714500
H	-4.49031600	-3.11201000	-3.57313700
H	-6.83525700	-3.69168000	-4.04351600
H	-7.37152700	-2.02629600	-3.61900000
C	-6.49873600	3.22569000	0.03580300
C	-7.30036000	3.25326000	-1.28736700
C	-7.47483200	3.28792600	1.23027100
H	-5.87160000	4.14686200	0.04874000
C	-8.16761300	4.51508300	-1.37006700
H	-7.97260600	2.36998700	-1.35484100
H	-6.60729900	3.23934300	-2.15466300
C	-8.36625900	4.53523400	1.15102100
H	-8.11169100	2.37558600	1.24437800
H	-6.91219300	3.32319500	2.18793300
C	-9.12913900	4.59218000	-0.17801400
H	-8.75492200	4.49576200	-2.31331000
H	-7.51848800	5.41811800	-1.38938200
H	-9.09430800	4.51931700	1.99040000
H	-7.73978200	5.44787100	1.25985000
H	-9.70372200	5.54181600	-0.23087700
H	-9.85417600	3.75009700	-0.22775300
C	-2.74903400	1.16492000	2.63550400
C	-1.77592700	2.34764200	2.48923700
C	-3.55295400	1.28367900	3.95053600
H	-2.12937900	0.25362800	2.70872500
C	-0.83056700	2.42179700	3.69563300
H	-2.32541200	3.30980000	2.40572200
H	-1.18723600	2.21575700	1.55977400
C	-2.62525500	1.39645200	5.16453700
H	-4.21250400	2.17748500	3.93302700
H	-4.20029100	0.38872600	4.07451000
C	-1.62273200	2.54636200	5.00300800
H	-0.16671300	3.30578500	3.59296800
H	-0.18716000	1.51592800	3.72818600
H	-3.23504200	1.56814600	6.07744300
H	-2.08355500	0.44255600	5.30050700

H	-0.91604400	2.53811700	5.86054000
H	-2.16643300	3.51654800	5.01649200
H	4.31651700	-1.05520800	-4.06022000
H	2.02822300	7.22650600	-0.72194400
C	6.37688400	0.20994200	-2.62125100
C	6.74184600	-0.39375900	-3.98328500
H	7.82747100	-0.31560200	-4.14636600
H	6.47837800	-1.46047000	-4.04352700
H	6.23941200	0.13250900	-4.80952700
C	7.08482500	-0.60114700	-1.52091500
H	6.86062200	-0.20722100	-0.51817100
H	6.75817800	-1.65148400	-1.55221000
H	8.17755300	-0.57858300	-1.65919500
C	6.87048300	1.66779900	-2.59220100
H	6.34538300	2.27610200	-3.34537400
H	6.71715700	2.13753900	-1.60938800
H	7.94914900	1.71180700	-2.80938100
C	3.91561800	5.89345300	-2.33376900
C	3.56563700	5.22319700	-3.67592600
H	4.22635500	5.59560800	-4.47470200
H	3.67922100	4.12969500	-3.62919000
H	2.52486300	5.43922000	-3.96247000
C	5.36986300	5.55964500	-1.95266700
H	5.63294000	6.00160500	-0.97949500
H	5.53798300	4.47463900	-1.88614100
H	6.06466900	5.95559700	-2.70994400
C	3.79204800	7.41260900	-2.49893300
H	2.77743800	7.70979000	-2.80557000
H	4.04338400	7.94636400	-1.56948900
H	4.48694900	7.75960200	-3.27844000
C	4.47281600	-3.80266400	0.58550100
C	5.48982900	-2.93515100	1.05595000
C	4.83012600	-4.97356300	-0.13083200
C	6.83581700	-3.24778900	0.81507800
C	6.18431700	-5.25309700	-0.36648500
C	7.18713200	-4.39608600	0.10046700
H	7.61809200	-2.58811800	1.17262600
H	6.45600800	-6.14345300	-0.92199800
C	3.81286000	-5.94002100	-0.68365600
H	2.76905000	-5.65841500	-0.45099400
H	3.91293300	-5.99087700	-1.78842000
H	3.99366700	-6.95027000	-0.25995700
C	5.15652600	-1.68322800	1.82550900
H	4.57251100	-0.98999800	1.18662600

H	4.56194500	-1.94558900	2.72538100
H	6.07022700	-1.14734100	2.15950700
C	8.63458800	-4.69078400	-0.16353700
H	9.08394900	-3.85733500	-0.74386000
H	9.17597400	-4.79497900	0.80028400
H	8.76233900	-5.63044700	-0.74195700
F	2.63046400	-0.04132400	3.35319500
F	2.81157600	1.39018400	4.95260500
F	4.57778000	0.57078600	4.02831800
F	4.32355100	3.62174700	4.29077800
F	4.11215600	4.27210500	2.24700000
F	5.57302900	2.77864800	2.74523600

3-TS-R

Ir	-0.09181500	-0.75253200	-0.66575200
B	-1.49271300	-0.87101500	0.97489100
N	-0.73215600	-1.34779400	2.17252300
C	-1.48405600	-1.25919200	3.41293500
C	-2.93455600	-0.94395300	2.92737300
N	-2.81991100	-0.64271300	1.48196700
C	0.49321100	-1.91204800	2.10643500
C	1.15905700	-2.51622800	3.20187600
C	2.39621100	-3.10077000	3.00718300
H	2.90149100	-3.57431100	3.85281100
C	2.97707000	-3.09582600	1.73153800
C	2.27604000	-2.45713700	0.71983000
H	2.67700100	-2.41605400	-0.29253500
H	0.69598700	-2.53269500	4.18694000
N	1.09318400	-1.85595200	0.89945600
C	-2.15719100	1.90272500	-3.34169100
C	-2.96104500	0.53659900	-3.54833600
O	-2.65857900	-0.16879700	-2.35321300
O	-1.12126600	1.49516800	-2.45582600
B	-1.44916300	0.26800700	-1.84464800
C	-2.46140200	-0.30858100	-4.73658700
H	-2.94565000	-1.30847100	-4.72609300
H	-2.70043700	0.18357900	-5.70316700
H	-1.36429400	-0.47138100	-4.67429200
C	-4.47707000	0.70254100	-3.68429900
H	-4.71537000	1.39270900	-4.52116500
H	-4.96769700	-0.27323900	-3.88439000
H	-4.91198800	1.10354200	-2.75100500
C	-2.96402900	3.01014300	-2.64902500
H	-2.29920000	3.85418900	-2.36550200

H	-3.76042600	3.39890000	-3.31766600
H	-3.42219300	2.62427200	-1.71987500
C	-1.53822100	2.47903200	-4.61948700
H	-2.32384400	2.63584200	-5.38878400
H	-1.05206800	3.45479400	-4.41716700
H	-0.75858800	1.80166800	-5.02561800
C	-1.06812500	-4.28480300	-2.80370100
C	-0.97633700	-4.80600800	-1.28664400
O	-1.10491700	-3.60318800	-0.54761400
O	-0.66722500	-2.92968100	-2.67453400
B	-0.75121400	-2.51975800	-1.34315000
C	-2.08125900	-5.77909200	-0.86325400
H	-3.07585300	-5.29986500	-0.92324100
H	-1.94222300	-6.09598800	0.19292900
H	-2.07007300	-6.68122000	-1.51121900
C	0.38086100	-5.43844900	-0.92843900
H	0.53333800	-6.39243700	-1.47631800
H	1.21644300	-4.74473400	-1.16293100
H	0.42158500	-5.65292700	0.15957900
C	-2.48752200	-4.30545300	-3.38912200
H	-2.50278300	-3.82415500	-4.39038900
H	-2.86195100	-5.34554000	-3.49513700
H	-3.17337700	-3.73223900	-2.73910800
C	-0.13183900	-4.99407200	-3.79007600
H	-0.36236500	-6.08009200	-3.82532600
H	-0.24759000	-4.57221800	-4.81175200
H	0.93151300	-4.85802300	-3.50475400
C	-0.93559000	-0.23634700	4.39274100
C	-1.46566000	-0.19675800	5.69420300
C	0.14977800	0.60787200	4.07756000
C	-0.90772500	0.64198800	6.66384800
H	-2.29815100	-0.83539500	5.96344900
C	0.71789900	1.42924200	5.05549100
H	0.56242300	0.63756300	3.08063400
C	0.19085900	1.44492700	6.34847800
H	-1.31957300	0.65938300	7.66451300
H	1.55532300	2.06754000	4.80532000
H	0.62539300	2.08870200	7.10207400
H	-1.42658500	-2.23526500	3.92927600
H	-3.33814300	-0.07356300	3.46981400
C	-3.83048900	-2.13107300	3.20292800
C	-4.89929900	-2.01438000	4.11025400
C	-3.54348200	-3.39343700	2.64613100
C	-5.67337700	-3.13181500	4.43811900

H	-5.13160200	-1.06166600	4.56946600
C	-4.31205500	-4.50878300	2.98730000
H	-2.71521300	-3.51653300	1.95967700
C	-5.37978200	-4.37734800	3.87799600
H	-6.49774900	-3.03293400	5.13226600
H	-4.08343900	-5.47446100	2.55522100
H	-5.97877900	-5.24115300	4.13528400
O	0.76808200	1.27575800	0.19010200
H	0.20333100	-0.35873300	-2.19064600
P	2.17334900	1.53428100	-0.27579900
C	2.75163400	0.17466100	-1.28363000
C	4.11021800	0.04311700	-1.58093600
C	1.78353600	-0.74212700	-1.73933500
C	4.56572500	-1.03087700	-2.34682500
H	4.83173300	0.74591700	-1.15346200
C	2.24738000	-1.74658800	-2.60403500
C	3.60177100	-1.89915000	-2.88303900
H	1.52097700	-2.44124100	-3.02800600
C	3.29332300	1.94595000	1.06251900
C	4.04808600	3.11910200	1.06398800
C	3.32252100	1.08101400	2.16233100
C	4.84016900	3.45615700	2.16822800
H	4.01653200	3.77540300	0.19315900
C	4.11172700	1.40635900	3.25991100
H	2.73357400	0.16016200	2.15942400
C	4.85829100	2.58320100	3.26416000
H	4.13268800	0.74367200	4.12710200
O	2.34927200	2.87408000	-1.19950200
C	1.48378100	3.21692600	-2.24507600
H	0.53205800	2.64922400	-2.21900100
C	1.15024500	4.70043900	-2.05292900
C	2.17976800	2.91869600	-3.58399100
F	0.69143100	4.89654500	-0.82395400
F	2.22541100	5.47406100	-2.21533800
F	0.21601500	5.10499900	-2.90856100
F	2.12557700	1.61635700	-3.85161700
F	3.45943100	3.27672000	-3.56103900
F	1.58625800	3.56180200	-4.58947000
C	-3.75127000	0.26100800	0.89499600
C	-3.66108300	1.73637700	1.15216900
C	-4.97616600	-0.23588000	0.18863500
C	-4.66886600	2.57289100	0.64805900
C	-5.92674500	0.70313700	-0.24555300
C	-5.78463500	2.07905600	-0.03285900

H	-4.60026600	3.63997800	0.81191300
H	-6.81693300	0.35699700	-0.75029100
C	-5.27558000	-1.71737900	-0.00692500
C	-6.54962400	-2.11373800	0.77942900
C	-5.43611300	-2.06945300	-1.50377100
H	-4.42605700	-2.31678100	0.37422300
C	-6.92165100	-3.58361000	0.55937400
H	-7.41564200	-1.49090700	0.46899200
H	-6.39475400	-1.93593600	1.86076200
C	-5.83550900	-3.53737100	-1.70320900
H	-6.20872200	-1.43375500	-1.98470300
H	-4.47656600	-1.88986400	-2.02618600
C	-7.11155600	-3.87502300	-0.93031100
H	-7.86403600	-3.81037300	1.10274800
H	-6.12574700	-4.24181500	0.96424900
H	-5.99557800	-3.73246800	-2.78544300
H	-5.01914400	-4.19822500	-1.35178900
H	-7.34907900	-4.95206100	-1.06628100
H	-7.96348100	-3.28059900	-1.32799500
C	-2.57081700	2.35132500	2.01764800
C	-1.71300300	3.35594200	1.22364400
C	-3.18185200	3.03013500	3.26695000
H	-1.88679400	1.55876500	2.37045500
C	-0.61195500	3.94131900	2.11860200
H	-2.33712100	4.18782300	0.83206000
H	-1.25052000	2.84772800	0.35187700
C	-2.10308200	3.66529400	4.14861000
H	-3.89980800	3.82590900	2.97548600
H	-3.73882800	2.27868700	3.86694500
C	-1.21551500	4.62721800	3.35007600
H	-0.02515000	4.68842300	1.54713500
H	0.08500000	3.13598900	2.43746800
H	-2.58816100	4.21979300	4.98049700
H	-1.48399000	2.86889100	4.59699800
H	-0.39476300	4.99402700	4.00332300
H	-1.81652100	5.50622800	3.02883100
C	-6.86681400	3.04654200	-0.49054900
C	-7.05432500	3.00732200	-2.02107800
C	-8.20106000	2.76954700	0.23322000
H	-6.56261100	4.08551000	-0.22752300
C	-8.14602100	3.98892500	-2.46570800
H	-7.33230900	1.98539200	-2.35842200
H	-6.10139800	3.28728100	-2.51762300
C	-9.28604100	3.75414400	-0.22093300

H	-8.55070400	1.73382400	0.03128300
H	-8.05622000	2.87340700	1.33074200
C	-9.46889800	3.70665200	-1.74292200
H	-8.29983400	3.89387300	-3.56217000
H	-7.82029800	5.03147500	-2.25548500
H	-10.24733100	3.49363800	0.27200800
H	-9.01039700	4.78562300	0.09089600
H	-10.22121500	4.46639600	-2.04550400
H	-9.85236200	2.70635200	-2.04237600
H	5.44926800	2.84165400	4.14580300
H	3.92468400	-2.71730900	-3.53234300
C	4.27279400	-3.76832800	1.44543700
C	5.45360500	-2.99810200	1.30482000
C	4.33544900	-5.17498600	1.27300000
C	6.66128900	-3.62873400	0.97016600
C	5.55757600	-5.77801200	0.94106000
C	6.71831900	-5.01267300	0.78578700
H	7.56630500	-3.04291000	0.85689300
H	5.60192900	-6.85248400	0.80420700
C	8.02572400	-5.66263900	0.43880800
H	8.40734800	-5.24838600	-0.51704700
H	8.76635100	-5.46180500	1.24130800
H	7.91919900	-6.76256100	0.32636400
C	3.13503500	-6.07440700	1.42272600
H	3.34090500	-6.83947400	2.20067500
H	2.21762800	-5.53325300	1.72130300
H	2.93609700	-6.58874600	0.45901800
C	5.44775200	-1.51274000	1.54240100
H	4.74597400	-1.02453300	0.84110000
H	5.12811400	-1.30738300	2.58500700
H	6.45166700	-1.06236300	1.39090300
C	6.01874500	-1.26318700	-2.54235700
C	6.54639700	-2.55957700	-2.45876500
C	6.89863700	-0.19756400	-2.77580600
C	7.91296000	-2.78400800	-2.60557000
H	5.87909600	-3.39968900	-2.25062100
C	8.26606100	-0.42114700	-2.91747300
H	6.50286000	0.81699500	-2.86571800
C	8.77853000	-1.71513800	-2.83260500
H	8.30575900	-3.80037900	-2.53615300
H	8.93548800	0.42163000	-3.10324500
H	9.85067000	-1.89047400	-2.94406000
C	5.62470300	4.71873600	2.16895200
C	6.91349300	4.76633200	2.71765100

C	5.09007800	5.88987600	1.61305800
C	7.64709700	5.95043500	2.71152800
H	7.35719500	3.85852400	3.13343500
C	5.82379600	7.07346100	1.60630000
H	4.08000400	5.87954900	1.19646400
C	7.10478700	7.10821100	2.15565300
H	8.65319700	5.96603100	3.13624100
H	5.38814100	7.97648800	1.17337500
H	7.68025400	8.03632000	2.14934900

3-TS-S

Ir	-0.06819600	-0.46224700	-0.80239600
B	-1.77361600	-1.09551700	0.42897600
N	-1.24153200	-2.12214500	1.37340100
C	-2.19614300	-2.55817800	2.37674800
C	-3.53685600	-1.92771000	1.88822900
N	-3.17717900	-1.01345800	0.77624900
C	-0.01812600	-2.68380100	1.25779200
C	0.42934200	-3.78956900	2.02101500
C	1.71782800	-4.25896800	1.85484300
H	2.06205800	-5.10534800	2.45422900
C	2.55977200	-3.64351700	0.92021400
C	2.04402000	-2.58198700	0.20061800
H	2.65441900	-2.08442500	-0.54784500
H	-0.23535200	-4.26196100	2.74181200
N	0.80492600	-2.09929600	0.36326300
C	-1.60347800	3.34030100	-1.96293000
C	-2.41576700	2.35968400	-2.94821100
O	-2.39236800	1.13272500	-2.23339900
O	-0.83085600	2.42524300	-1.20178100
B	-1.27903100	1.12428200	-1.40807800
C	-1.73106700	2.13103900	-4.31159300
H	-2.25251100	1.32599600	-4.87343600
H	-1.75183100	3.05599100	-4.92619500
H	-0.67658100	1.80895900	-4.18048700
C	-3.86633600	2.76756600	-3.21400400
H	-3.90769000	3.78709000	-3.65265700
H	-4.35520100	2.05865800	-3.91566000
H	-4.45015500	2.75223000	-2.27812600
C	-2.49096500	4.13784200	-0.99723200
H	-1.86776400	4.66741200	-0.24473100
H	-3.09811100	4.89003300	-1.54383200
H	-3.16409800	3.45610000	-0.44712600
C	-0.65903200	4.31986100	-2.67041600

H	-1.22577400	4.94958700	-3.38864400
H	-0.16102500	4.98770500	-1.93730000
H	0.14499300	3.77966800	-3.21188300
C	-0.86000100	-2.98812700	-4.11267400
C	-1.10394800	-3.96782800	-2.85788400
O	-1.26347200	-3.05290200	-1.78740100
O	-0.40844700	-1.80169200	-3.47901400
B	-0.70300300	-1.83089300	-2.12061200
C	-2.35374000	-4.84784700	-2.95434500
H	-2.44099800	-5.50455300	-2.06224100
H	-3.27123000	-4.22850100	-2.98799200
H	-2.30703000	-5.48662700	-3.86168900
C	0.09914300	-4.86902300	-2.52623000
H	0.26855600	-5.61837200	-3.32814700
H	1.01975600	-4.26153400	-2.39242500
H	-0.07907600	-5.41133500	-1.57280600
C	-2.12913700	-2.63688500	-4.89968700
H	-1.90352600	-1.89253800	-5.69355900
H	-2.57098800	-3.53659900	-5.37719000
H	-2.86636700	-2.16996000	-4.22296300
C	0.20548000	-3.46629100	-5.10714800
H	-0.08674800	-4.44632400	-5.54069800
H	0.32714300	-2.73371400	-5.93393900
H	1.19671000	-3.56605100	-4.61943200
C	-1.82370600	-2.16091100	3.79595100
C	-2.60256700	-2.65387300	4.85773700
C	-0.67878100	-1.39066000	4.09647100
C	-2.23790600	-2.40394600	6.18382000
H	-3.48414000	-3.25017300	4.65617200
C	-0.30658700	-1.16254900	5.42455400
H	-0.06393000	-0.96638000	3.31529800
C	-1.08538000	-1.66825800	6.46723100
H	-2.84298700	-2.79238500	6.99267700
H	0.57731500	-0.57854600	5.64556700
H	-0.80087900	-1.48195500	7.49462100
H	-2.23654600	-3.66279500	2.37084900
H	-4.00953500	-1.36197200	2.70805300
C	-4.49539400	-3.01838400	1.46466200
C	-5.70490100	-3.20493500	2.15929300
C	-4.14646900	-3.92872400	0.44844000
C	-6.55315400	-4.26654500	1.82979800
H	-5.99128100	-2.53359600	2.95919800
C	-4.99231200	-4.99406000	0.13061300
H	-3.21201500	-3.82166200	-0.08529200

C	-6.19739900	-5.15982300	0.81668700
H	-7.48463300	-4.39958000	2.36445100
H	-4.71576000	-5.68866200	-0.65176700
H	-6.85463400	-5.98178300	0.56457100
O	0.84947900	0.95680200	0.96504500
H	-0.15837200	0.38785900	-2.12785200
P	2.21139000	1.41162600	0.52164300
C	2.87607300	0.45809400	-0.82404700
C	4.25952500	0.33515000	-1.00467500
C	1.92673300	-0.23097300	-1.60275400
C	4.76583800	-0.47914800	-2.01679400
H	4.94490600	0.84236100	-0.32183700
C	2.46419600	-0.93102800	-2.69995800
C	3.83564100	-1.06677200	-2.89163500
H	1.77952500	-1.44426000	-3.37589300
C	2.29185500	3.18521900	0.19702900
C	1.26823800	4.01439700	0.65903100
C	3.37019200	3.72708000	-0.50680600
C	1.34402100	5.38604500	0.43296100
H	0.41382400	3.57618300	1.16696500
C	3.44241500	5.09974800	-0.76436500
H	4.17106600	3.07689500	-0.86405100
C	2.41583700	5.92139300	-0.27609600
H	0.54436900	6.03960900	0.78735100
O	3.38810500	1.17580700	1.65261300
C	3.59121600	1.93921300	2.78787600
H	3.09644400	2.92460400	2.73338200
C	3.01859600	1.21466500	4.04189100
C	5.09842200	2.21781200	2.86453600
C	-4.00660600	0.12802600	0.55893500
C	-3.96301000	1.24504500	1.44435600
C	-5.07625200	0.11167900	-0.49985200
C	-4.85776800	2.31531600	1.25657500
C	-5.93210900	1.22156400	-0.60322600
C	-5.83471300	2.31349500	0.26102400
H	-4.80143000	3.17557100	1.90955100
H	-6.71046300	1.22995200	-1.35254100
C	-5.35501200	-1.09877600	-1.38364000
C	-6.75693900	-1.67506800	-1.05810700
C	-5.24830900	-0.74303500	-2.88609200
H	-4.59765500	-1.88161000	-1.18565400
C	-7.12666900	-2.84464700	-1.97442700
H	-7.54114900	-0.89605000	-1.16886600
H	-6.78807600	-2.01474700	-0.00543500

C	-5.65988900	-1.92036500	-3.78283500
H	-5.89399100	0.12342900	-3.13981600
H	-4.20205200	-0.46283800	-3.12416600
C	-7.06482700	-2.41612400	-3.44082100
H	-8.15512700	-3.19005400	-1.73402200
H	-6.43186600	-3.69350000	-1.80725100
H	-5.62742900	-1.60287900	-4.84724500
H	-4.95201400	-2.76196100	-3.65210100
H	-7.31589100	-3.28414400	-4.08771000
H	-7.80921100	-1.61281500	-3.63557400
C	-6.81035200	3.47749900	0.16461400
C	-6.69690600	4.20405400	-1.19063400
C	-8.25881800	3.01114300	0.41922900
H	-6.56530000	4.22434400	0.95417000
C	-7.68508700	5.37469400	-1.27118000
H	-6.89897500	3.50347800	-2.02981500
H	-5.66692000	4.60286100	-1.31062400
C	-9.23675500	4.18980300	0.33534700
H	-8.56099100	2.24199900	-0.32425600
H	-8.32926200	2.55519400	1.43089800
C	-9.12207000	4.90299900	-1.01747100
H	-7.62368000	5.83731700	-2.27970300
H	-7.40875200	6.14967800	-0.52263000
H	-10.27484700	3.81523300	0.46547900
H	-9.02749600	4.90975700	1.15700700
H	-9.80305800	5.78101800	-1.02769900
H	-9.43847400	4.21411100	-1.83145100
C	-3.00202100	1.32603100	2.62646900
C	-2.00097100	2.47738300	2.43846200
C	-3.76537900	1.47557400	3.96535300
H	-2.40153700	0.40247700	2.69855700
C	-1.02729100	2.54424800	3.61925700
H	-2.52348300	3.45367400	2.34491900
H	-1.43370400	2.30221000	1.50293600
C	-2.80383400	1.59387500	5.15217400
H	-4.41246800	2.37733900	3.96143200
H	-4.42135400	0.59206400	4.12074000
C	-1.78339200	2.71917100	4.94116400
H	-0.33369000	3.40063500	3.48068400
H	-0.42294900	1.61261000	3.65292900
H	-3.38517700	1.79524600	6.07753100
H	-2.27727300	0.63326700	5.29266100
H	-1.05598800	2.71497400	5.78125400
H	-2.30592400	3.70097100	4.94442400

H	4.19555300	-1.69938700	-3.70769200
H	2.44393200	6.99385200	-0.48230300
C	3.96487200	-4.06972800	0.69865800
C	5.02178900	-3.31745100	1.26712000
C	4.26414000	-5.17573200	-0.13604500
C	6.35130100	-3.67164100	0.99428700
C	5.60330700	-5.50041900	-0.39854300
C	6.64677600	-4.75132300	0.15747600
H	7.16443000	-3.09694200	1.42263200
H	5.83130200	-6.33889200	-1.04669800
C	3.19881100	-6.02023100	-0.78828500
H	2.17193500	-5.72622300	-0.49979700
H	3.28403900	-5.93618100	-1.89225400
H	3.33836500	-7.08261600	-0.49720300
C	4.74548600	-2.13504300	2.15960600
H	4.20567700	-1.35403200	1.58778500
H	4.12509500	-2.45207400	3.02398700
H	5.68215600	-1.68603100	2.55232100
C	8.07847300	-5.08946500	-0.13883000
H	8.57224700	-4.22363900	-0.62814700
H	8.61028600	-5.32301700	0.80745400
H	8.16036400	-5.96747400	-0.81423100
C	4.57304900	5.65986100	-1.54966300
C	5.15187300	6.88906900	-1.20589300
C	5.08416600	4.96521500	-2.65513400
C	6.21073000	7.40953000	-1.94620600
H	4.78350700	7.43441200	-0.33366500
C	6.14374900	5.48471600	-3.39446500
H	4.63399000	4.01499800	-2.95246400
C	6.71045600	6.70908800	-3.04317300
H	6.65398100	8.36529500	-1.65827700
H	6.52458500	4.93078100	-4.25513900
H	7.54139000	7.11652400	-3.62290300
C	6.22209500	-0.75671100	-2.11498400
C	6.85133400	-0.95170600	-3.35231600
C	6.99942100	-0.86489400	-0.95176300
C	8.20949800	-1.25350600	-3.42393700
H	6.27311300	-0.85124900	-4.27373500
C	8.35695000	-1.16506800	-1.02228800
H	6.52980900	-0.73447300	0.02580800
C	8.96788900	-1.36442800	-2.25934700
H	8.68022000	-1.39856400	-4.39892100
H	8.93776400	-1.25603100	-0.10173200
H	10.03153400	-1.60552000	-2.31557500

F	2.62176100	-0.00902100	3.74812500
F	3.91192600	1.13512700	5.02940600
F	1.97337100	1.89670600	4.50995200
F	5.49411200	2.79909600	1.73122100
F	5.80609200	1.10414200	3.01549700
F	5.37773900	3.04583700	3.86659000

Ir	0.38653900	-1.31527200	-0.23106800
B	2.18516900	-0.36454900	-0.05116600
N	3.07040700	-0.65097800	-1.22389900
C	4.24959500	0.20156800	-1.24939300
H	5.15614100	-0.40642100	-1.41650100
C	4.26333100	0.85133700	0.17245400
H	4.45453900	1.92987000	0.06816200
N	2.91717300	0.60506000	0.69959900
C	2.81925100	-1.65270300	-2.09420900
C	3.71595100	-2.08451100	-3.10134700
C	3.38254800	-3.18288900	-3.87137000
H	4.07446400	-3.53161700	-4.64164300
C	2.17069500	-3.84832400	-3.65359500
C	1.33163000	-3.34088500	-2.67512300
H	0.36830100	-3.80911800	-2.46241100
C	2.55769100	1.07658200	1.98620100
C	2.05921900	2.02801800	4.58312700
H	1.86677800	2.39285100	5.58366000
C	1.94418000	2.89072800	3.49222700
H	1.66868300	3.92475800	3.66082400
H	4.66421100	-1.57045100	-3.25231500
N	1.62268300	-2.26587800	-1.94123100
C	2.73524500	-4.11444900	2.14573400
C	1.24194000	-4.68183700	2.30590000
O	0.52154100	-3.89238600	1.37561500
O	2.50884600	-2.82672000	1.60237400
B	1.23423500	-2.73076800	1.06512200
C	-2.25219600	0.48632200	2.59740800
C	-1.49390400	-0.56737200	3.52848200
O	-0.33476300	-0.85224400	2.76182000
O	-1.77895600	0.12730700	1.31205200
B	-0.58055600	-0.60332900	1.40767300
C	2.67209600	0.22295300	3.09652000
H	2.99173700	-0.79939100	2.96860100
C	-3.24953800	-0.60884400	-2.55512000
H	-3.11166600	0.46160800	-2.78288000
H	-3.80646000	-1.01681600	-3.41543900

C	-1.82302000	-1.21385500	-2.44380200
C	-1.87924400	-2.57848400	-1.71886400
O	-1.22636400	-2.72232500	-0.67391900
N	-2.61513000	-3.64012700	-2.12411700
C	-2.64581400	-4.84795800	-1.26007900
H	-3.31367000	-5.53971500	-1.79139600
C	-3.36446800	-3.72110800	-3.38384800
H	-3.25429000	-2.75415400	-3.87563300
C	-0.98837800	-0.18553500	-1.60414900
H	-1.66114700	0.35728300	-0.93207000
H	0.37597000	0.16814900	-0.84501600
H	-0.63106000	0.54532300	-2.34622300
C	-2.75620100	-4.76769200	-4.31918900
H	-1.68630600	-4.56771900	-4.48032300
H	-2.85659600	-5.78767800	-3.91824900
H	-3.26300200	-4.74228000	-5.29505200
C	-4.86341300	-3.93229000	-3.16773600
H	-5.38331200	-3.90009400	-4.13637300
H	-5.08783700	-4.90475800	-2.70509200
H	-5.28444500	-3.14339300	-2.52924500
C	-1.29302700	-5.55068700	-1.13020600
H	-0.84652100	-5.73232500	-2.11983700
H	-0.59798700	-4.96939100	-0.50939500
H	-1.44461700	-6.53044500	-0.65195000
C	-3.28448900	-4.57346200	0.10199700
H	-4.25067700	-4.05954600	-0.01380900
H	-3.46623300	-5.52905100	0.61630600
H	-2.62313900	-3.96300500	0.72887600
C	-1.15711000	-1.32012100	-3.82996200
H	-0.07550200	-1.46864500	-3.72347200
H	-1.54679600	-2.12523200	-4.46712400
H	-1.30970600	-0.37128600	-4.36626900
C	2.19529400	2.42783600	2.19181900
C	4.14821400	1.23810200	-2.35033500
C	5.20202700	1.39896600	-3.26787100
C	3.00846300	2.05714800	-2.47216100
C	5.11714500	2.35694200	-4.28272000
H	6.09265400	0.78737100	-3.19668200
C	2.92790200	3.01111100	-3.49028800
H	2.18177300	1.95969700	-1.78226600
C	3.98168400	3.16272400	-4.39327100
H	5.93414900	2.47641200	-4.98234800
H	2.04924200	3.63593600	-3.57779900
H	3.91920800	3.90566500	-5.17769600

C	5.34299600	0.27035100	1.06347800
C	6.23738100	1.12473000	1.73407000
C	5.46086900	-1.12014800	1.24914800
C	7.22986500	0.59832900	2.56589600
H	6.16491000	2.19900200	1.61969100
C	6.46235300	-1.64162100	2.07262700
H	4.79026400	-1.80080200	0.74354900
C	7.34358500	-0.78347700	2.73347700
H	7.91299100	1.26259600	3.07927800
H	6.55918600	-2.71227900	2.19490500
H	8.11629500	-1.18942600	3.37338600
C	-2.27270700	-1.88170200	3.73333200
H	-1.65207800	-2.61749600	4.28474900
H	-3.19751000	-1.70722100	4.32318900
H	-2.54546600	-2.33628900	2.75727800
C	-1.08586000	-0.03829400	4.90739700
H	-0.42059100	0.84085600	4.82118900
H	-1.98655300	0.25367000	5.48828700
H	-0.52905800	-0.81549700	5.47394600
C	-3.78114600	0.38862200	2.61518900
H	-4.16756900	0.56589800	3.64130000
H	-4.22680500	1.14108400	1.92937300
H	-4.11834000	-0.60909700	2.26852500
C	-1.85839200	1.94977500	2.86259000
H	-0.75603400	2.06794600	2.83484400
H	-2.28595700	2.61138000	2.07932100
H	-2.23461700	2.28938900	3.85098000
C	1.06490800	-6.16167800	1.94432500
H	1.70807100	-6.79588000	2.59070300
H	0.00652000	-6.47295600	2.07954700
H	1.32030200	-6.34688600	0.88049700
C	0.63295700	-4.44845600	3.69951500
H	0.69959200	-3.37634600	3.98161700
H	-0.44220600	-4.72906300	3.70138700
H	1.15253700	-5.05982400	4.46745900
C	3.51750500	-3.97717300	3.45753000
H	3.59746900	-4.96306300	3.96276900
H	4.54138900	-3.59536100	3.26624200
H	3.02863900	-3.25325900	4.14174800
C	3.59898200	-4.90226400	1.14406600
H	4.56789600	-4.38455000	0.97701000
H	3.81173300	-5.92481900	1.52174300
H	3.08960800	-4.97362000	0.15927800
C	2.42097100	0.69539000	4.38521000

H	1.88435600	-4.72966900	-4.22740300
H	2.51512600	0.02891300	5.23266500
C	2.12016800	3.38586000	1.07577100
C	0.92258200	3.57140900	0.38395500
C	3.23879900	4.15290500	0.75034900
C	0.82259800	4.51962500	-0.65500800
H	0.06318100	2.98399000	0.67601000
C	3.21430500	5.06014600	-0.32443100
H	4.14264400	4.01283000	1.32878900
C	1.98881400	5.24127400	-1.00872200
H	1.94164200	5.94899300	-1.81430600
C	4.50783200	5.81491500	-0.70179900
C	5.62772100	4.79783700	-1.01937500
H	5.89401600	4.18632700	-0.13282200
H	5.30709100	4.11216500	-1.83085500
H	6.55310200	5.31818900	-1.34865200
C	4.94564500	6.70342900	0.48197600
H	5.85515400	7.28717100	0.22147200
H	4.13634400	7.41711900	0.74877700
H	5.18494600	6.09694700	1.38050500
C	4.34475800	6.72607900	-1.94235700
H	3.59579300	7.52529300	-1.75364600
H	5.30491400	7.22793600	-2.19200400
H	4.03715000	6.13226300	-2.83062800
C	-0.54218800	4.75064000	-1.34124800
C	-1.05382600	3.42528000	-1.94161200
H	-1.27106200	2.68488600	-1.14693300
H	-1.99769100	3.58192000	-2.50745600
H	-0.29760100	2.99669000	-2.63366800
C	-0.48719200	5.79041800	-2.48681200
H	-1.48845600	5.91649800	-2.95340100
H	-0.17450200	6.78648800	-2.10533700
H	0.21320500	5.46339000	-3.28539000
C	-1.55629600	5.26048300	-0.29470900
H	-1.71635100	4.51681100	0.51329100
H	-1.19231500	6.20309500	0.16844500
H	-2.54330200	5.45983900	-0.76578500
C	-4.09728100	-0.75338600	-1.29236000
H	-4.36904300	-1.81283800	-1.15498600
H	-3.49849500	-0.48656900	-0.40540500
C	-5.36820400	0.09138800	-1.32472000
H	-5.11065300	1.15094200	-1.15988500
H	-5.83184000	0.04415400	-2.32643000
C	-6.39672600	-0.35147900	-0.27706000

H	-6.80015700	-1.33809600	-0.56423900
H	-5.89095100	-0.49919500	0.68810200
C	-7.52713500	0.62841400	-0.09548300
C	-7.57571500	1.46534800	0.99598800
C	-8.54949100	0.73621500	-1.08002400
C	-8.61062700	2.42552000	1.15956600
H	-6.80230000	1.39856300	1.76661200
C	-9.56383600	1.65112700	-0.95536000
H	-8.52259800	0.06981300	-1.94687400
C	-8.66476500	3.29223300	2.28543700
C	-9.62561500	2.52453600	0.16553100
H	-10.34290400	1.71698000	-1.71921300
C	-9.67575700	4.21256500	2.41607200
H	-7.88500100	3.21540200	3.04748300
C	-10.66011100	3.48581900	0.32889900
C	-10.68522200	4.31098700	1.42684900
H	-9.70484500	4.87438800	3.28451300
H	-11.43743400	3.55813400	-0.43615000
H	-11.48360000	5.04751100	1.54081600

4-TS-R

Ir	0.38653900	-1.31527200	-0.23106800
B	2.18516900	-0.36454900	-0.05116600
N	3.07040700	-0.65097800	-1.22389900
C	4.24959500	0.20156800	-1.24939300
H	5.15614100	-0.40642100	-1.41650100
C	4.26333100	0.85133700	0.17245400
H	4.45453900	1.92987000	0.06816200
N	2.91717300	0.60506000	0.69959900
C	2.81925100	-1.65270300	-2.09420900
C	3.71595100	-2.08451100	-3.10134700
C	3.38254800	-3.18288900	-3.87137000
H	4.07446400	-3.53161700	-4.64164300
C	2.17069500	-3.84832400	-3.65359500
C	1.33163000	-3.34088500	-2.67512300
H	0.36830100	-3.80911800	-2.46241100
C	2.55769100	1.07658200	1.98620100
C	2.05921900	2.02801800	4.58312700
H	1.86677800	2.39285100	5.58366000
C	1.94418000	2.89072800	3.49222700
H	1.66868300	3.92475800	3.66082400
H	4.66421100	-1.57045100	-3.25231500
N	1.62268300	-2.26587800	-1.94123100
C	2.73524500	-4.11444900	2.14573400

C	1.24194000	-4.68183700	2.30590000
O	0.52154100	-3.89238600	1.37561500
O	2.50884600	-2.82672000	1.60237400
B	1.23423500	-2.73076800	1.06512200
C	-2.25219600	0.48632200	2.59740800
C	-1.49390400	-0.56737200	3.52848200
O	-0.33476300	-0.85224400	2.76182000
O	-1.77895600	0.12730700	1.31205200
B	-0.58055600	-0.60332900	1.40767300
C	2.67209600	0.22295300	3.09652000
H	2.99173700	-0.79939100	2.96860100
C	-3.24953800	-0.60884400	-2.55512000
H	-3.11166600	0.46160800	-2.78288000
H	-3.80646000	-1.01681600	-3.41543900
C	-1.82302000	-1.21385500	-2.44380200
C	-1.87924400	-2.57848400	-1.71886400
O	-1.22636400	-2.72232500	-0.67391900
N	-2.61513000	-3.64012700	-2.12411700
C	-2.64581400	-4.84795800	-1.26007900
H	-3.31367000	-5.53971500	-1.79139600
C	-3.36446800	-3.72110800	-3.38384800
H	-3.25429000	-2.75415400	-3.87563300
C	-0.98837800	-0.18553500	-1.60414900
H	-1.66114700	0.35728300	-0.93207000
H	0.37597000	0.16814900	-0.84501600
H	-0.63106000	0.54532300	-2.34622300
C	-2.75620100	-4.76769200	-4.31918900
H	-1.68630600	-4.56771900	-4.48032300
H	-2.85659600	-5.78767800	-3.91824900
H	-3.26300200	-4.74228000	-5.29505200
C	-4.86341300	-3.93229000	-3.16773600
H	-5.38331200	-3.90009400	-4.13637300
H	-5.08783700	-4.90475800	-2.70509200
H	-5.28444500	-3.14339300	-2.52924500
C	-1.29302700	-5.55068700	-1.13020600
H	-0.84652100	-5.73232500	-2.11983700
H	-0.59798700	-4.96939100	-0.50939500
H	-1.44461700	-6.53044500	-0.65195000
C	-3.28448900	-4.57346200	0.10199700
H	-4.25067700	-4.05954600	-0.01380900
H	-3.46623300	-5.52905100	0.61630600
H	-2.62313900	-3.96300500	0.72887600
C	-1.15711000	-1.32012100	-3.82996200
H	-0.07550200	-1.46864500	-3.72347200

H	-1.54679600	-2.12523200	-4.46712400
H	-1.30970600	-0.37128600	-4.36626900
C	2.19529400	2.42783600	2.19181900
C	4.14821400	1.23810200	-2.35033500
C	5.20202700	1.39896600	-3.26787100
C	3.00846300	2.05714800	-2.47216100
C	5.11714500	2.35694200	-4.28272000
H	6.09265400	0.78737100	-3.19668200
C	2.92790200	3.01111100	-3.49028800
H	2.18177300	1.95969700	-1.78226600
C	3.98168400	3.16272400	-4.39327100
H	5.93414900	2.47641200	-4.98234800
H	2.04924200	3.63593600	-3.57779900
H	3.91920800	3.90566500	-5.17769600
C	5.34299600	0.27035100	1.06347800
C	6.23738100	1.12473000	1.73407000
C	5.46086900	-1.12014800	1.24914800
C	7.22986500	0.59832900	2.56589600
H	6.16491000	2.19900200	1.61969100
C	6.46235300	-1.64162100	2.07262700
H	4.79026400	-1.80080200	0.74354900
C	7.34358500	-0.78347700	2.73347700
H	7.91299100	1.26259600	3.07927800
H	6.55918600	-2.71227900	2.19490500
H	8.11629500	-1.18942600	3.37338600
C	-2.27270700	-1.88170200	3.73333200
H	-1.65207800	-2.61749600	4.28474900
H	-3.19751000	-1.70722100	4.32318900
H	-2.54546600	-2.33628900	2.75727800
C	-1.08586000	-0.03829400	4.90739700
H	-0.42059100	0.84085600	4.82118900
H	-1.98655300	0.25367000	5.48828700
H	-0.52905800	-0.81549700	5.47394600
C	-3.78114600	0.38862200	2.61518900
H	-4.16756900	0.56589800	3.64130000
H	-4.22680500	1.14108400	1.92937300
H	-4.11834000	-0.60909700	2.26852500
C	-1.85839200	1.94977500	2.86259000
H	-0.75603400	2.06794600	2.83484400
H	-2.28595700	2.61138000	2.07932100
H	-2.23461700	2.28938900	3.85098000
C	1.06490800	-6.16167800	1.94432500
H	1.70807100	-6.79588000	2.59070300
H	0.00652000	-6.47295600	2.07954700

H	1.32030200	-6.34688600	0.88049700
C	0.63295700	-4.44845600	3.69951500
H	0.69959200	-3.37634600	3.98161700
H	-0.44220600	-4.72906300	3.70138700
H	1.15253700	-5.05982400	4.46745900
C	3.51750500	-3.97717300	3.45753000
H	3.59746900	-4.96306300	3.96276900
H	4.54138900	-3.59536100	3.26624200
H	3.02863900	-3.25325900	4.14174800
C	3.59898200	-4.90226400	1.14406600
H	4.56789600	-4.38455000	0.97701000
H	3.81173300	-5.92481900	1.52174300
H	3.08960800	-4.97362000	0.15927800
C	2.42097100	0.69539000	4.38521000
H	1.88435600	-4.72966900	-4.22740300
H	2.51512600	0.02891300	5.23266500
C	2.12016800	3.38586000	1.07577100
C	0.92258200	3.57140900	0.38395500
C	3.23879900	4.15290500	0.75034900
C	0.82259800	4.51962500	-0.65500800
H	0.06318100	2.98399000	0.67601000
C	3.21430500	5.06014600	-0.32443100
H	4.14264400	4.01283000	1.32878900
C	1.98881400	5.24127400	-1.00872200
H	1.94164200	5.94899300	-1.81430600
C	4.50783200	5.81491500	-0.70179900
C	5.62772100	4.79783700	-1.01937500
H	5.89401600	4.18632700	-0.13282200
H	5.30709100	4.11216500	-1.83085500
H	6.55310200	5.31818900	-1.34865200
C	4.94564500	6.70342900	0.48197600
H	5.85515400	7.28717100	0.22147200
H	4.13634400	7.41711900	0.74877700
H	5.18494600	6.09694700	1.38050500
C	4.34475800	6.72607900	-1.94235700
H	3.59579300	7.52529300	-1.75364600
H	5.30491400	7.22793600	-2.19200400
H	4.03715000	6.13226300	-2.83062800
C	-0.54218800	4.75064000	-1.34124800
C	-1.05382600	3.42528000	-1.94161200
H	-1.27106200	2.68488600	-1.14693300
H	-1.99769100	3.58192000	-2.50745600
H	-0.29760100	2.99669000	-2.63366800
C	-0.48719200	5.79041800	-2.48681200

H	-1.48845600	5.91649800	-2.95340100
H	-0.17450200	6.78648800	-2.10533700
H	0.21320500	5.46339000	-3.28539000
C	-1.55629600	5.26048300	-0.29470900
H	-1.71635100	4.51681100	0.51329100
H	-1.19231500	6.20309500	0.16844500
H	-2.54330200	5.45983900	-0.76578500
C	-4.09728100	-0.75338600	-1.29236000
H	-4.36904300	-1.81283800	-1.15498600
H	-3.49849500	-0.48656900	-0.40540500
C	-5.36820400	0.09138800	-1.32472000
H	-5.11065300	1.15094200	-1.15988500
H	-5.83184000	0.04415400	-2.32643000
C	-6.39672600	-0.35147900	-0.27706000
H	-6.80015700	-1.33809600	-0.56423900
H	-5.89095100	-0.49919500	0.68810200
C	-7.52713500	0.62841400	-0.09548300
C	-7.57571500	1.46534800	0.99598800
C	-8.54949100	0.73621500	-1.08002400
C	-8.61062700	2.42552000	1.15956600
H	-6.80230000	1.39856300	1.76661200
C	-9.56383600	1.65112700	-0.95536000
H	-8.52259800	0.06981300	-1.94687400
C	-8.66476500	3.29223300	2.28543700
C	-9.62561500	2.52453600	0.16553100
H	-10.34290400	1.71698000	-1.71921300
C	-9.67575700	4.21256500	2.41607200
H	-7.88500100	3.21540200	3.04748300
C	-10.66011100	3.48581900	0.32889900
C	-10.68522200	4.31098700	1.42684900
H	-9.70484500	4.87438800	3.28451300
H	-11.43743400	3.55813400	-0.43615000
H	-11.48360000	5.04751100	1.54081600

4-TS-S

Ir	0.14936400	0.77818700	0.16627800
B	-1.60883200	0.32838500	-0.77266200
N	-1.35478500	-0.47693600	-2.01450900
C	-2.57890000	-1.02079800	-2.58167400
H	-2.61495500	-0.82378000	-3.66751600
C	-3.70686600	-0.24081600	-1.83482600
H	-4.48541600	-0.95218600	-1.52087400
N	-3.03466400	0.35111300	-0.67208400
C	-0.14189700	-0.53572100	-2.60562700

C	0.10770200	-1.08260800	-3.88757100
C	1.37251400	-0.95883800	-4.43276900
H	1.57136900	-1.36257200	-5.42838500
C	2.38488500	-0.30718700	-3.71776400
C	2.07537800	0.14735100	-2.44575400
H	2.81766700	0.64999200	-1.82027900
C	-3.77439800	1.14100900	0.24265900
C	-5.42745600	2.72993600	1.86824500
H	-6.06233800	3.34330700	2.49421000
C	-5.42692700	1.34269000	2.02201100
H	-6.06813900	0.89172900	2.76969200
H	-0.69048000	-1.57097800	-4.44498100
N	0.87223200	-0.00030300	-1.89004200
C	-0.43826900	4.31944100	-2.30908800
C	0.74012700	4.76510000	-1.31536100
O	1.06288700	3.54391900	-0.67385100
O	-0.92719600	3.14658900	-1.68425600
B	0.00178200	2.63973500	-0.78960000
C	-1.01838400	2.10706400	4.06093300
C	-1.05663600	3.48584400	3.24734300
O	-1.08672000	3.03376700	1.90367300
O	-0.44464300	1.22670100	3.11299100
B	-0.56044200	1.74026200	1.81293200
C	-3.79035300	2.53886200	0.09971200
H	-3.19835700	3.01626100	-0.66531300
C	2.86657100	-1.74854200	2.61904500
H	2.11289200	-2.49989800	2.89495400
H	3.82146500	-2.28496900	2.52776100
C	2.40095000	-1.05655700	1.32024500
C	2.98839400	0.35813400	1.08655000
O	2.24024500	1.20529800	0.56648000
N	4.24067700	0.78151200	1.36514900
C	4.55834500	2.21037800	1.10862600
H	5.58069700	2.32558300	1.49190400
C	5.33454600	-0.03552800	1.90564600
H	4.98194000	-1.06584300	1.89269200
C	0.84924900	-0.92362400	1.46060600
H	0.60764400	-0.62041100	2.48717700
H	-0.55689900	-0.54186600	0.75085100
H	0.48615100	-1.95676300	1.34091800
C	6.58189600	-0.00409000	1.02273200
H	6.33532600	-0.25010000	-0.01795500
H	7.09258600	0.96983500	1.03869600
H	7.30299600	-0.75685700	1.37429000

C	5.64826400	0.33656100	3.35450500
H	6.40589700	-0.34829000	3.76332800
H	6.04807200	1.35928200	3.43357500
H	4.74851800	0.27330300	3.98258600
C	4.59060600	2.55564400	-0.38214700
H	5.16292800	1.80651100	-0.95050500
H	3.57169700	2.63053000	-0.78529200
H	5.08316600	3.53050100	-0.51885300
C	3.68081000	3.18613500	1.89600400
H	3.54653800	2.84237000	2.93253900
H	4.17727000	4.16813400	1.92549200
H	2.69842300	3.30568100	1.42165500
C	2.65966900	-1.97836500	0.09724900
H	1.90228000	-1.74495000	-0.66187300
H	2.43584000	-3.00525000	0.43458200
C	-4.60657100	0.53982200	1.21503900
C	-2.66706300	-2.51606800	-2.35360800
C	-2.91488800	-3.38222300	-3.43382400
C	-2.50855000	-3.06304200	-1.06505300
C	-3.00247400	-4.76261900	-3.22878700
H	-3.04541200	-2.99115700	-4.43503600
C	-2.59503300	-4.44366900	-0.86641500
H	-2.31819800	-2.42473400	-0.21350700
C	-2.84362100	-5.29261300	-1.94635300
H	-3.19652000	-5.42177500	-4.06495400
H	-2.47231500	-4.85610200	0.12601400
H	-2.91468000	-6.36101800	-1.78920400
C	-4.35812100	0.80870000	-2.71350200
C	-5.75778200	0.84165600	-2.85093700
C	-3.58469800	1.77512000	-3.38462900
C	-6.36789000	1.81740800	-3.64443700
H	-6.37957800	0.11797400	-2.33887900
C	-4.19915100	2.74321900	-4.18322000
H	-2.50689700	1.77184000	-3.30114300
C	-5.58947700	2.76644900	-4.31085100
H	-7.44551200	1.83724900	-3.74302000
H	-3.59698900	3.47379200	-4.70699100
H	-6.06360900	3.51870800	-4.92778900
C	0.20424300	4.34897700	3.44064700
H	0.19174500	5.21531900	2.74714300
H	0.26286400	4.73949500	4.47883300
H	1.11860200	3.76021700	3.22649000
C	-2.27939400	4.36878600	3.52003000
H	-3.22017500	3.84096700	3.27573800

H	-2.30657400	4.66806400	4.58945600
H	-2.24630100	5.28497200	2.89180000
C	-0.14998500	2.11872400	5.32506300
H	-0.50882100	2.89747700	6.03120900
H	-0.18762300	1.13175900	5.83480000
H	0.91435400	2.31231300	5.07783200
C	-2.40910100	1.56721300	4.43599800
H	-3.05495400	1.50498000	3.53773600
H	-2.32560300	0.54299000	4.85762200
H	-2.89775000	2.21775500	5.19210400
C	1.99743800	5.31455100	-1.99985600
H	1.74536400	6.20942300	-2.60759200
H	2.76054200	5.60008500	-1.24375700
H	2.46404000	4.54833200	-2.65299900
C	0.29771700	5.76856300	-0.23561700
H	-0.58852000	5.38807300	0.31506400
H	1.11182900	5.91938900	0.50553600
H	0.05060700	6.75309600	-0.68620200
C	-1.58323000	5.32959500	-2.44909000
H	-1.19546100	6.30166700	-2.82102500
H	-2.34907300	4.95839500	-3.16161300
H	-2.09817500	5.48750500	-1.47891000
C	0.04702700	3.93371000	-3.71846900
H	-0.78841700	3.50229200	-4.31068800
H	0.43645000	4.82119000	-4.26081300
H	0.84344400	3.16105900	-3.66082800
C	-4.61034200	3.32681000	0.90797400
H	3.38287700	-0.16379000	-4.13198100
H	-4.61694600	4.40202900	0.78482600
C	-4.65449600	-0.92149300	1.39150200
C	-3.75666900	-1.55541000	2.25118700
C	-5.63335600	-1.66608800	0.73350500
C	-3.81193300	-2.94835200	2.46216300
H	-3.02625200	-0.94953900	2.76962200
C	-5.68695500	-3.06705300	0.85224600
H	-6.33635200	-1.14218800	0.09910700
C	-4.77427400	-3.69128400	1.73578700
H	-4.81600800	-4.75646200	1.86126400
C	-6.72823100	-3.85325500	0.02595800
C	-6.52708800	-3.55607800	-1.47754900
H	-6.70831500	-2.48758400	-1.71524900
H	-5.49136600	-3.80951000	-1.78542500
H	-7.23051000	-4.15405000	-2.09653900
C	-8.14876300	-3.42031200	0.44694300

H	-8.91820100	-4.00052200	-0.10728400
H	-8.29787400	-3.59176800	1.53501400
H	-8.32675400	-2.34496900	0.23641900
C	-6.62651600	-5.38650900	0.21253500
H	-6.82566900	-5.67318100	1.26776800
H	-7.37712900	-5.91116000	-0.41787100
H	-5.62156300	-5.75518300	-0.08818800
C	-2.84917200	-3.59887000	3.48032100
C	-1.38981900	-3.30044300	3.07991400
H	-1.16951600	-2.21618300	3.14616400
H	-0.67703900	-3.81998700	3.75638100
H	-1.19706000	-3.64176200	2.04026000
C	-3.00111700	-5.13655000	3.57583000
H	-2.28475900	-5.55856700	4.31400100
H	-4.02301400	-5.41513000	3.91285400
H	-2.79069800	-5.61724500	2.59610700
C	-3.12226300	-3.01378100	4.88269600
H	-2.93590300	-1.92016100	4.91113900
H	-4.17883400	-3.19386800	5.17722900
H	-2.46166700	-3.48498100	5.64258700
H	2.93822000	-1.03815300	3.45526900
C	4.02615800	-1.96674200	-0.57249500
H	4.25860200	-0.95334100	-0.94187800
H	4.82245000	-2.21769500	0.14611400
C	4.08379800	-2.94895100	-1.74016700
H	3.99051600	-3.97679700	-1.34858800
H	3.20616900	-2.79396400	-2.38963300
C	5.34741900	-2.82146500	-2.60027100
H	5.37011100	-3.64791900	-3.33067200
H	5.28831100	-1.89131600	-3.18797700
C	6.63080700	-2.81000100	-1.80484100
C	7.53284200	-1.77736300	-1.91326300
C	6.90850400	-3.85827500	-0.88082900
C	8.70873100	-1.71894200	-1.11538000
H	7.33769200	-0.95919400	-2.61315100
C	8.02974700	-3.83424800	-0.09309200
H	6.20420500	-4.68951300	-0.79231200
C	9.62427200	-0.63510900	-1.19805500
C	8.95896000	-2.75988300	-0.17754100
H	8.22128300	-4.64231700	0.61766800
C	10.73126800	-0.58675100	-0.38534700
H	9.43113300	0.16406000	-1.91825000
C	10.11334700	-2.68506700	0.64722100
C	10.97885000	-1.62233400	0.54851100

H	11.42704900	0.25207300	-0.45698900
H	10.30132700	-3.48652600	1.36632400
H	11.86211200	-1.57334300	1.18896700

3. References

[1] K. T. Schütt, O. T. Unke, M. Gastegger. "Equivariant Message Passing for the Prediction of Tensorial Properties and Molecular Spectra," In Proceedings of the 38th International Conference on Machine Learning (ICML); PMLR, (2021); Vol. 139, pp 9377–9388.

[2] W. Kohn, L. J. Sham, "Self-Consistent Equations Including Exchange and Correlation Effects," *Physical Review* **140**, (1965): A1133-A1138.

[3] P. Hohenberg, W. Kohn, "Inhomogeneous Electron Gas," *Physical Review* **136**, (1964)**:** B864-B871.

[4] J. D. Chai, M. Head-Gordon, "Long-range corrected hybrid density functionals with damped atom-atom dispersion corrections," *Phys. Chem. Chem. Phys.* **10**, (2008): 6615-20.

[5] J. D. Chai, M. Head-Gordon, "Accurate Coulomb-fitting basis sets for H to Rn," *Phys. Chem. Chem. Phys.* **8**, (2006): 1057-65.

[6] F. Weigend, R. Ahlrichs, "Balanced basis sets of split valence, triple zeta valence and quadruple zeta valence quality for H to Rn: Design and assessment of accuracy," *Phys. Chem. Chem. Phys.* **7**, (2005): 3297-305.

[7] L. C. Menikarachchi, J. A. Gascon, "QM/MM approaches in medicinal chemistry research," *Curr. Top. Med. Chem.* **10**, (2010): 46-54.

[8] H. M. Senn, W. Thiel, "QM/MM methods for biomolecular systems," *Angew. Chem. Int. Ed. Engl.* **48**, (2009): 1198-229.

[9] S. Dapprich, I. Komáromi, K. S. Byun, K. Morokuma, M. J. Frisch, "A new ONIOM implementation in Gaussian98. Part I. The calculation of energies, gradients, vibrational frequencies and electric field derivatives," *Journal of Molecular Structure: THEOCHEM* **461-462**, (1999): 1-21.

[10] T. Vreven, K. S. Byun, I. Komaromi, S. Dapprich, J. A. Montgomery, K. Morokuma, M. J. Frisch, "Combining Quantum Mechanics Methods with Molecular Mechanics Methods in ONIOM," *J. Chem. Theory. Comput.* **2**, (2006): 815-26.

[11] L. W. Chung, W. M. Sameera, R. Ramozzi, A. J. Page, M. Hatanaka, G. P. Petrova, T. V. Harris, X. Li, Z. Ke, F. Liu, H. B. Li, L. Ding, K. Morokuma, "The ONIOM Method and Its Applications," *Chem. Rev.* **115**, (2015): 5678-796.

[12] A. K. Rappe, C. J. Casewit, K. S. Colwell, W. A. Goddard, W. M. Skiff, "UFF, a full periodic table force field for molecular mechanics and molecular dynamics simulations," *Journal of the American Chemical Society* **114**, (1992): 10024-10035.

[13] J. Tomasi, B. Mennucci, R. Cammi, "Quantum mechanical continuum solvation models," *Chem. Rev.* **105**, (2005): 2999-3093.

[14] S. Miertuš, E. Scrocco, J. Tomasi, "Electrostatic interaction of a solute with a continuum. A direct utilizaion of AB initio molecular potentials for the prevision of solvent effects," *Chemical Physics* **55**, (1981): 117-129.

[15] M. Cossi, V. Barone, R. Cammi, J. Tomasi, "Ab initio study of solvated molecules: a new implementation of the polarizable continuum model," *Chemical Physics Letters* **255**, (1996): 327-335.

[16] Gaussian 16, Revision C.01, M. J. Frisch, G. W. Trucks, H. B. Schlegel, G. E. Scuseria, M. A. Robb, J. R. Cheeseman, G. Scalmani, V. Barone, G. A. Petersson, H. Nakatsuji, X. Li, M. Caricato, A. V. Marenich, J. Bloino, B. G. Janesko, R. Gomperts, B. Mennucci, H. P. Hratchian, J. V. Ortiz, A. F.

Izmaylov, J. L. Sonnenberg, D. Williams-Young, F. Ding, F. Lipparini, F. Egidi, J. Goings, B. Peng, A. Petrone, T. Henderson, D. Ranasinghe, V. G. Zakrzewski, J. Gao, N. Rega, G. Zheng, W. Liang, M. Hada, M. Ehara, K. Toyota, R. Fukuda, J. Hasegawa, M. Ishida, T. Nakajima, Y. Honda, O. Kitao, H. Nakai, T. Vreven, K. Throssell, J. A. Montgomery, Jr., J. E. Peralta, F. Ogliaro, M. J. Bearpark, J. J. Heyd, E. N. Brothers, K. N. Kudin, V. N. Staroverov, T. A. Keith, R. Kobayashi, J. Normand, K. Raghavachari, A. P. Rendell, J. C. Burant, S. S. Iyengar, J. Tomasi, M. Cossi, J. M. Millam, M. Klene, C. Adamo, R. Cammi, J. W. Ochterski, R. L. Martin, K. Morokuma, O. Farkas, J. B. Foresman, and D. J. Fox, Gaussian, Inc., Wallingford CT, (2016).

[17] Legault, C. Y., CYL view, 1.0b; Universite de Sherbrooke (2009), http://www.cylview.org (accessed Oct 11, 2022).

[18] W. Humphrey, A. Dalke, K. Schulten, "VMD: Visual molecular dynamics," *J. Mol. Graphics* **14**, (1996): 33–38.
